# Supplementary material for: The human Y and inactive X chromosomes similarly modulate autosomal gene expression
Source: Cell Genom. 2023 Dec 13;4(1):100462. doi: 10.1016/j.xgen.2023.100462 (PMC10794785; doi:10.1016/j.xgen.2023.100462)
Supplement: Document S1. Figures S1–S22 [file mmc1.pdf]

Cell Genomics, Volume *4*

## Supplemental information

# The human Y and inactive X chromosomes similarly modulate autosomal gene expression

**Adrianna K. San Roman, Helen Skaletsky, Alexander K. Godfrey, Neha V. Bokil, Levi Teitz, Isani Singh, Laura V. Blanton, Daniel W. Bellott, Tatyana Pyntikova, Julian Lange, Natalia Koutseva, Jennifer F. Hughes, Laura Brown, Sidaly Phou, Ashley Buscetta, Paul Kruszka, Nicole Banks, Amalia Dutra, Evgenia Pak, Patricia C. Lasutschinkow, Colleen Keen, Shanlee M. Davis, Angela E. Lin, Nicole R. Tartaglia, Carole Samango-Sprouse, Maximilian Muenke, and David C. Page**

## Figure S1

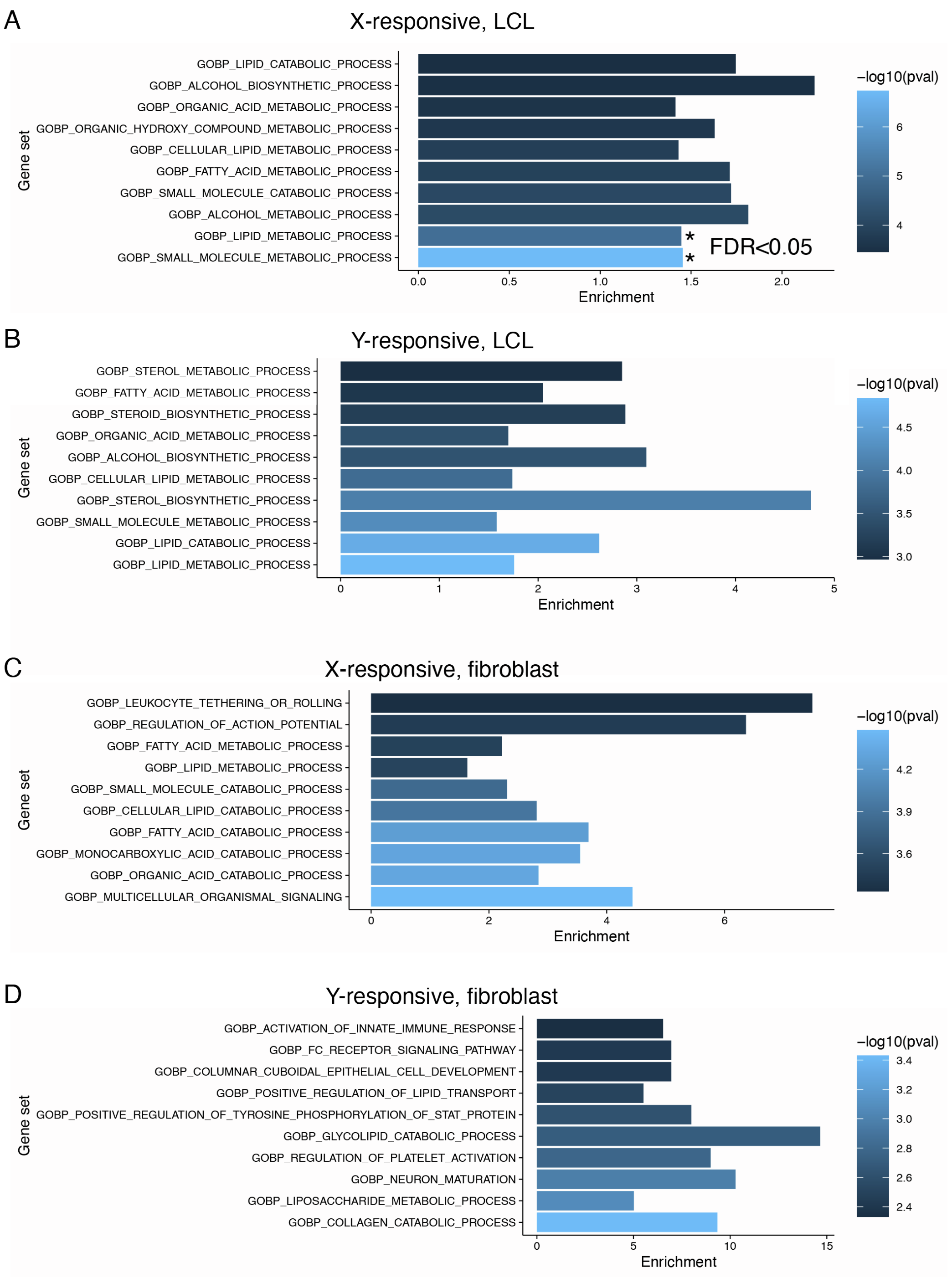


**Figure S1. Analysis of gene ontology category enrichment in autosomal genes that significantly respond to Chr X or Y copy number, related to Figure 1.** Enrichment of

“Biological Process” Gene Ontology categories was calculated separately for autosomal genes that significantly responded to Chr X or Y copy number in LCLs or fibroblasts using Fisher’s exact test. All expressed genes in LCLs or fibroblasts were used as the background set. Shown here are the top 10 enriched categories based on P value. Stars indicate those that were below the Benjamini-Hochberg-corrected P value (FDR) of 0.05. There were no significant categories (by FDR threshold) for genes that responded to Chr Y copy number in LCLs, nor for genes that responded to Chr X or Y copy number in fibroblasts.

**Figure S2**

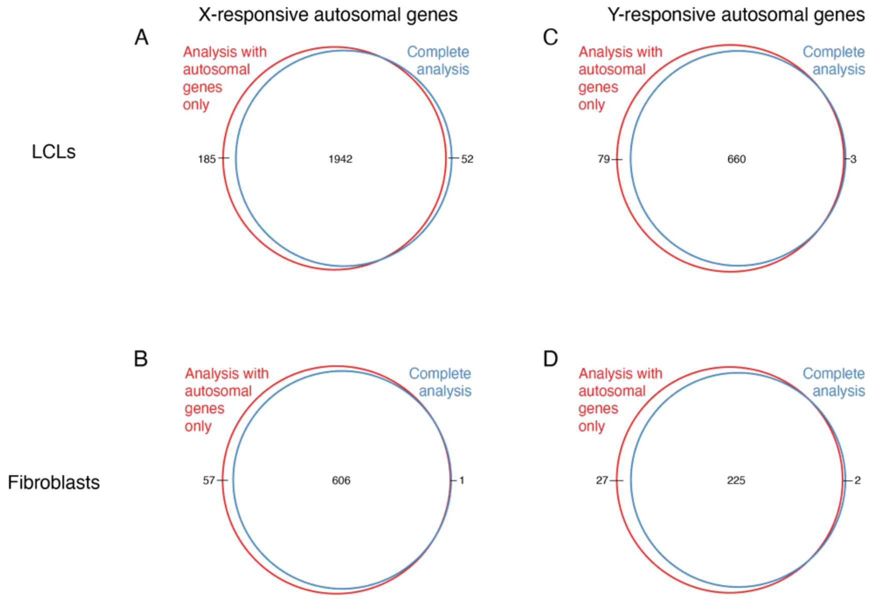


**Figure S2. Mapping to a transcriptome with or without the sex chromosomes does not affect X- and Y-responsive autosomal genes in LCLs and fibroblasts, related to Figure 1.** Read depth normalization between samples and differential expression was performed using an annotation only containing autosomal genes to rule out artifacts of bulk over-expression of sex chromosome genes, and compared to the analysis conducted using the complete annotation. Significantly X-responsive genes in LCLs (A) and fibroblasts (B) were almost completely overlapping between the two analyses, as were the significantly Y-responsive genes in LCLs (C) and fibroblasts (D).

**Figure S3**

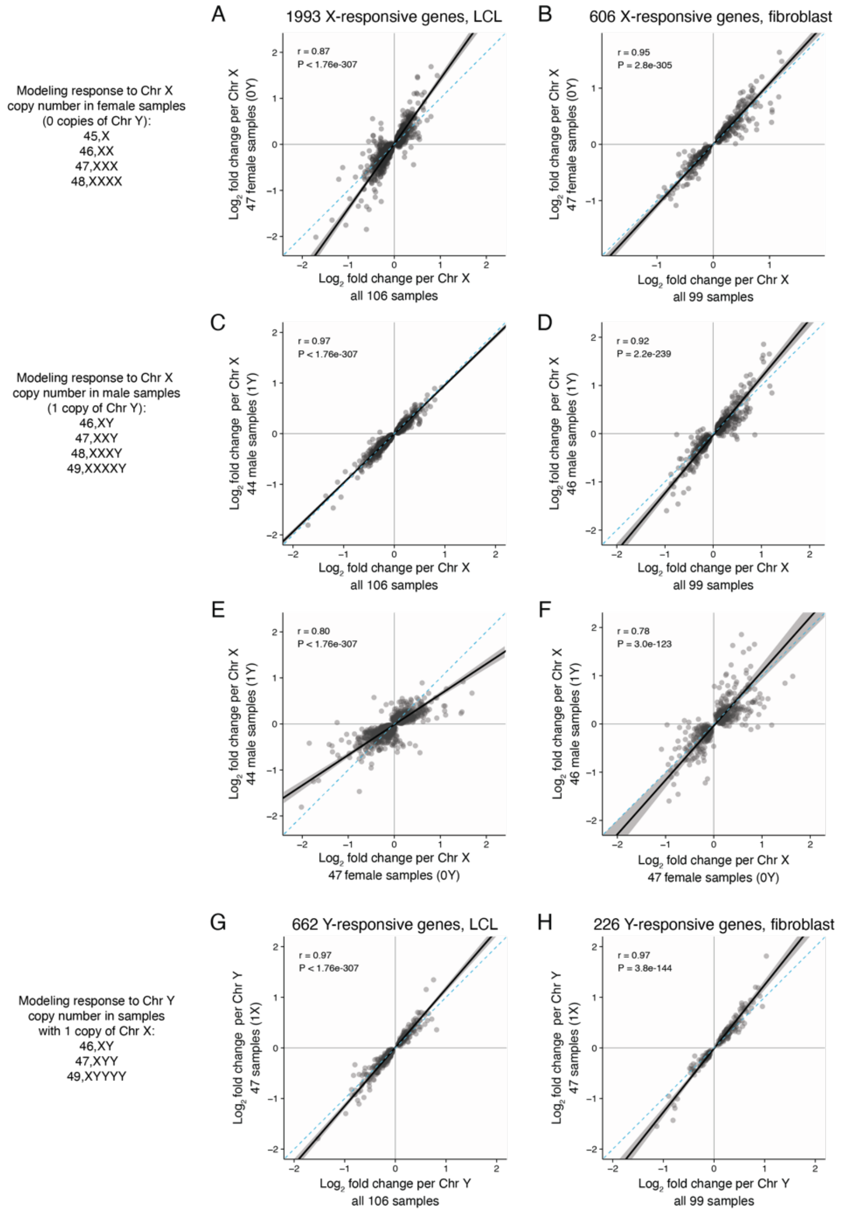


**Figure S3. Modeling Chr X or Y copy number separately is highly correlated with models including both covariates, related to Figure 1. (A-D)** Scatterplots comparing models of gene expression as a function of Chr X copy number in female samples (zero Y chromosomes; [A, B]) or male samples with one Y chromosome (C, D) with the full model show high correlations. **(E, F)** Scatterplots comparing models of gene expression as a function of Chr X copy number in male samples with one Y chromosome and female samples (no Y chromosomes) show high

correlations. **(G, H)** Scatterplots comparing a model of gene expression as a function of Chr Y copy number in male samples with one X chromosome with the full model reveal a high correlation. Black line and grey shading, Deming regression and 95% confidence interval; blue dashed line, identity (X=Y) line. Pearson correlation coefficients and Bonferroni-adjusted P values are indicated.

**Figure S4**

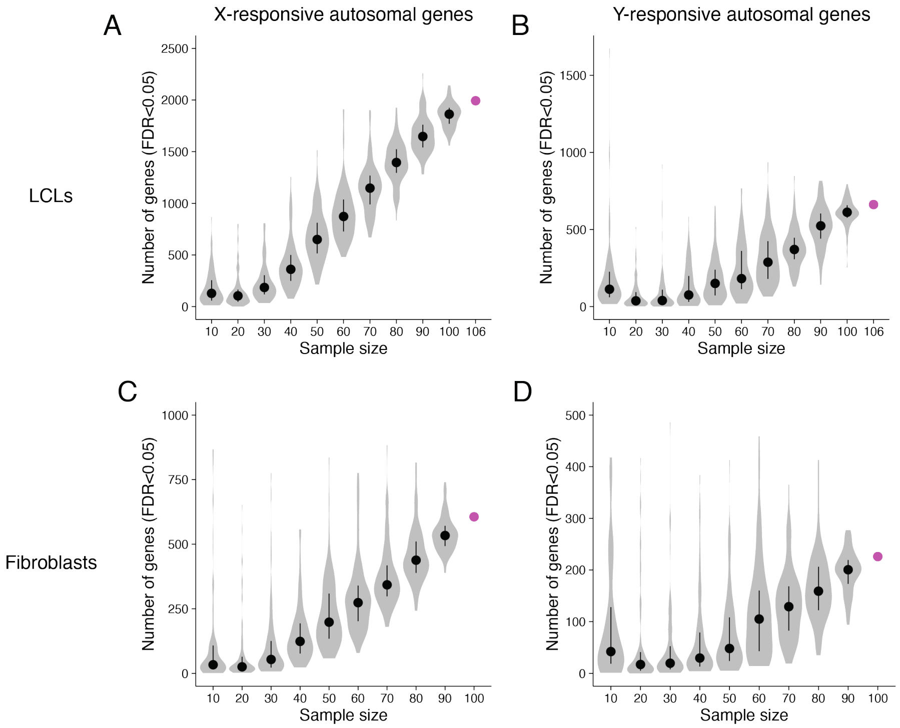


**Figure S4. Detection of autosomal genes significantly responsive to Chr X and Y copy number is not saturated at the current sample size, related to Figure 1.** To determine whether we had saturated the signal of significantly Chr X- or Y-responsive genes at the given sample size, we performed a bootstrapping analysis. Violin plots with median (dots) and interquartile range (whiskers) showing the number of Chr X-responsive (**A, C**) or Y-responsive (**B, D**) autosomal genes detected in random subsets of the LCL (A,B) or fibroblast (C,D) RNA-seq libraries. The number of samples in each subset is shown on the x-axis. 100 random samples were obtained at each sample size. Pink dots show the number of Chr X- and Y-responsive genes detected in the full set of samples. The large number of genes detected in some of the smallest subsets is caused by unbalanced karyotype distributions.

# Figure S5

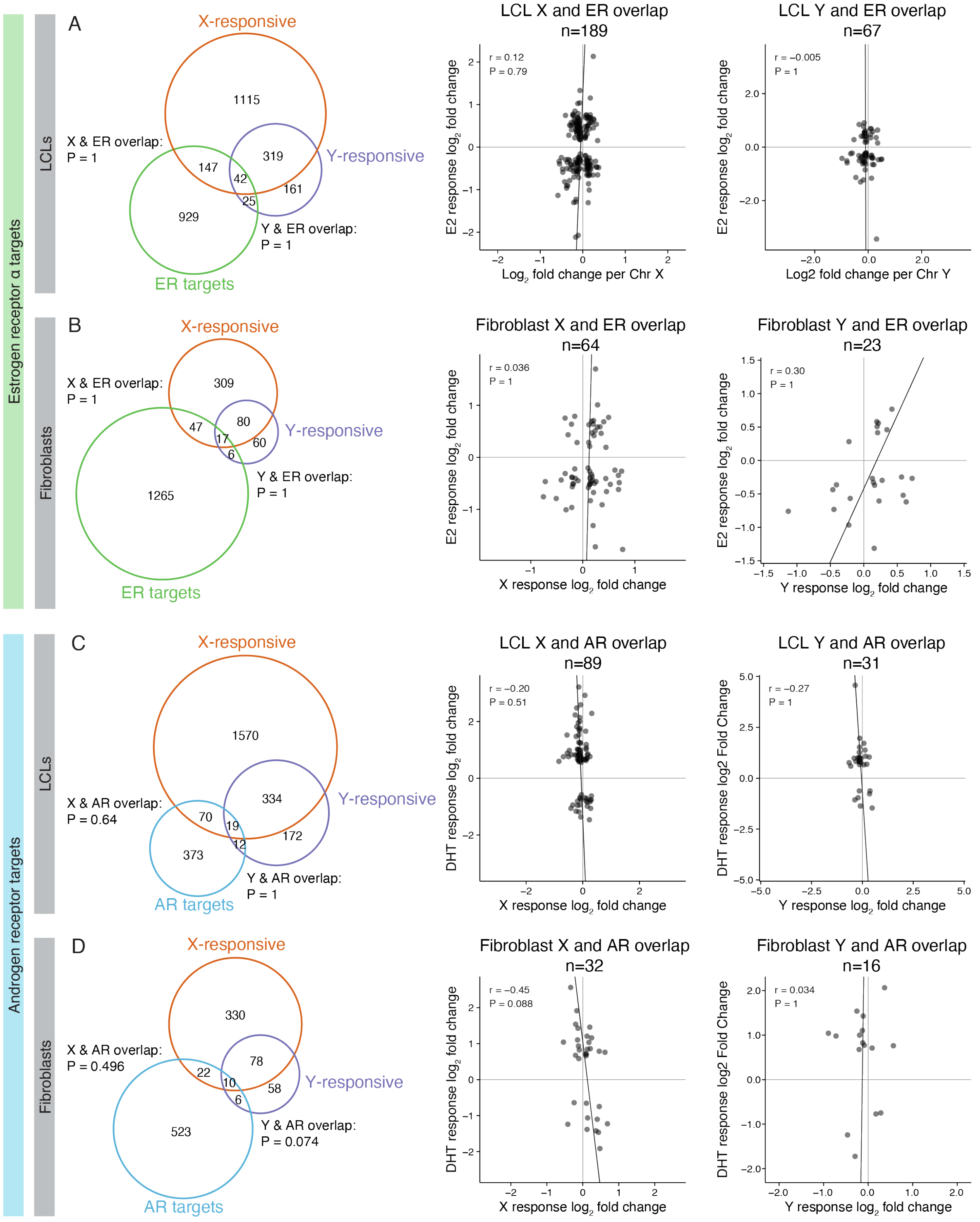


**Figure S5. The response to Chr X- and Y-responsive genes is distinct from the response to sex hormones, as defined by estrogen receptor alpha and androgen receptor direct target**

**genes, related to Figure 1. (A-B)** *Left,* Venn diagrams showing overlap of ERα direct target genes (bound by ERα within 30 kb of the transcription start site and differentially expressed [$P_{adj}$<0.05] with 17-ß estradiol [E2] treatment) and Chr X- or Y-responsive genes in LCLs (A) or fibroblasts (B). Venn diagrams were restricted to autosomal genes expressed in both MCF7 cells (where ERα direct targets were defined) and LCLs or fibroblasts. *Right,* scatterplots of overlapping genes showing the relationship between response to E2 and Chr X or Y copy number. **(C-D)** *Left,* Venn diagrams showing overlap of AR direct target genes (bound by AR within 30 kb of the transcription start site and differentially expressed [$P_{adj}$<0.05] with dihydrotestosterone [DHT] treatment) and Chr X- or Y-responsive genes in LCLs (C) or fibroblasts (D). Venn diagrams were restricted to autosomal genes expressed in both LNCaP cells (where AR direct targets were defined) and LCLs or fibroblasts. *Right,* scatterplots of overlapping genes showing the relationship between response to DHT and Chr X or Y copy number. Venn diagram Bonferroni-adjusted P values, hypergeometric test; scatterplots show Deming regressions (black lines), Pearson correlation coefficients and Bonferroni-adjusted P values.

## Figure S6

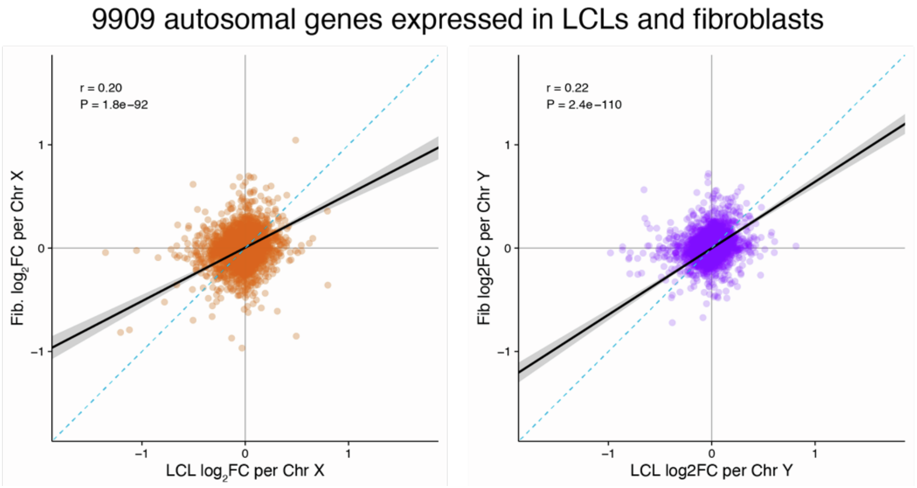


**Figure S6. Response to Chr X or Y copy number is weakly correlated between cell types, related to Figure 1.** Scatterplots comparing the effect sizes of the response to Chr X (left) or Y (right) copy number in LCLs versus fibroblasts for all autosomal genes expressed in both cell types. Black line and grey shading, weighted Deming regression and 95% confidence interval; blue dashed line, identity (X=Y) line; Pearson correlation coefficients and Bonferroni-adjusted P values are indicated.

**Figure S7**

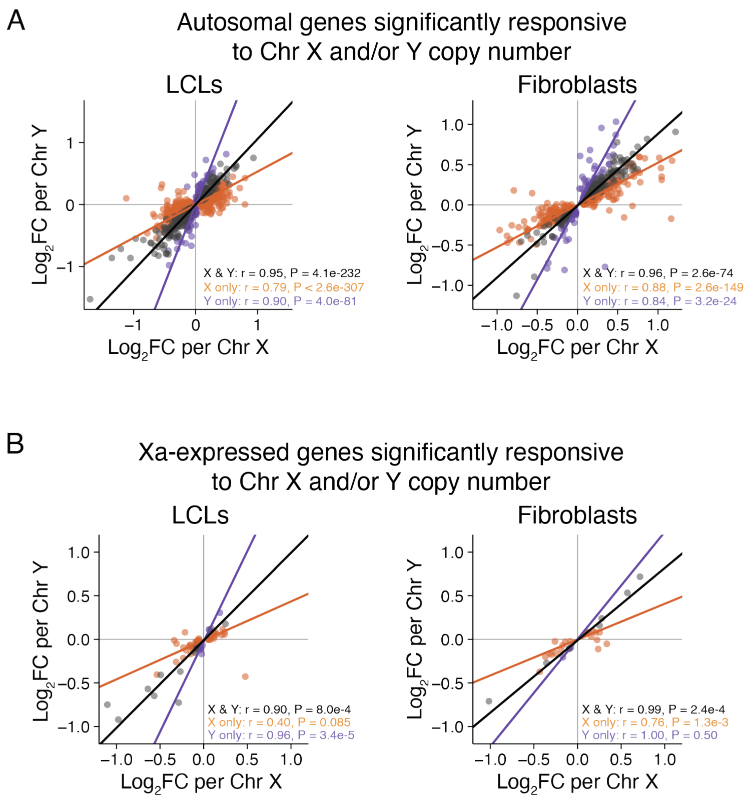


**Figure S7. Correlation plots for significantly X and/or Y responsive genes, related to Figure 2.** Scatterplots showing the relationship between the effects per additional copy of Chr X or Y for autosomal genes **(A)** or Xa-expressed genes **(B)** called as significantly responsive ($P_{adj}$<0.05) to either Chr X, Chr Y, or both. Weighted Deming regression lines, Pearson correlation coefficients, and Bonferroni-adjusted P values of the correlation are included in each plot.

## Figure S8

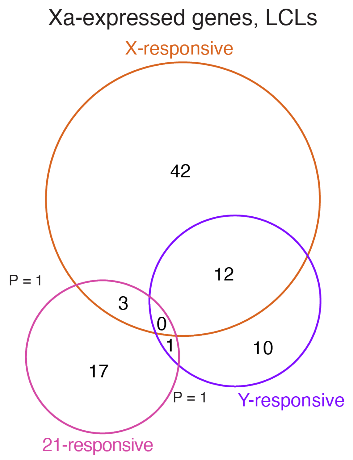


**Figure S8. For Xa-expressed genes, the response to Chr 21 copy number is distinct from the shared response to Chr X and Chr Y copy number, related to Figure 3.** Venn diagram of Xa-expressed genes that significantly respond to Chr 21, Chr X, or Chr Y copy number. This analysis is limited to genes previously annotated as transcribed from Xa but not from Xi. Bonferroni-adjusted P values, hypergeometric test.

**Figure S9**

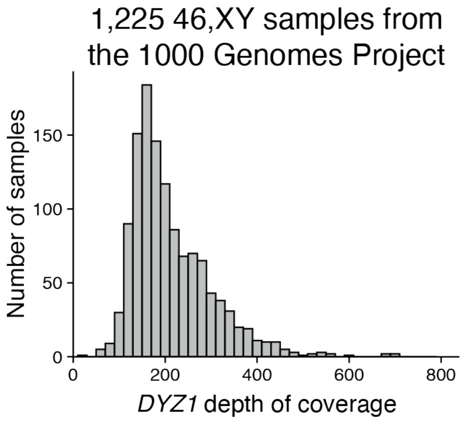


**Figure S9. Wide range of Y heterochromatin lengths in males from the 1000 Genomes Project, related to Figure 4.** Histogram of *DYZ1* depth of coverage – a proxy for Chr Y heterochromatin length – for 1,225 46,XY samples from the 1000 Genomes Project.

**Figure S10**

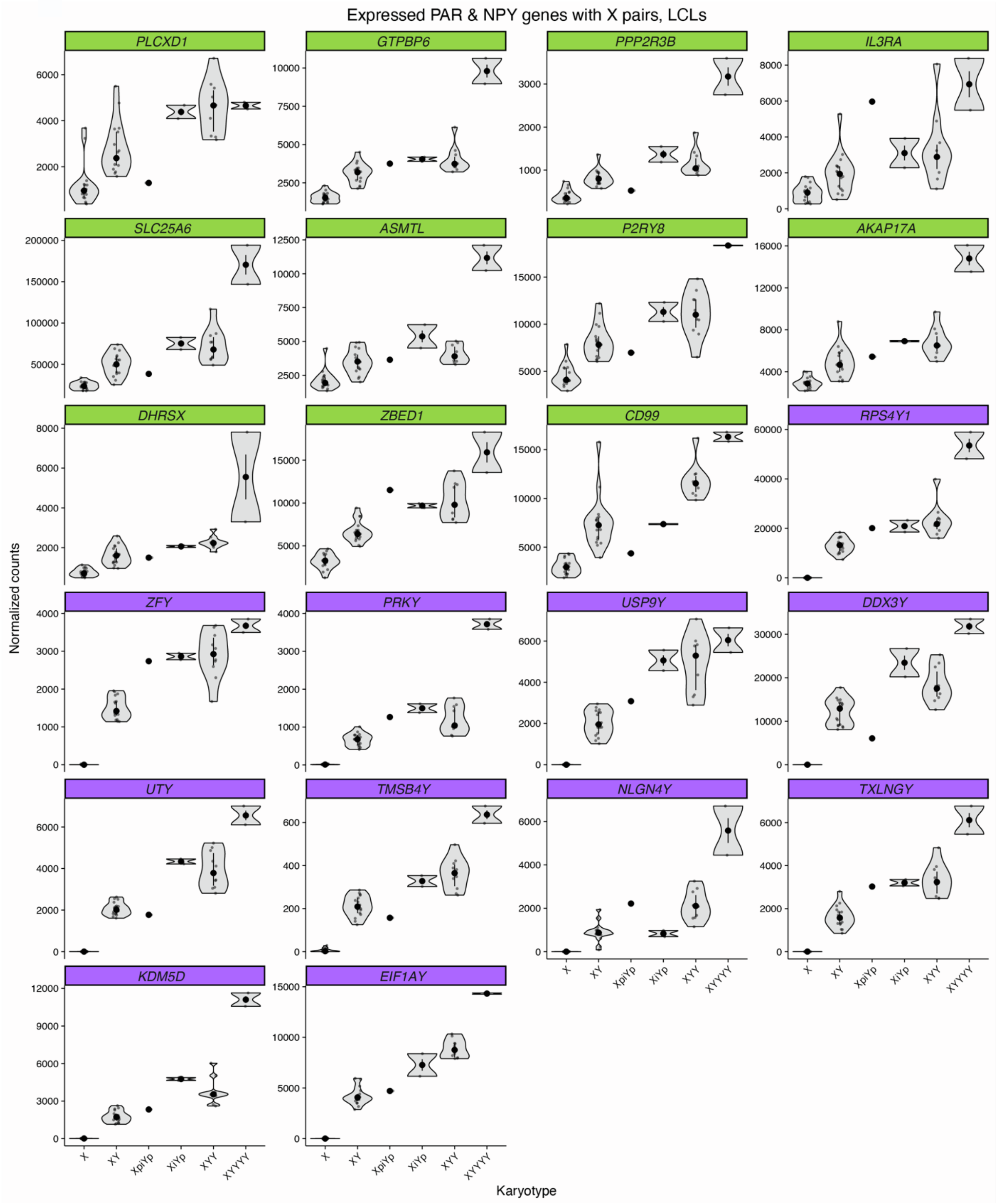

Expressed PAR & NPY genes with X pairs, LCLs
PLCXD1
GTPBP6
PPP2R3B
IL3RA
SLC25A6
ASMTL
P2RY8
AKAP17A
DHRSX
ZBED1
CD99
RPS4Y1
ZFY
PRKY
USP9Y
DDX3Y
UTY
TMSB4Y
NLGN4Y
TXLNGY
KDM5D
EIF1AY
Normalized counts
X
XY
XpiYp
XiYp
XYY
XYYYY
Karyotype

**Figure S10. Chr Y gene expression in samples with variant Y chromosomes, related to Figure 4.** Violin plots showing median and interquartile range for all expressed Chr Y genes (PAR1 and NPY) for samples with two types of variant Y chromosomes: isoYp (XiYp) with recombination in palindrome P1, pseudoisoYp (XpiYp) with recombination between IR4 inverted repeats, or samples with zero to four copies of Chr Y and one copy of Chr X. Green, PAR1 genes; purple, NPY genes with homologs on Chr X.

## Figure S11

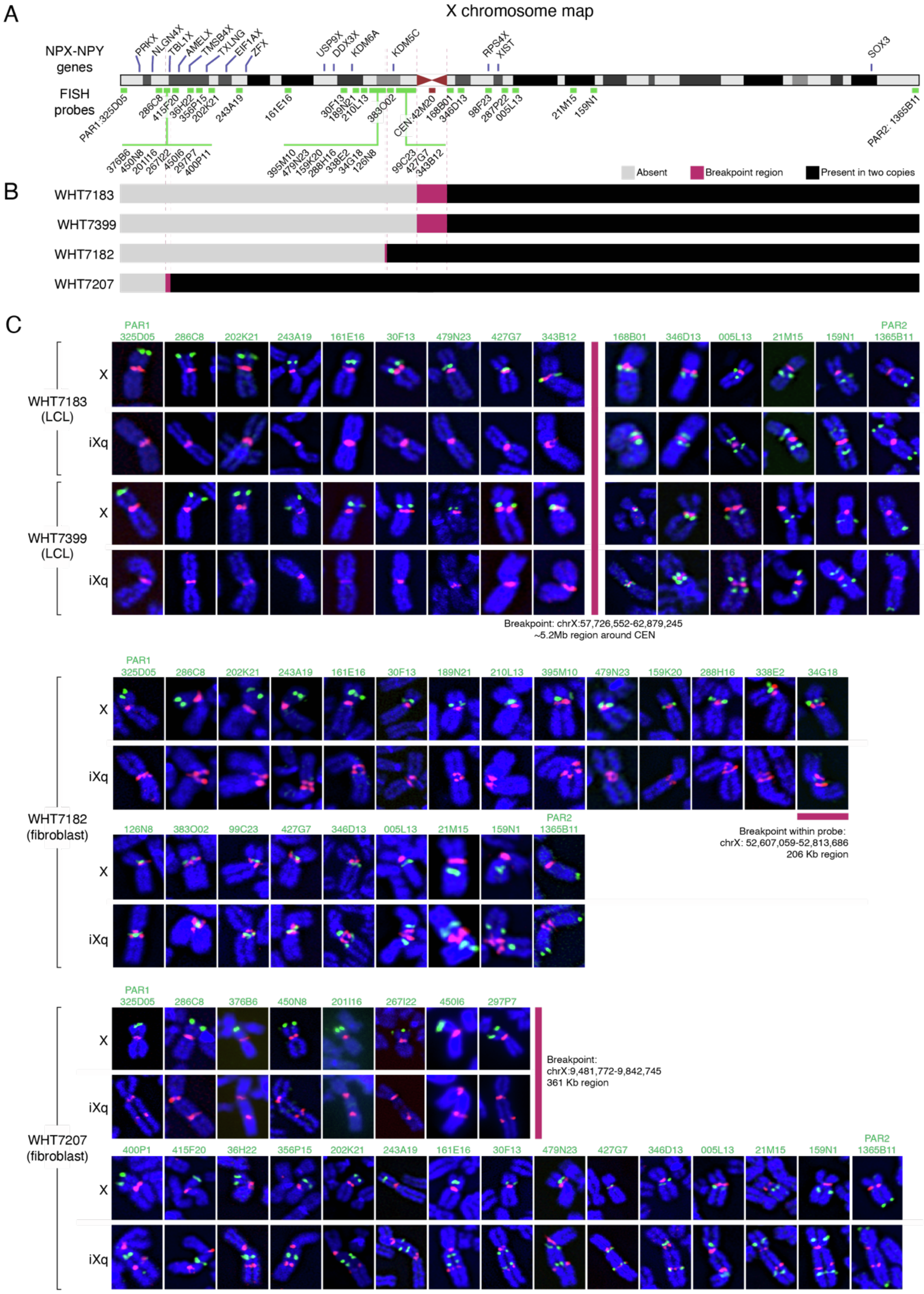
A
X chromosome map
NPX-NPY genes
PRKX
NLGN4X
TBL1X
AMELX
TMSB4X
TXLNG
EIF1AX
ZFX
USP9X
DDX3X
KDM6A
KDM5C
RPS4X
XIST
SOX3
FISH probes
PAR1:325D05
286C8
415F20
36H22
356P15
202K21
243A19
161E16
30F13
189N21
210L13
383O02
CEN:42M20
168B01
346D13
98F23
287P22
005L13
21M15
159N1
PAR2: 1365B11
376B6
450N8
201I16
267I22
450I6
297P7
400P11
395M10
479N23
159K20
288H16
338E2
34G18
126N8
99C23
427G7
343B12
Absent
Breakpoint region
Present in two copies
B
WHT7183
WHT7399
WHT7182
WHT7207
C
WHT7183 (LCL)
WHT7399 (LCL)
X
iXq
Breakpoint: chrX:57,726,552-62,879,245
~5.2Mb region around CEN
WHT7182 (fibroblast)
Breakpoint within probe:
chrX: 52,607,059-52,813,686
206 Kb region
WHT7207 (fibroblast)
Breakpoint:
chrX:9,481,772-9,842,745
361 Kb region
400P1

**Figure S11. Determination of X isochromosome breakpoints in four individuals using DNA FISH, related to Figure 5. (A)** Chr X ideogram showing locations of NPX-NPY gene pairs and FISH probes. Centromere probe 42M20 (labeled in red) served as a point of reference in all experiments; all other probes labeled in green. **(B)** Locations of breakpoints on X isochromosomes as determined by FISH. Locations of breakpoints correspond to schematic of X Chr in (A) and are indicated with pink shading. Grey, region absent in iXq; pink, breakpoint region; black, region present in two copies in iXq. **(C)** Breakpoints for iXq chromosomes were refined using DNA FISH. For each individual and probe, an image of a normal Chr X and iXq are included from the same chromosome spread for comparison. Images for WHT7183 and WHT7399 were obtained from FISH on spreads from LCLs, while those for WHT7182 and WHT7202 were obtained from FISH on spreads from fibroblasts. See Table S7 for additional details of gene content and FISH probes.

## Figure S12

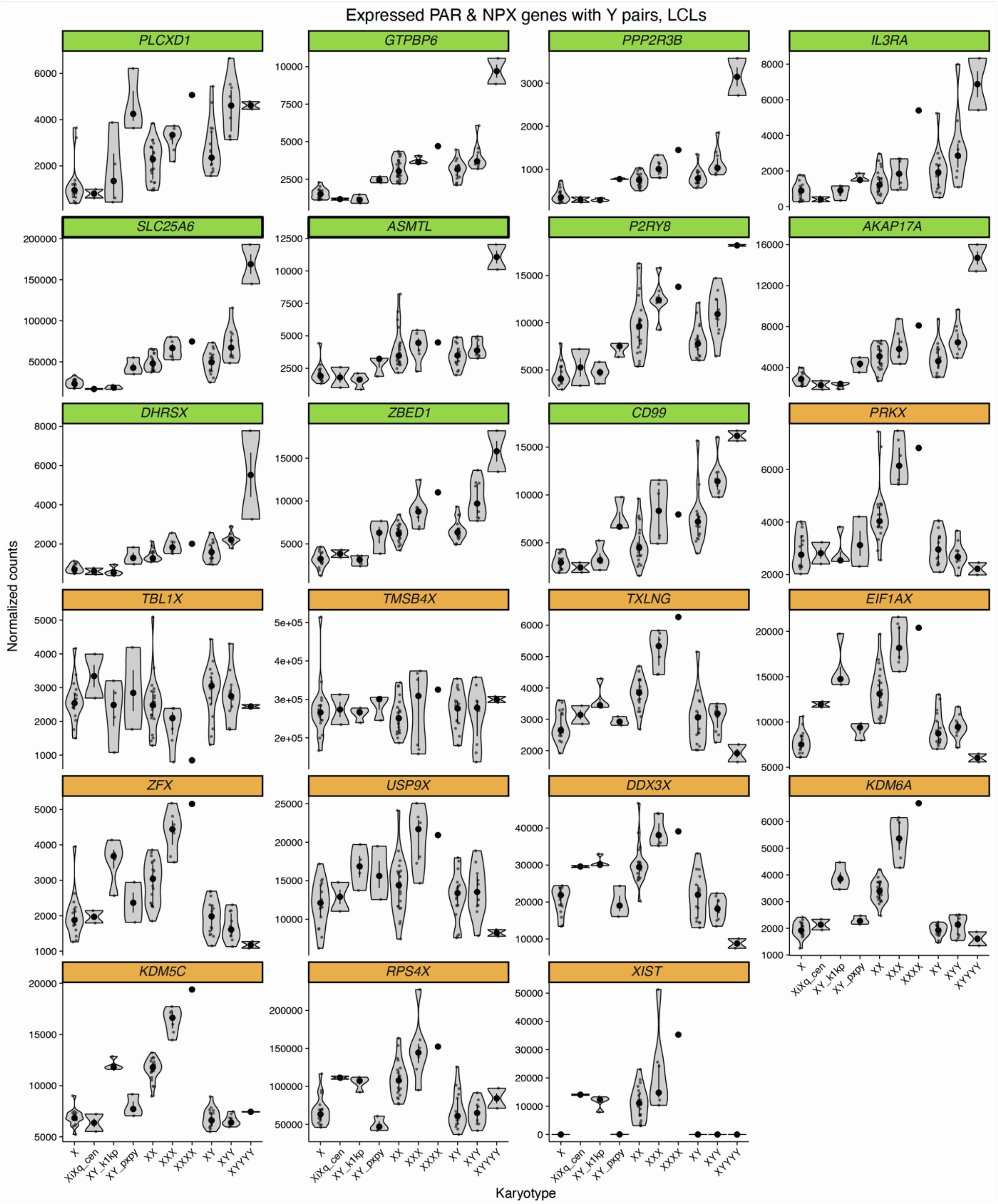

Expressed PAR & NPX genes with Y pairs, LCLs
PLCXD1
GTPBP6
PPP2R3B
IL3RA
SLC25A6
ASMTL
P2RY8
AKAP17A
DHRSX
ZBED1
CD99
PRKX
TBL1X
TMSB4X
TXLNG
EIF1AX
ZFX
USP9X
DDX3X
KDM6A
KDM5C
RPS4X
XIST
Normalized counts
Karyotype
X
XiXq_cen
XY_k1kp
XY_pxpy
XX
XXX
XXXX
XY
XYY
XYYYY

**Figure S12. Expression levels of PAR1 and NPX genes with NPY homologs in LCL samples with structural variants of Chr X or Chr Y, related to Figure 5.** Violin plots showing median and interquartile range for expressed PAR1 genes (green) and NPX genes with NPY homologs and *XIST* (orange) for samples with Chr X or Y structural variants or samples with one to four copies of Chr X and one to four copies of Chr Y. Karyotypes with only one sample are represented as a single dot. XiXq_cen: 46,X,i(Xq) samples with recombination in the centromere; XY_k1kp: 46,X,t(X;Y)(*ANOS1-ANOS2P*); XY_pxpy: 46,X,t(X;Y)(*PRKX-PRKY*).

## Figure S13

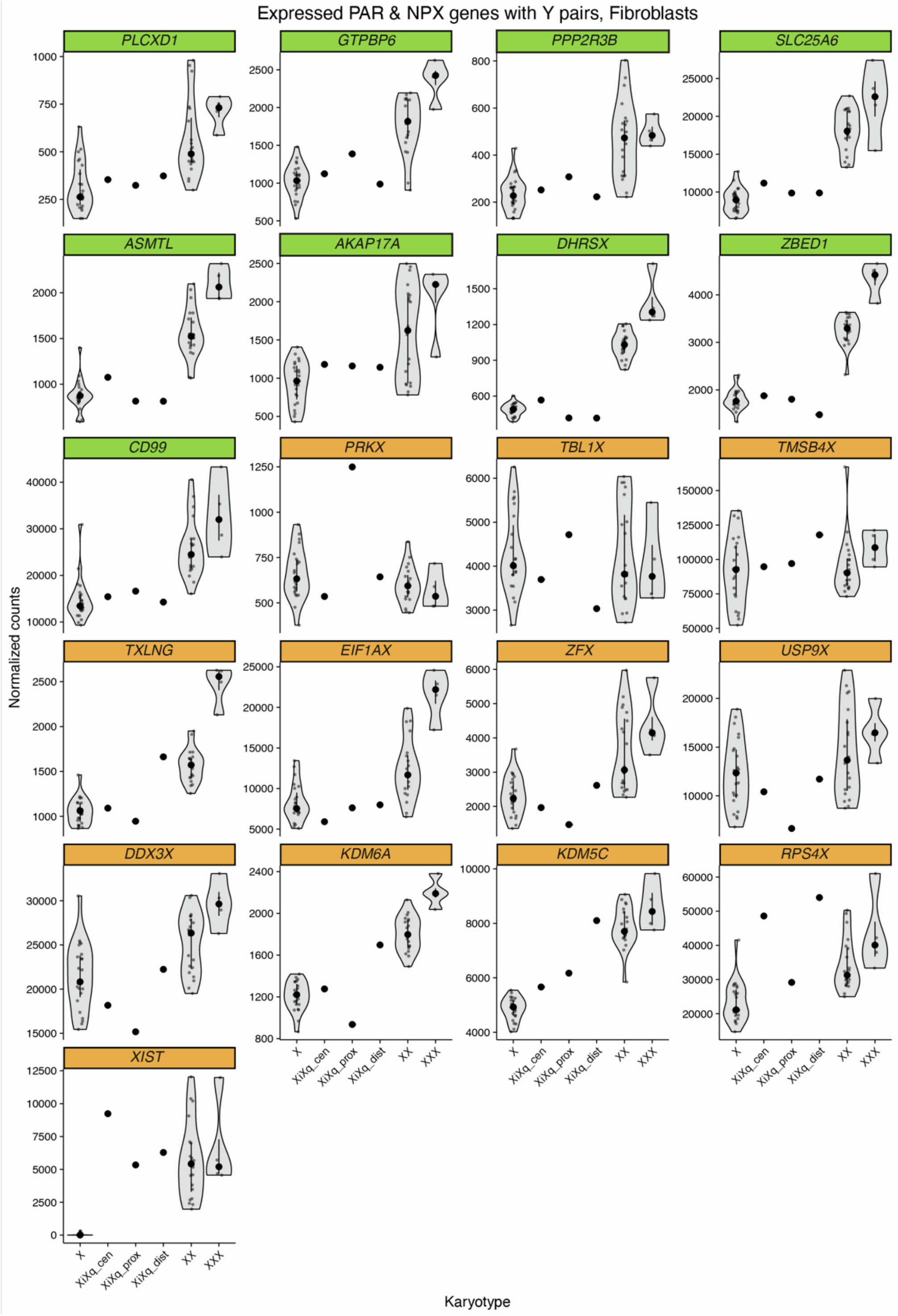
Expressed PAR & NPX genes with Y pairs, Fibroblasts
PLCXD1
GTPBP6
PPP2R3B
SLC25A6
ASMTL
AKAP17A
DHRSX
ZBED1
CD99
PRKX
TBL1X
TMSB4X
TXLNG
EIF1AX
ZFX
USP9X
DDX3X
KDM6A
KDM5C
RPS4X
XIST
Normalized counts
X
XiXq_cen
XiXq_prox
XiXq_dist
XX
XXX
Karyotype

**Figure S13. Expression levels of PAR1 and NPX genes with NPY homologs in fibroblast samples with structural variants of Chr X, related to Figure 5.** Violin plots showing median and interquartile range for expressed PAR1 genes (green) and NPX genes with NPY homologs and *XIST* (orange) for samples with Chr X structural variants or samples with one to three copies of Chr X. Karyotypes with only one sample are represented as a single dot. XiXq_cen: 46,X,i(Xq) samples with recombination in the centromere; XiXq_prox: 46,X,i(Xq) samples with recombination in proximal Xp; XiXq_dist: 46,Xi(Xq) samples with recombination in distal Xp.

## Figure S14

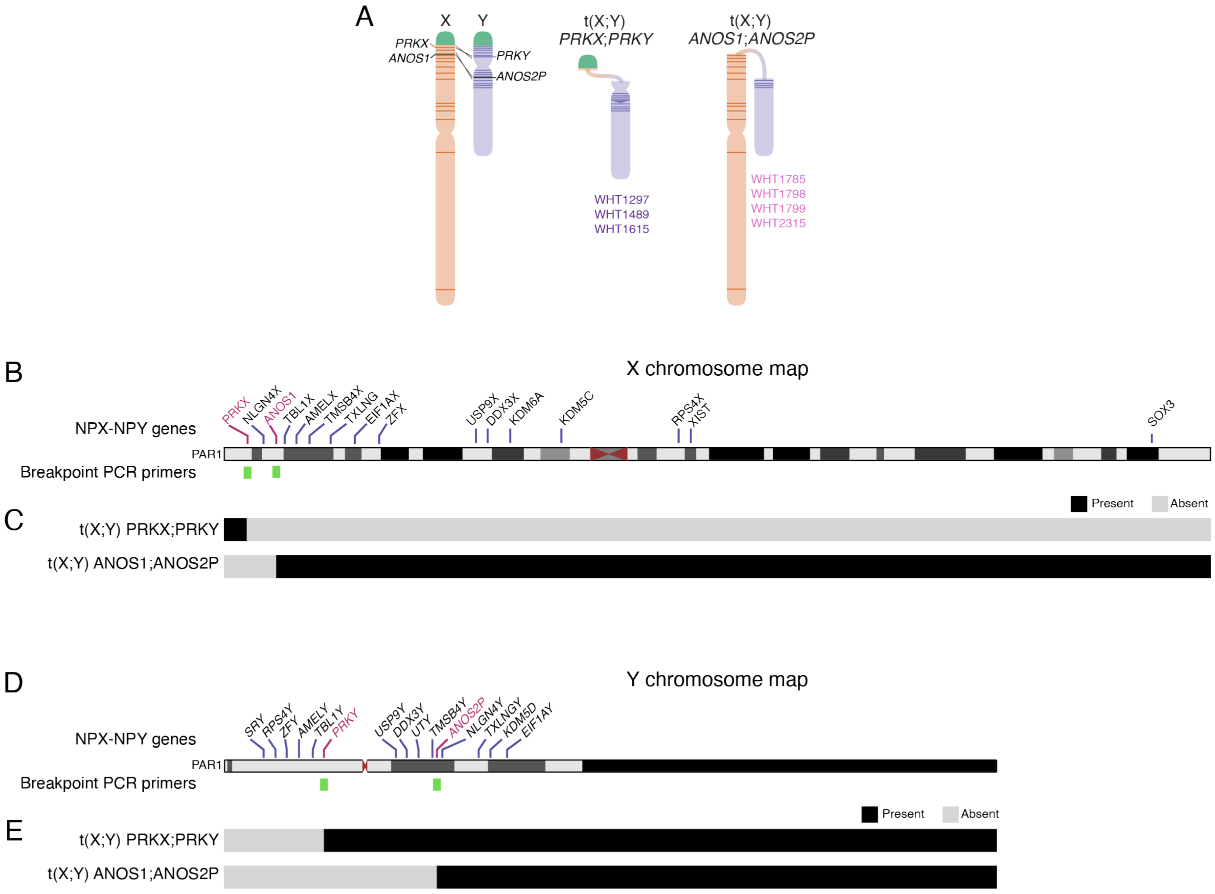


**Figure S14. X-Y translocation breakpoints in seven individuals, related to Figure 5. (A)** Schematic showing recombination between X and Y Chrs at *PRKX* and *PRKY*, or at *ANOS1* and *ANOS2P*, resulting in X-Y translocation products. **(B,D)** Chr X (B) and Chr Y (D) ideograms showing locations of NPX-NPY gene pairs, *ANOS1,* and *ANOS2P*. The locations of primers to amplify the breakpoints are indicated; for more details see Table S7. **(C,E)** Locations of breakpoints on the X (C) and Y (E) chromosomes as determined by PCR. Locations of breakpoints correspond to schematic of corresponding chromosomes above. Grey, region absent in t(X;Y) chromosome; black, region present in t(X;Y) chromosome.

## Figure S15

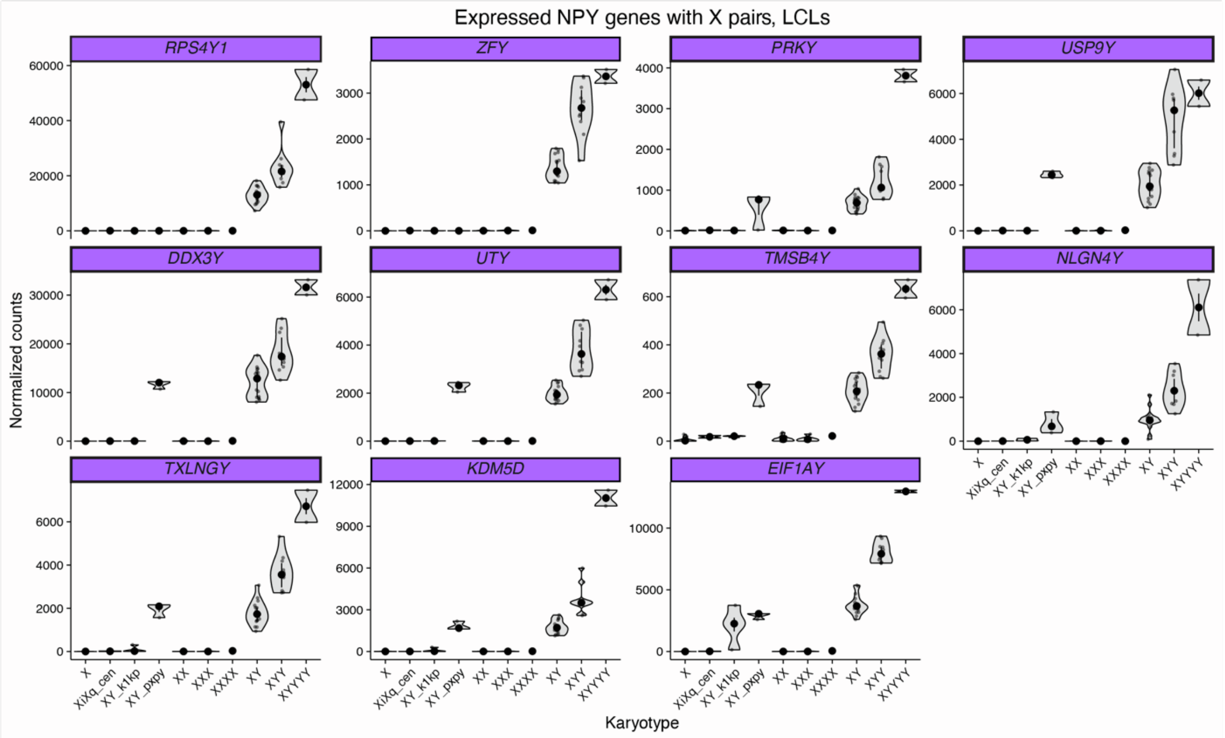


**Figure S15. Expression levels of NPY genes with NPX homologs in LCL samples with structural variants of Chr X or Chr Y, related to Figure 5.** Violin plots showing median and interquartile range for expressed NPY genes for samples with Chr X or Y structural variants or samples with one to four copies of Chr X and one to four copies of Chr Y. Karyotypes with only one sample are represented as a single dot. XiXq_cen: 46,X,i(Xq) samples with recombination in the centromere; XY_k1kp: 46,X,t(X;Y)(*ANOS1-ANOS2P*); XY_pxpy: 46,X,t(X;Y)(*PRKX-PRKY*).

## Figure S16

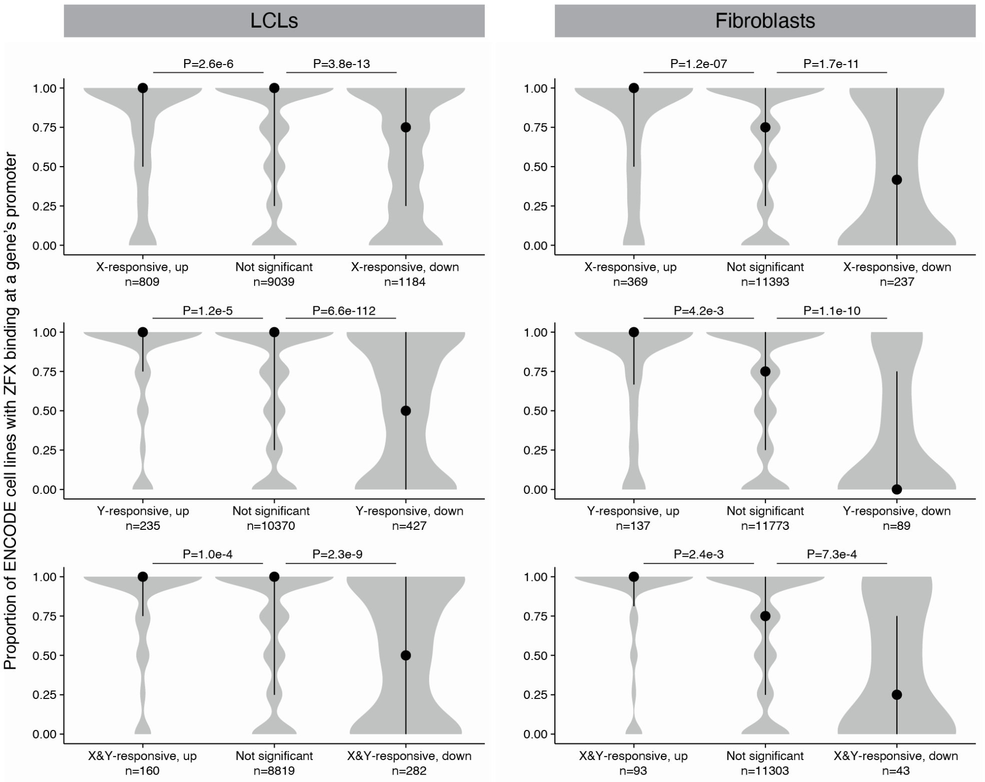


**Figure S16. ZFX protein binding is enriched in promoters of autosomal genes whose expression increases in response to Chr X and/or Y copy number, and is depleted in promoters of genes whose expression decreases, related to Figure 6.** For autosomal genes with or without significant expression changes with Chr X or Chr Y copy number, we investigated ZFX binding using publicly available ChIP-seq data from four cell lines. Violin plots, median (dot), and interquartile range (line) of the proportion of cell lines with ZFX binding at the gene's promoter (in cell lines where the gene is expressed). For example, a gene expressed in three of the four cell types with a ZFX peak in the promoter in those three cell types would have a proportion bound of 1. Top row, Chr X-responsive autosomal genes; middle row, Chr Y-responsive autosomal genes; bottom row, Chr X- and Y-responsive autosomal genes. The number of genes in each violin is indicated. Bonferroni-adjusted P values, Wilcoxon rank sum test.

**Figure S17**

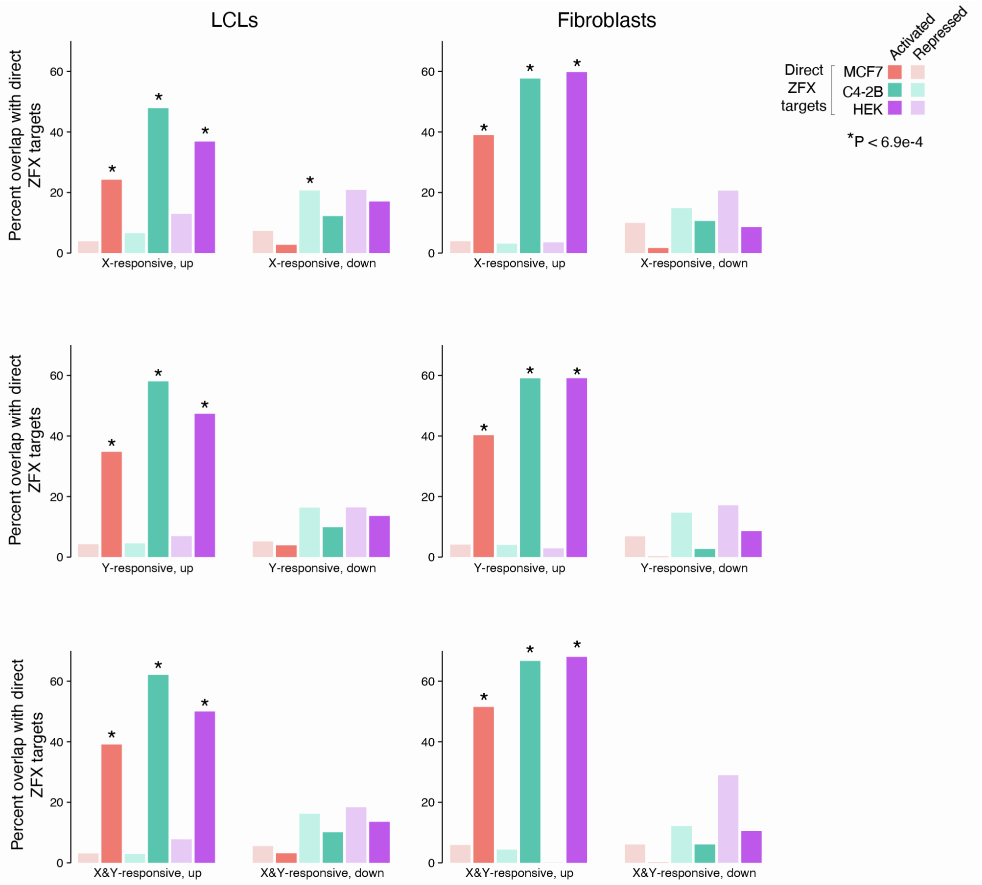


**Figure S17. Published ZFX-activated genes are enriched in autosomal genes positively responsive to Chr X and Y copy number, related to Figure 6.** Bar plots showing the percentage of genes responsive to Chr X and/or Y copy number that were identified as ZFX direct target genes in three cell types: MCF7, C4-2B, and HEK-293T [S1,2]. We defined direct target genes as those having a ZFX binding peak within 1 kb of the transcription start site and displaying significant expression changes (FDR<0.05) upon *ZFX* knockdown or knockout. We considered genes whose expression decreased with ZFX knockdown/knockout to be "activated" by ZFX, and those whose expression increased to be "repressed" by ZFX. Top row, Chr X-responsive autosomal genes; middle row, Chr Y-responsive autosomal genes; bottom row, Chr X- and Y-responsive autosomal genes. To identify significant enrichments of ZFX target genes in Chr X- or Y-responsive genes, we restricted analyses to genes expressed in both cell lines

(e.g., LCLs and MCF7 cells) and performed hypergeometric tests. Bars marked with an asterisk indicate the P value of the enrichment was lower than the Bonferroni-adjusted threshold of 6.9e-4.

**Figure 18**

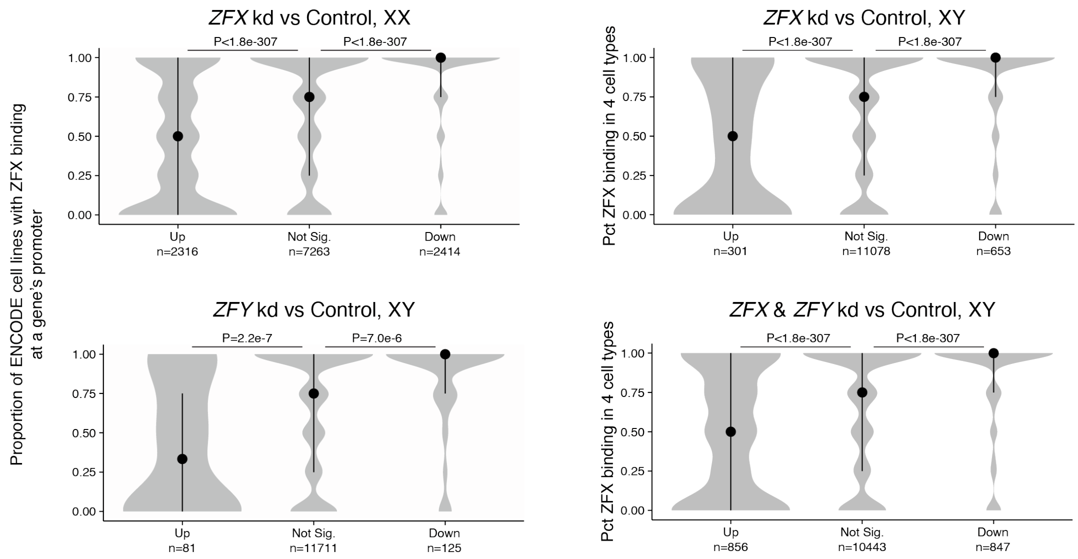


**Figure S18. ZFX protein binding is enriched in promoters of autosomal genes that decrease in response to *ZFX* and/or *ZFY* knockdown in fibroblasts, and is depleted in promoters of genes that increase, related to Figure 6.** For autosomal genes with or without significant expression changes in each of the four CRISPRi experiments, we investigated ZFX binding using publicly available ChIP-seq data from four cell lines (as in Fig. S16). Violin plots, median (dot), and interquartile range (line) of the proportion of cell lines with ZFX binding at the gene's promoter (in cell lines where the gene is expressed). The number of genes in each violin is indicated. Bonferroni-adjusted P values, Wilcoxon rank sum test.

**Figure S19**

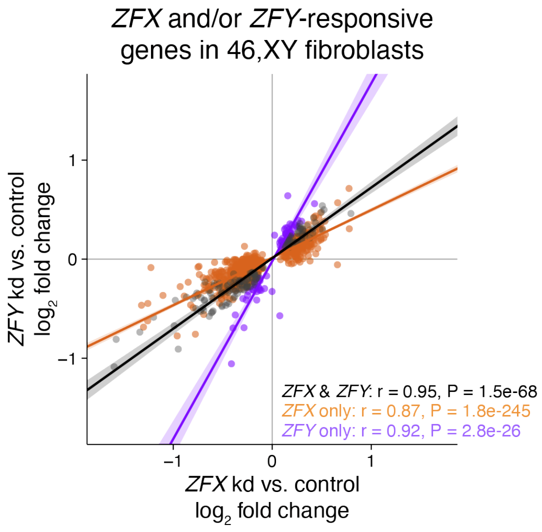


**Figure S19. Correlation plots for significantly *ZFX* and/or *ZFY* responsive genes, related to Figure 6.** Scatterplot showing the relationship between the effects of *ZFX* and *ZFY* knockdown in 46,XY cells for autosomal genes called as significantly responsive ($P_{adj}$<0.05) to either *ZFX* (orange), *ZFY* (purple), or both (grey). Weighted Deming regression lines and 95% confidence intervals (solid purple, orange, or black lines with shading), Pearson correlation coefficients, and Bonferroni-adjusted P values of the correlations are included.

**Figure S20**

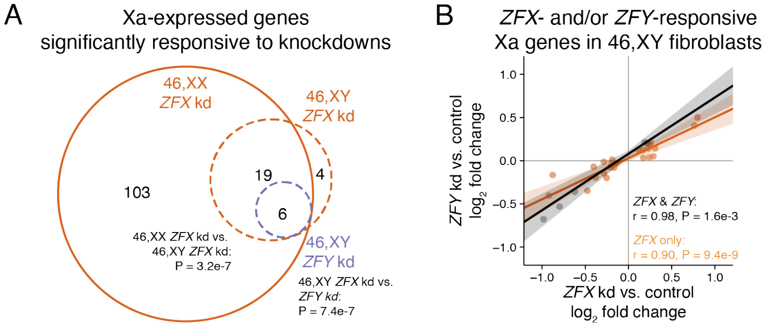


**Figure S20. *ZFY*-responsive Xa-expressed genes are also *ZFX*-responsive, related to Figure 6.** (A) Venn diagram of Xa-expressed genes significantly differentially expressed upon knockdown of *ZFX* in XX or XY cells or upon knockdown of *ZFY* in XY cells. Bonferroni-adjusted P values, hypergeometric test. (B) Scatterplot of Xa-expressed genes in 46,XY cells that were statistically significantly responsive to both *ZFX* and *ZFY* (grey points) or only *ZFX* (orange points), comparing gene-by-gene effects of *ZFX* versus *ZFY* knockdowns. Weighted Deming regression lines and 95% confidence intervals (black or orange lines and shading); Pearson correlation coefficients and Bonferroni-adjusted P values are indicated.

## Figure S21

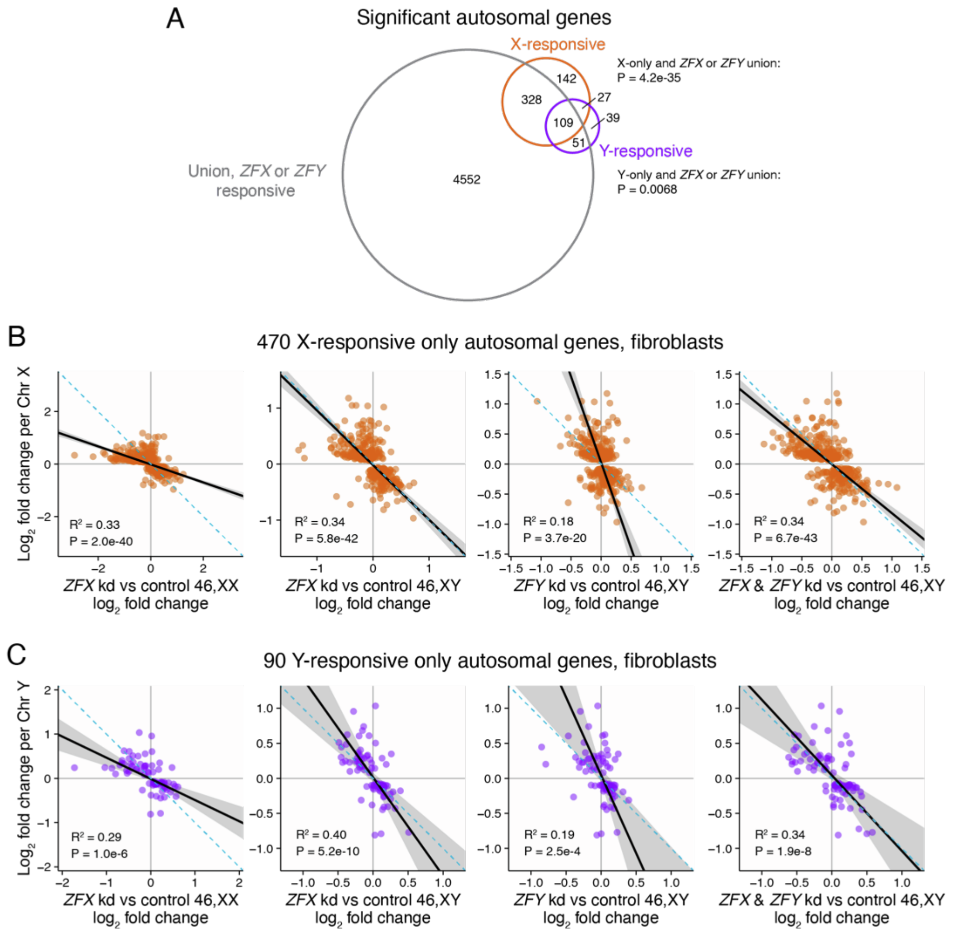


**Figure S21. *ZFX* and *ZFY* response also explains variation in X-responsive-only and Y-responsive-only autosomal genes, related to Figure 7. (A)** Venn diagram of genes significantly responsive to either Chr X or Y copy number in fibroblasts and the union of genes responsive to *ZFX* or *ZFY* across all four CRISPRi experiments. Bonferroni-adjusted P values, hypergeometric test. **(B-C)** Scatterplots for X-responsive-only (B) or Y-responsive-only (C) genes comparing the effect size in response to Chr X (B) or Chr Y (C) copy number with the effect size in response to four *ZFX* or *ZFY* CRISPRi experiments. Black line and grey shading, weighted Deming regression and 95% confidence interval; blue dashed line, identity line (X=Y). Coefficients of determination and Bonferroni-adjusted P values are indicated.

**Figure S22**

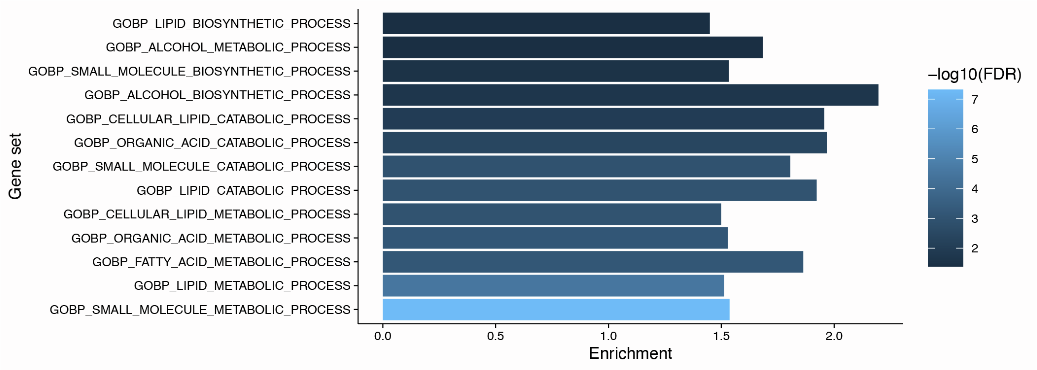


**Figure S22. Functional enrichment in union of significant Chr X or Y responsive autosomal and Xa-expressed genes in LCLs and fibroblasts, related to Figure 1.** Enrichment of "Biological Process" Gene Ontology categories was calculated for the union of autosomal and Xa-expressed genes and genes that significantly responded to Chr X or Y copy number in LCLs and fibroblasts. The union of all expressed autosomal and Xa-expressed genes in LCLs and fibroblasts was used as the background set. Shown here are categories that fall below the Benjamini-Hochberg-corrected P value (FDR) of 0.05, hypergeometric test.

## References

[S1] Rhie, S.K., Yao, L., Luo, Z., Witt, H., Schreiner, S., Guo, Y., Perez, A.A., and Farnham, P.J. (2018). ZFX acts as a transcriptional activator in multiple types of human tumors by binding downstream from transcription start sites at the majority of CpG island promoters. Genome Res. *28*, 310-320. 10.1101/gr.228809.117.

[S2] Ni, W., Perez, A.A., Schreiner, S., Nicolet, C.M., and Farnham, P.J. (2020). Characterization of the ZFX family of transcription factors that bind downstream of the start site of CpG island promoters. Nucleic Acids Res. *48*, 5986-6000. 10.1093/nar/gkaa384.
