# Supplementary material for: The human Y and inactive X chromosomes similarly modulate autosomal gene expression
Source: Cell Genom. 2023 Dec 13;4(1):100462. doi: 10.1016/j.xgen.2023.100462 (PMC10794785; doi:10.1016/j.xgen.2023.100462)
Supplement: Document S2. Article plus supplemental information [file mmc15.pdf]

Cell Genomics

Article

The human Y and inactive X chromosomes similarly modulate autosomal gene expression

Graphical abstract

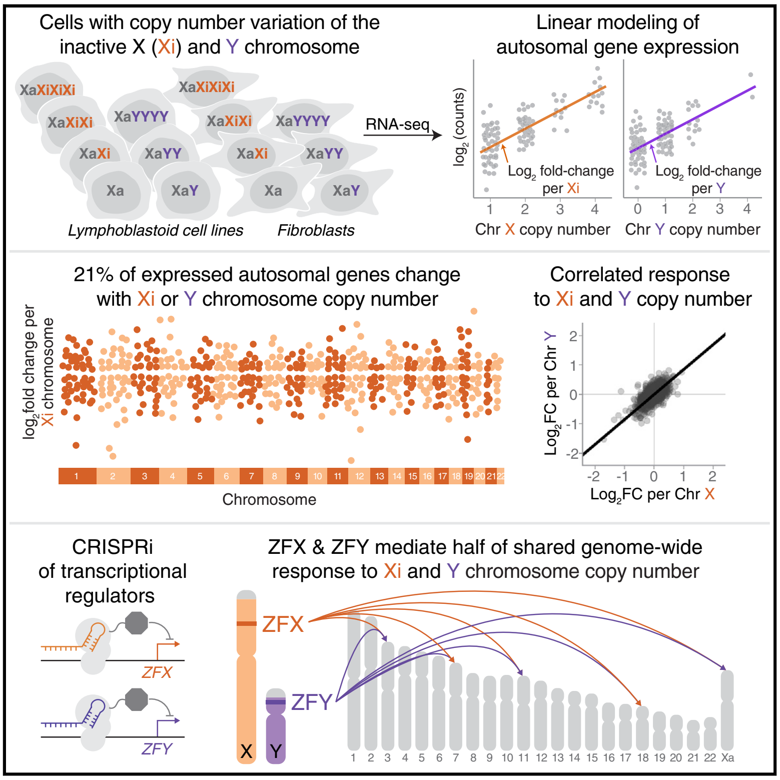

Highlights

- Linear modeling of autosomal gene expression in sex chromosome aneuploidy samples
- Xi and Y affect expression of 21% of all genes expressed in the cell types tested
- Xi and Y have broadly similar effects on gene expression across the genome
- A pair of transcription factors on X and Y mediate much of this shared response

Authors

Adrianna K. San Roman, Helen Skaletsky, Alexander K. Godfrey, ..., Carole Samango-Sprouse, Maximilian Muenke, David C. Page

Correspondence

dcpage@wi.mit.edu

In brief

Using human cells with variation in sex chromosome copy number, San Roman et al. find widespread transcriptomic impacts of the inactive X (Xi) and Y chromosome on autosomes. While Xi elicited larger gene-by-gene effects, Y's effects were largely concordant. They demonstrate that ZFX and ZFY, a pair of conserved transcription factors encoded by the X and Y chromosomes, mediate much of this shared genome-wide transcriptional program.

San Roman et al., 2024, Cell Genomics *4*, 100462
January 10, 2024 © 2023 The Authors.
https://doi.org/10.1016/j.xgen.2023.100462

CellPress

Cell Genomics

CellPress
OPEN ACCESS

Article

The human Y and inactive X chromosomes similarly modulate autosomal gene expression

Adrianna K. San Roman,[1] Helen Skaletsky,[1,3] Alexander K. Godfrey,[1,2] Neha V. Bokil,[1,2] Levi Teitz,[1,2] Isani Singh,[1,4] Laura V. Blanton,[1] Daniel W. Bellott,[1] Tatyana Pyntikova,[1] Julian Lange,[1,2,15] Natalia Koutseva,[1] Jennifer F. Hughes,[1] Laura Brown,[1,3] Sidaly Phou,[1,3] Ashley Buscetta,[5,16] Paul Kruszka,[5,17] Nicole Banks,[5,6] Amalia Dutra,[7] Evgenia Pak,[7] Patricia C. Lasutschinkow,[8] Colleen Keen,[8] Shanlee M. Davis,[9] Angela E. Lin,[10,11] Nicole R. Tartaglia,[9,12] Carole Samango-Sprouse,[8,13,14] Maximilian Muenke,[5,18] and David C. Page[1,2,3,19,*]

[1]Whitehead Institute, Cambridge, MA 02142, USA
[2]Department of Biology, Massachusetts Institute of Technology, Cambridge, MA 02139, USA
[3]Howard Hughes Medical Institute, Whitehead Institute, Cambridge, MA 02142, USA
[4]Harvard Medical School, Boston, MA 02115, USA
[5]Medical Genetics Branch, National Human Genome Research Institute, National Institutes of Health, Bethesda, MD 20892, USA
[6]Eunice Kennedy Shriver National Institute of Child Health and Human Development, National Institutes of Health, Bethesda, MD 20892, USA
[7]Cytogenetics and Microscopy Core, National Human Genome Research Institute, National Institutes of Health, Bethesda, MD 20892, USA
[8]Focus Foundation, Davidsonville, MD 21035, USA
[9]Department of Pediatrics, University of Colorado School of Medicine, Aurora, CO 80045, USA
[10]Medical Genetics, Massachusetts General for Children, Boston, MA 02114, USA
[11]Department of Pediatrics, Harvard Medical School, Boston, MA 02115, USA
[12]Developmental Pediatrics, eXtraOrdinarY Kids Program, Children's Hospital Colorado, Aurora, CO 80011, USA
[13]Department of Pediatrics, George Washington University, Washington, DC 20052, USA
[14]Department of Human and Molecular Genetics, Florida International University, Miami, FL 33199, USA
[15]Present address: Takeda Pharmaceutical Company, Cambridge, MA 02142, USA
[16]Present address: Social and Behavior Research Branch, National Human Genome Research Institute, National Institutes of Health, Bethesda, MD 20892, USA
[17]Present address: GeneDx, Gaithersburg, MD 20877, USA
[18]Present address: American College of Medical Genetics and Genomics, Bethesda, MD 20814, USA
[19]Lead contact
*Correspondence: dcpage@wi.mit.edu
https://doi.org/10.1016/j.xgen.2023.100462

SUMMARY

Somatic cells of human males and females have 45 chromosomes in common, including the "active" X chromosome. In males the 46th chromosome is a Y; in females it is an "inactive" X (Xi). Through linear modeling of autosomal gene expression in cells from individuals with zero to three Xi and zero to four Y chromosomes, we found that Xi and Y impact autosomal expression broadly and with remarkably similar effects. Studying sex chromosome structural anomalies, promoters of Xi- and Y-responsive genes, and CRISPR inhibition, we traced part of this shared effect to homologous transcription factors—*ZFX* and *ZFY*—encoded by Chr X and Y. This demonstrates sex-shared mechanisms by which Xi and Y modulate autosomal expression. Combined with earlier analyses of sex-linked gene expression, our studies show that 21% of all genes expressed in lymphoblastoid cells or fibroblasts change expression significantly in response to Xi or Y chromosomes.

INTRODUCTION

The human X and Y chromosomes evolved from ordinary autosomes over the course of the last 200 million years.[1,2] Today, these homologous chromosomes—typically two X chromosomes in females, and one X and one Y chromosome in males—comprise the oldest, most massive variation in the human genome.

Until recently, this chromosome-scale variation was understood to be of little direct consequence outside the reproductive tract, where the decisive roles of sex chromosome constitution are well established. The reasoning was straightforward: the second X chromosome in 46,XX cells is condensed and transcriptionally attenuated through the process of X chromosome inactivation (XCI),[3,4] and the Y chromosome carries relatively few genes, many of which are not expressed outside the testes.[5,6]

Despite this logic, observations in recent decades have suggested more expansive roles for the second ("inactive") X chromosome in human female cells,[7,8] and even for the Y chromosome in male somatic cells.[9–11] In theory, differing sex chromosome complements in somatic cells could help explain the abundant male-female differences in autosomal gene expression that are seen throughout the human body.[12–14] Still,

This is an open access article under the CC BY license (http://creativecommons.org/licenses/by/4.0/).

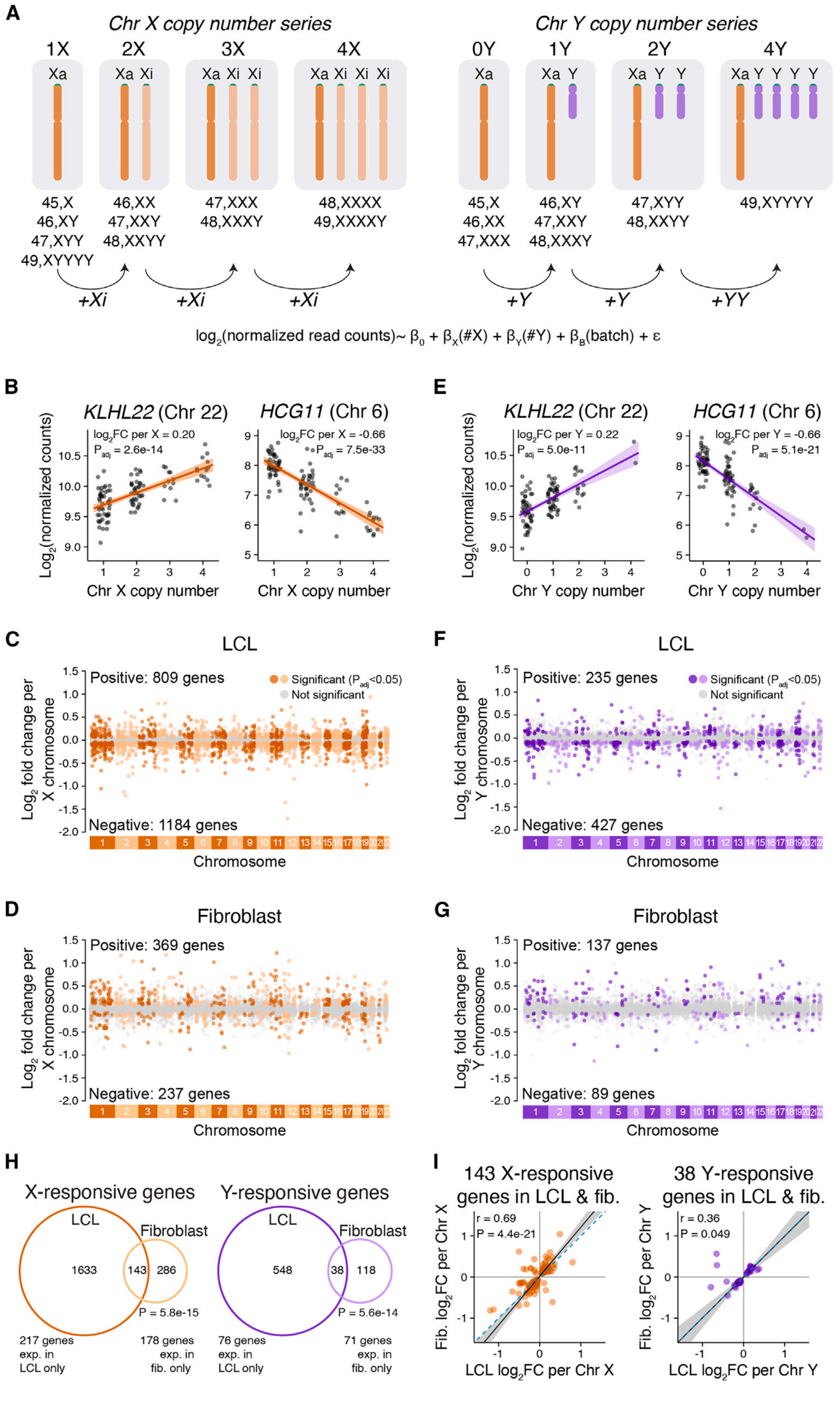

(legend on next page)

it is not known whether these differences in autosomal transcriptomes are driven by sex chromosome constitution, sex steroid hormones, or other biological, behavioral, or environmental factors.

Diploid human somatic cells invariably have one active X chromosome (Xa); most also have an inactive X (Xi) or a Y chromosome, and some have additional Xi or Y chromosomes. We previously modeled expression of X- or Y-linked genes as a function of Xi or Y copy number in cells cultured from individuals with various sex chromosome constitutions: one to four copies of chromosome (Chr) X (zero to three copies of Xi), and zero to four copies of Chr Y.[15] Expression of 38% of X-linked genes changed with additional copies of Xi, and expression of nearly all Y-linked genes increased with additional copies of Chr Y. These Xi- and Y-driven changes were remarkably modular, with successive Xi or Y chromosomes having quantitatively similar effects, facilitating linear modeling. By incorporating allele-specific analyses, we determined that the Chr X gene expression changes are due to a combination of (1) expression from Xi alleles and (2) modulation of Xa transcript levels by Xi, in *trans*.

Given that Xi modulates Xa genes in *trans*, the question arises whether Xi also modulates expression of autosomal genes, potentially via similar mechanisms. A related question is whether Chr Y modulates autosomal gene expression. Based on our recent success in linear modeling of sex chromosome gene expression,[15] we now adapt and expand that modeling to measure how variation in the number of X or Y chromosomes affects autosomal transcription in cultured human cells. We quantified the impact of variation in both Xi and Y chromosome copy number on autosomal transcripts, explored mechanisms responsible for those effects, and discovered that a conserved pair of transcription factors expressed from Xi and Y contribute to modulation of gene expression on autosomes and Xa. An unanticipated finding is that the autosomal impacts of Xi and Y chromosomes are remarkably similar.

RESULTS

Thousands of autosomal genes respond to Chr X and Chr Y copy number

We examined the effects of Chr X and Chr Y copy number on autosomal gene expression using our recently reported RNA-seq dataset from 106 lymphoblastoid cell lines (LCLs) and 99 primary dermal fibroblast cultures.[15] These lines and cultures derive from 176 individuals with one to four X chromosomes and zero to four Y chromosomes (Figure 1A, Table S1). Previously, we reported that each Chr X or Y copy contributes an equal increment of expression for 38% of Chr X genes and nearly all Chr Y genes, which are well-fit with linear models.[15] For each expressed autosomal gene, including protein-coding and long non-coding RNA genes with at least one transcript per million in either 46,XX or 46,XY samples, we fit a linear regression model in DESeq2[16] to estimate the $\log_2$ fold change in expression per copy of Chr X or Y (STAR Methods).

This analysis revealed that Chr X and Chr Y copy number had widespread effects on autosomal expression. The magnitudes of individual gene expression changes per Chr X or Y were modest—with most changes less than 1.5-fold—but many genes were affected. There were 1,993 significantly (adjusted p value [P_{adj}]<0.05) X-responsive genes in LCLs—comprising 18% of expressed autosomal genes—and 606 in fibroblasts—5% of expressed autosomal genes (Figures 1B–1D; full results in Table S2). Chr Y copy number had a statistically significant effect on substantially fewer autosomal genes: 662 Chr Y-responsive genes in LCLs—6% of expressed autosomal genes—and 226 in fibroblasts—1.9% of expressed autosomal genes (Figures 1E–1G, Table S2). Gene ontology analyses of the Chr X- or Y-responsive genes yielded few significantly enriched functional categories (STAR Methods, Figure S1). We conclude that variation in Chr X and Y copy numbers significantly and broadly alters autosomal gene expression.

We considered and tested various interpretations of these findings. First, we asked whether X- or Y-responsive autosomal genes were statistical artifacts of enhanced sex-chromosomal expression altering the genome-wide distribution of sampled read counts. To this end, we renormalized the data and refit the same linear models using only autosomal genes, obtaining virtually identical results (STAR Methods, Figure S2).

Second, we tested whether there was any impact of modeling gene expression as a function of both variables—Chr X copy number and Chr Y copy number—together compared with models in which we used subsets of samples to vary either Chr X or Chr Y copy number individually. We found that the full model was highly correlated genome-wide with models of the individual

Figure 1. Genome-wide response to Chr X or Y copy number is distinct in two cell types

(A) RNA-seq data from LCLs and fibroblasts spanning a range of Chr X and Y copy numbers were analyzed using linear regression, modeling $\log_2$ expression as a function of Chr X copy number, Chr Y copy number, and batch. Number of samples per karyotype in LCLs: 17 45,X; 22 46,XX; 17 46,XY; 7 47,XXX; 11 47,XXY; 10 47,XYY; 1 48,XXXX; 4 48,XXXY; 3 48,XXYY; 12 49,XXXXY; 2 49,XYYYY. Number of samples per karyotype in fibroblasts: 23 45,X; 20 46,XX; 14 46,XY; 4 47,XXX; 30 47,XXY; 5 47,XYY; 1 48,XXXY; 1 49,XXXXY; 1 49,XYYYY.

(B and E) Examples of individual autosomal genes that significantly respond to Chr X (B) or Chr Y (E) copy number in LCLs. Each point represents the expression level in an individual sample with the indicated number of X or Y chromosomes. The regression lines and confidence intervals, $\log_2$ fold change per copy of Chr X or Y, and adjusted p values (P_{adj}) from linear regressions are indicated.

(C, D, F, G) Log_2 fold change per copy of Chr X or Y for 11,034 expressed autosomal genes in LCLs and 12,002 expressed autosomal genes in fibroblasts, plotted by chromosomal location. Genes in gray do not significantly change in response to X or Y copy number, while genes in dark or light colors (odd- or even-numbered chromosomes, respectively) represent significantly Chr X- (C, D) or Chr Y-responsive (F, G) genes.

(H) Venn diagrams showing the overlap of genes that significantly respond to X or Y copy number ($P_{adj} < 0.05$) in LCLs and fibroblasts. Genes expressed in both cell types were included in the Venn diagrams, and genes with cell-type-specific expression are noted below. Bonferroni-adjusted p values, hypergeometric test.

(I) Scatterplots of genes that are Chr X- or Y-responsive in both LCLs and fibroblasts show mostly similar $\log_2$ fold change ($\log_2$FC) values across cell types. Black line and gray shading, weighted Deming regression and 95% confidence interval; blue dashed line, identity (X = Y) line. Pearson correlation coefficients and Bonferroni-adjusted p values are indicated.

contributions of Chr X and Y copy number (Figure S3). Moreover, by conducting the Chr X analysis in samples with and without Y chromosomes, we found that the autosomal effects of Xi copy number are correlated in cells derived from anatomically male and female individuals (Figure S3).

Third, we tested the impact of sample size on the number of significantly Chr X- or Y-responsive genes detected in our analysis to ask whether we have saturated the analysis with our current sample set. Using a bootstrapping analysis (STAR Methods), we found that sequencing cell lines from additional individuals would lead to discovery of more sex chromosome-responsive genes, but they would likely have small effect sizes (Figure S4).

Finally, because individuals across our dataset have differing hormonal profiles that might be correlated with Chr X or Y copy number, we evaluated whether a memory of differential hormonal exposures in the body prior to tissue sampling might explain the results. First, we examined the expression levels of estrogen receptors alpha (*ESR1*) and beta (*ESR2*) and the androgen receptor (*AR*), finding low levels of expression in both LCLs and fibroblasts; only *ESR2* in LCLs and *AR* in fibroblasts exceeded the 1 transcript per million (TPM) minimum expression cutoff, with median TPM of 1.5 and 2.3, respectively, across samples. Next, we compared autosomal genes that significantly respond to Chr X or Y copy number with direct target genes for estrogen or androgen receptors. Most X- or Y-responsive genes did not overlap with estrogen or androgen receptor direct target genes (STAR Methods, Table S3, Figure S5).[17,18] There was a modest enrichment of androgen receptor targets among Y-responsive genes in fibroblasts, but their effects showed no evidence of correlation (Spearman $r = 0.034$, $p = 0.9$; Figure S5D). Thus, we did not find evidence that sex hormones explain the observed impacts of Chr X or Y copy number. In sum, thousands of autosomal genes respond significantly to Chr X or Y copy number, with more genes responding to X than to Y, and more in LCLs than in fibroblasts.

Response to Chr X or Chr Y copy number is mostly cell type specific

We investigated whether individual autosomal genes responded to Chr X or Chr Y copy number in LCLs only, in fibroblasts only, or in both cell types. The majority of significantly responsive autosomal genes were responsive in only one of the two cell types; some of these genes were "cell-type-specific"—that is, they are only expressed in only one of the two cell types—and others were expressed in both cell types, but only responsive in one. Among the significantly Chr X- or Y-responsive genes in LCLs, 93% and 94%, respectively, were not significantly responsive in fibroblasts; among significantly Chr X- or Y-responsive genes in fibroblasts, 76% and 83% were not significantly responsive in LCLs (Figure 1H). Indeed, across autosomal genes expressed in both cell types, we observed only a weak correlation between responses to Chr X and to Chr Y (Figure S6). While most significantly responsive genes were responsive in only one of the two cell types, the overlap was greater than expected by chance (Figure 1H), and most of these overlapping genes responded with the same directionality—rising (or falling) in response to Chr X or Y copy number in both LCLs and fibroblasts (Figure 1I). Thus, a minority of the genome-wide response to sex chromosome copy number is shared between the two cell types; the majority is cell-type-specific.

Chr Xi and Y elicit similar responses across autosomes and Xa

Next, we compared, within each cell type, the autosomal responses to Chr X and to Chr Y. Among autosomal genes with statistically significant responses to Chr X or Y copy number, we found that 22% of X-responsive genes and >60% of Y-responsive genes were shared—442 genes in LCLs and 136 in fibroblasts (Figure 2A). These co-regulated genes' responses to X and Y were highly concordant in polarity and magnitude (Pearson correlation in LCLs: 0.95 and fibroblasts: 0.96), suggesting that the mechanisms governing these responses might be shared (Figure 2B; see example genes in Figures 1B and 1E). Given the remarkable correlation of responses to Chr X and Y copy number for these shared genes, we further investigated the gene-by-gene effects of Chr X and Y on the "Chr X-responsive-only" autosomal genes. Interestingly, we observed a strong correlation of Chr X and Y responses for these genes (LCLs: 0.79, fibroblasts: 0.88; Figure S7). This highly correlated response to Chr X and Y could even be observed when assessing all expressed genes (LCLs: 0.67, fibroblasts: 0.6), with significantly larger effects in response to Chr X compared with Chr Y (Figure 2C). These results indicate that additional copies of Chr Xi and Y modulate a similar group of genes, with Xi exerting larger gene-by-gene effects than Y.

In a previous study, we showed that Xi copy number modulates gene expression from Xa in trans.[15] In light of our current findings, we wondered whether Chr X and Chr Y copy number might have shared effects on Xa gene expression. We first conducted a new meta-analysis, compiling data from studies quantifying Xi expression to identify strictly "Xa-expressed" genes that are consistently "silenced" on Xi across studies (STAR Methods; Table S4).[7,8,15,19–22] Using these annotations, we found that slightly larger proportions of Xa-expressed genes were impacted by Chr X or Y copy number than on the autosomes: 57 (20%) and 27 (8.6%) Xa-expressed genes were significantly responsive to Chr X copy number in LCLs and fibroblasts, respectively, and 23 (8.2%) and 10 (3.2%) were responsive to Chr Y copy number (Table S4). Among these, 12 genes in LCLs and seven genes in fibroblasts significantly responded to both Chr X and Y copy numbers (Figure 2D). Like the autosomal genes, the responses of Xa-expressed genes to Chr X and Chr Y copy number were highly concordant in magnitude and polarity (Figures 2E and S7B). This suggests that Xi and Y modulate expression of a defined subset of genes on Xa in *trans* through shared mechanisms.

To identify the mechanisms driving the genome-wide shared response to Chr X and Chr Y copy numbers, we considered and tested three hypotheses. First, we asked whether the observed expression changes reflect a generic response to aneuploidy that is not specific to the sex chromosomes (and has been reported for autosomes[23]). Second, we tested whether a "heterochromatin sink" created by additional sex chromosomes pulls heterochromatin factors away from and thereby de-represses (activates) autosomal genes. Third, we investigated the actions

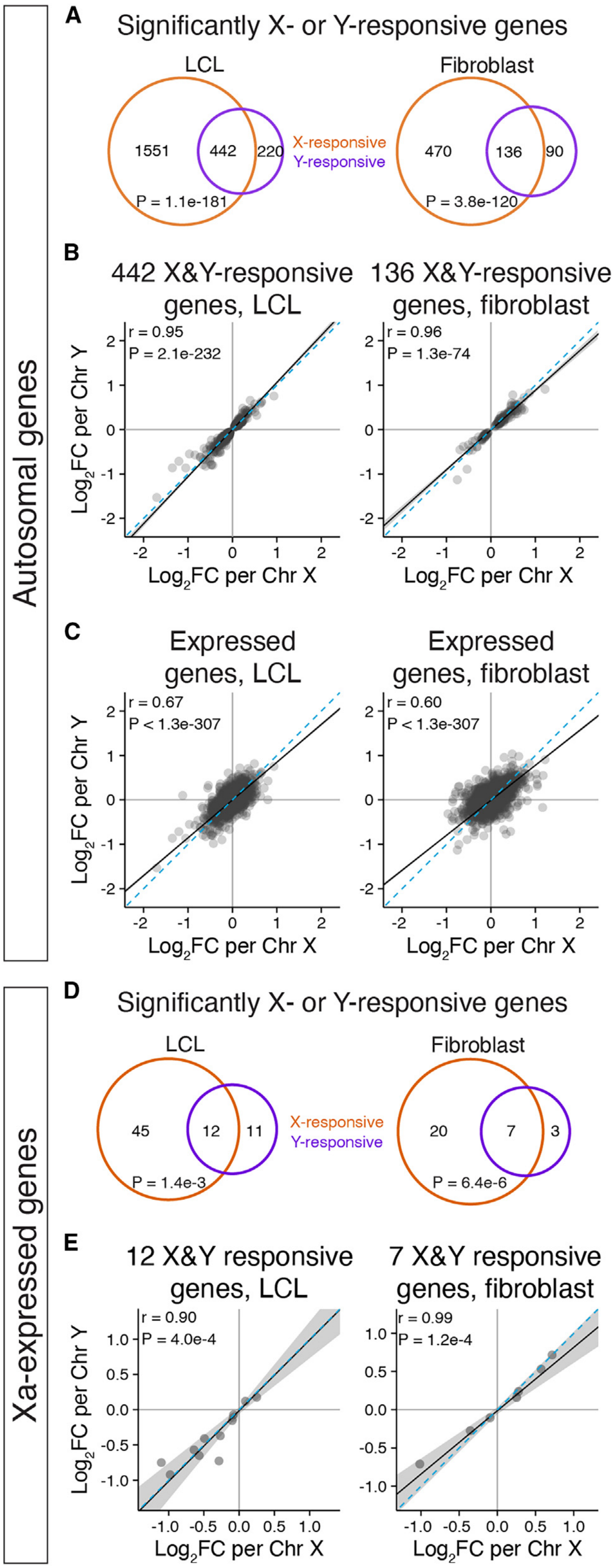

Figure 2. Chr X and Y copy number have similar genome-wide effects

(A and D) Venn diagrams of significantly ($P_{adj} < 0.05$) X- and Y-responsive autosomal (A) or Xa-expressed (D) genes reveal significant overlap. Bonferroni-adjusted p values, hypergeometric test.

(B and E) Scatterplots of $\log_2$ fold change ($\log_2$FC) per Chr X vs. Chr Y for significantly Chr X- and Y-responsive autosomal (B) or Xa-expressed (E) genes show correlated responses.

(C) Scatterplots of all expressed autosomal genes in LCLs and fibroblasts show globally correlated response to Chr X and Chr Y copy number. In (B), (C), and (E): black line and gray shading depict weighted Deming regression and 95% confidence interval; blue dashed line, identity (X = Y) line; Pearson correlation coefficients and Bonferroni-adjusted p values are indicated.

of regulatory genes shared between the X and Y chromosomes. We will consider each of these hypotheses in turn.

The shared response to Chr X and Chr Y copy number is not a generic response to aneuploidy

To test the first hypothesis, we analyzed RNA-seq data from LCLs with three copies of Chr 21 (47,XX+21 and 47,XY+21) and compared these with our data from 46,XX and 46,XY LCLs (Figure 3A, Table S1).[15] We employed linear modeling to estimate the $\log_2$ fold change in expression per additional Chr 21, controlling for sex chromosome constitution (46,XX or 46,XY) (STAR Methods). Chr 21 had large effects across the genome: in addition to expression increases for Chr 21 genes (previously reported in San Roman et al.[15]), we identified 980 genes on other chromosomes (including X and Y) that were significantly Chr 21-responsive (Figures 3B–3D, Table S5).

We then compared the autosomal responses to Chr 21 copy number with responses to Chr X or Chr Y copy number. Across the autosomes, Chr 21-responsive genes displayed significantly larger fold changes compared with Chr X- or Y-responsive genes (Figures 3E–3G). Importantly, most X- or Y-responsive autosomal genes did not overlap with the Chr 21-responsive gene set (Figure 3H). There was a modest overlap between Chr 21-responsive and Chr X-responsive genes, but the effects of Chr 21 and Chr X were weakly correlated (Figure 3I). Moreover, none of the Xa-expressed genes that responded to both Chr X and Y copy number in LCLs were among the Chr 21-responsive Chr X genes (Figure S8). We conclude that features unique to the sex chromosomes, rather than a generic aneuploidy response, explain autosomal (and Xa) gene expression changes in response to Chr X and Y copy number.

The response to Chr Y copy number is not due to heterochromatin sinks

"Heterochromatin sink" effects are well documented in *Drosophila*, where Y chromosomes with varying amounts of heterochromatin affect expression of autosomal genes adjacent to heterochromatic regions—a phenomenon known as position-effect variegation.[24] The human Chr Y has a large heterochromatic region on its long arm (Yq); in theory, the increase in heterochromatin with additional copies of Chr Y could pull heterochromatin factors away from autosomal genes, thereby increasing their expression. We studied two kinds of human variation to test whether Chr Y heterochromatin impacts autosomal gene

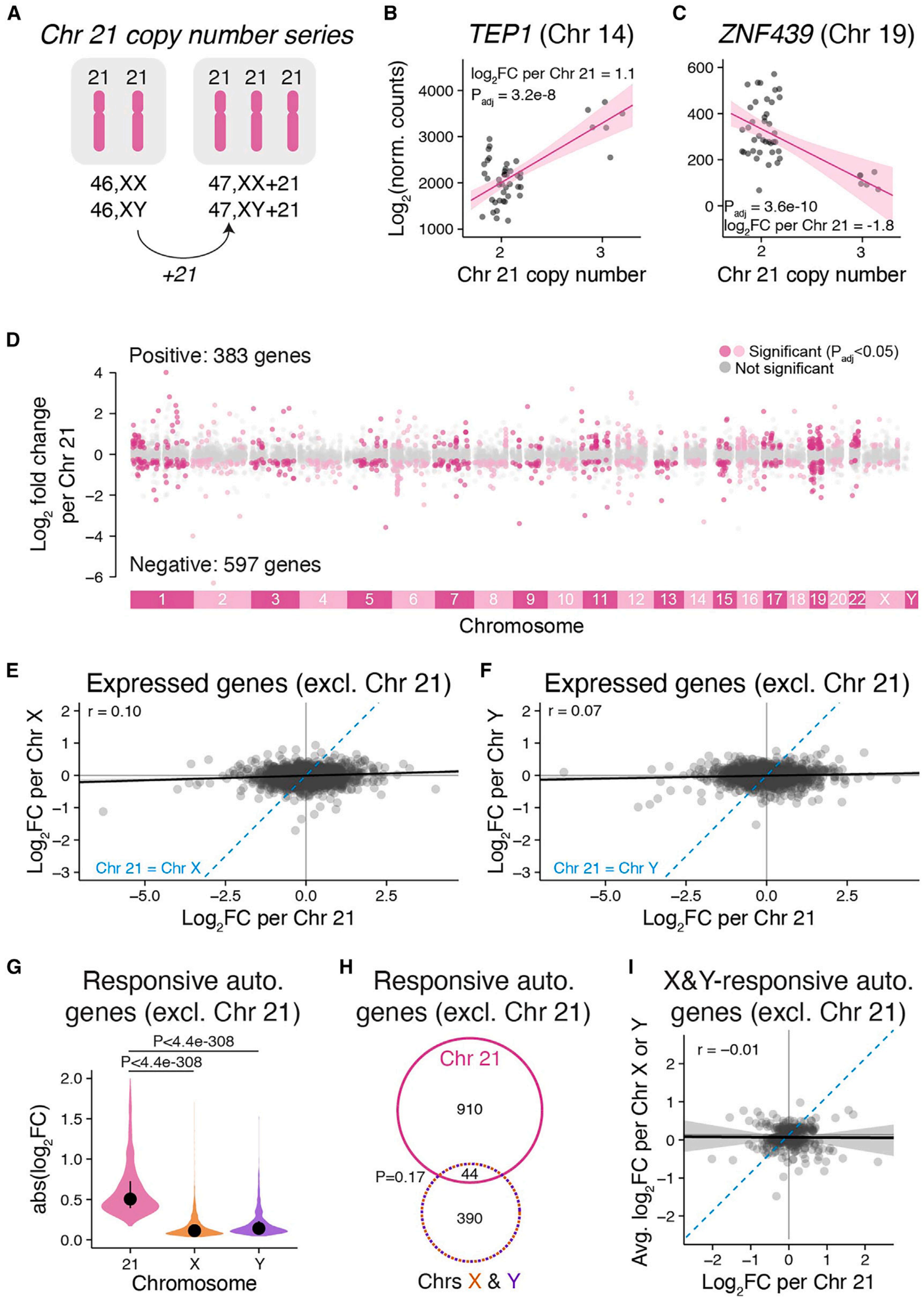

Figure 3. The shared response to Chr X and Y copy number is not a general response to aneuploidy

(A) RNA-seq data from LCLs with two or three copies of Chr 21 were analyzed using linear regression.

(B and C) Scatterplots and regression lines with confidence intervals of individual autosomal genes showing expression changes in samples with two or three copies of Chr 21. The $\log_2$ fold change per Chr 21 and P_{adj} from linear regressions are indicated.

(legend continued on next page)

expression: (1) common polymorphisms in Yq heterochromatin length,[25] and (2) rare structurally variant Y chromosomes.

To quantify Yq heterochromatin length, we mapped whole-genome sequencing data from 1,225 male samples from the 1000 Genomes Project[26] to *DYZ1*, a repetitive sequence specific to the Yq heterochromatic region.[6] The average depth of coverage of the *DYZ1* sequence, which reflects the number of repeats and size of the Yq heterochromatic region, ranged from 23.8 to 896.5 reads (after normalization to depth of coverage of a single-copy region of the Y chromosome), with a median of 202.5 (Figure S9; Table S6). For 194 samples for which RNA-seq data were also available,[27] we modeled gene expression as a function of heterochromatin length as approximated by *DYZ1* read depth, finding only three significantly differentially expressed genes (Figures 4A and 4B). We conclude that variation in Yq heterochromatin length does not affect genome-wide expression.

We next analyzed RNA-seq data from individuals with an intact X chromosome and a structurally variant Y "isochromosome" in which Yq heterochromatin is deleted and a portion of the chromosome, including widely expressed Chr Y genes on the short arm (Yp), is duplicated in "mirror image" orientation, resulting in higher Chr Y gene expression (Figures 4C, S10, and Table S1). If Yq heterochromatin contributes significantly to expression of shared X- and Y-responsive autosomal genes, we would expect cells missing this region to show expression like that of 45,X cells, which also lack Yq heterochromatin. Using principal-component analysis of the 442 autosomal genes responsive to both Chr X and Y copy number in LCLs, we found that samples with variant Y chromosomes did not cluster with 45,X samples but were instead more similar to 47,XYY samples (Figure 4D). Together, these experiments indicate that a heterochromatin sink does not explain the response of autosomal genes to Chr Y copy number, and therefore cannot explain the shared response to Chr X and Chr Y copy number.

NPX-NPY gene pairs drive shared autosomal response to X and Y copy number

Two groups of genes common to Chr X and Y could drive the shared autosomal response (Figure 5A). First are genes in the pseudoautosomal region (PAR), a region of identity on the distal short arms of Chr X and Y; expression of these genes increases linearly with sex chromosome copy number.[15] Second are homologous gene pairs in the non-pseudoautosomal regions of Chr X (NPX) and Y (NPY), which increase in expression with X or Y copy number, respectively. These NPX-NPY gene pairs are remnants of the ~200-million-year evolutionary process that differentiated a pair of ordinary autosomes into Chr X, which retains 98% of the ancestral gene content, and Chr Y, which retains only 3%. The NPX-NPY pairs are involved in important cellular processes, including transcription, epigenetic regulation, and translation,[10] and have diverged in sequence to varying degrees,[6] possibly maintaining identical functions. Of the NPX-NPY pair genes, four are of particular interest because they encode proteins that affect genome-wide expression: the histone lysine demethylases *KDM6A*/*UTY* and *KDM5C*/*KDM5D*, RNA helicases *DDX3X*/*DDX3Y*, and transcription factors *ZFX*/*ZFY*. Since PAR and NPX-NPY pair gene copy numbers are usually correlated, we decoupled their effects using cell lines from individuals with structurally variant X or Y chromosomes in which PAR or NPX-NPY genes are deleted or duplicated.

We performed RNA-seq on four individuals who have, in addition to one structurally normal X chromosome, an X isochromosome in which some or all of the short arm (Xp) is deleted and the long arm (Xq) is duplicated in "mirror image" orientation (Table S1). Three different isochromosome structures with breakpoints in distal Xp (iXq-dist), or in proximal Xp (iXq-prox), or at the centromere (iXq-cen) were identified by fluorescent *in situ* hybridization (FISH) and confirmed by RNA-seq read counts (Figures S11–S13). The X isochromosomes are all missing PAR1, but they differ with respect to copy numbers of the four NPX-NPY genes of interest: iXq-dist retains them, iXq-prox is missing three (*ZFX*, *DDX3X*, and *KDM6A*), and iXq-cen is missing all four (Figures 5B and 5C; see Table S7 for copy numbers of PAR1 and NPX-NPY pair genes in cell lines with structural variants).

We also assessed gene expression in cells from individuals with one normal Chr X and an X-Y translocation product arising from recombination between highly similar genes in NPX and NPY, either *PRKX* and *PRKY* or *ANOS1 (KAL1)* and *ANOS2P (KALP)* (Figure 5D).[28] We identified these translocations using a PCR-based screening strategy, and we confirmed that RNA-seq read counts were consistent with the identified chromosomal breakpoints (Figures S12, S14, S15, and Table S7). Three individuals had *PRKX-PRKY* translocated chromosomes that retained PAR1 but lacked distal Yp, resulting in loss of *ZFY*. Four individuals had *ANOS1-ANOS2P* translocated chromosomes that lacked PAR1 but retained most NPX-NPY gene pairs (Table S7).

To determine if the shared autosomal response to X and Y copy number is due to PAR1 or NPX-NPY genes, we compared gene expression in the cell lines with structurally variant sex chromosomes to those with sex chromosome aneuploidy, using principal-component analysis of the shared Chr X- and Y-responsive autosomal genes in LCLs (442 genes) or fibroblasts (136 genes). If PAR1 genes drive the X-Y-shared autosomal response, we would expect all 46,X,i(Xq) and 46,X,t(X;

(D) The $\log_2$ fold changes per Chr 21 across all chromosomes excluding (excl.) Chr 21 are plotted by chromosomal location. Genes in gray are expressed, but do not significantly change in response to Chr 21 copy number, while genes in dark or light pink (colored by every other chromosome for clarity) represent significantly Chr 21-responsive genes.

(E and F) Scatterplot of all expressed autosomal genes, excluding Chr 21, comparing Chr 21 response to Chr X (E) or Chr Y (F) response. Blue dashed line, Chr 21 = Chr X (E) or Chr Y (F).

(G) Violin plot with median and interquartile range of the absolute values of the $\log_2$ fold changes for significantly Chr 21, X, or Y-responsive autosomal genes excluding Chr 21. Numbers of genes included in the plot: 1,030 for Chr 21, 1,968 for Chr X, and 653 for Chr Y. Bonferroni-adjusted p values, Wilcoxon rank-sum test.

(H) Venn diagram of Chr X- and Y- and Chr 21-responsive genes shows little overlap (p values, hypergeometric test).

(I) Scatterplot of autosomal genes (excluding Chr 21) regulated by Chr X and Y shows little correlation with responses to Chr 21. All scatterplots: black line and gray shading, Deming regression and 95% confidence interval; blue dashed line, identity (X = Y) line; Pearson correlation coefficients are indicated.

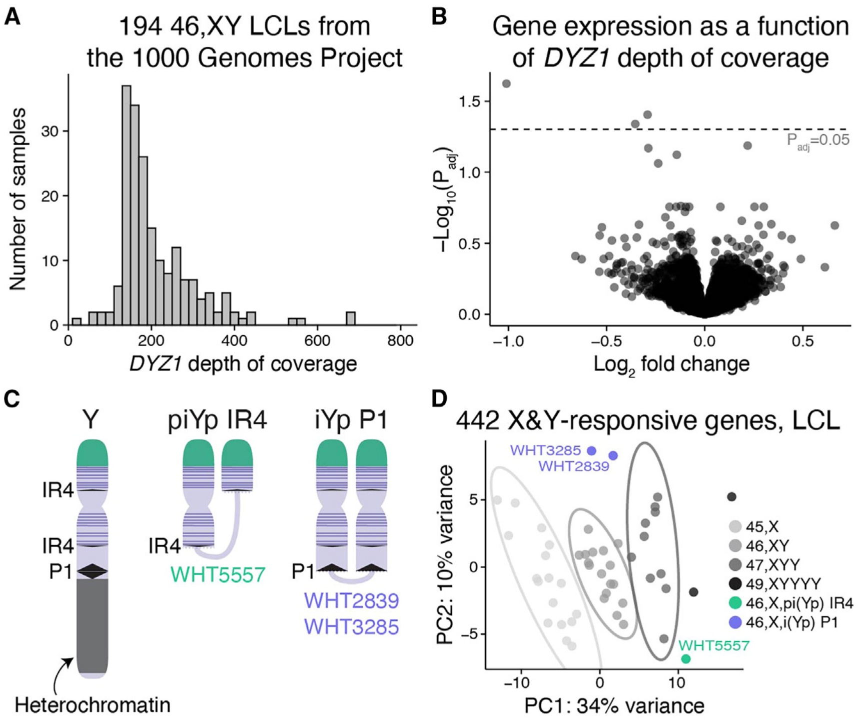

Figure 4. Heterochromatin sink model does not explain genes upregulated in response to Chr Y copy number

(A) Histogram of *DYZ1* depth of coverage for the 194 46,XY samples from the 1000 Genomes Project that have paired RNA-sequencing data.

(B) Each point represents the magnitude and significance of expression change in response to *DYZ1* depth of coverage for expressed autosomal genes in LCLs. Only three genes are below the significance cutoff of $P_{adj} = 0.05$.

(C) Schematic of the normal Y chromosome and two types of variant Y chromosomes that have recombined in repeated DNA regions to generate chromosomes missing the large heterochromatic region on the long arm and have two copies of many or all Chr Y genes expressed in somatic cells (purple horizontal lines). One Y pseudoisochromosome (piYp) resulted from recombination in an inverted repeat (IR4) on Yp and Yq, and a different Y isochromosome (iYp) resulted from recombination in a palindrome (P1) on Yq.

(D) Principal-component analysis (PCA) plot showing separation of samples based on expression of the 442 autosomal genes that are X- and Y-responsive in LCLs. Ellipses represent 95% confidence intervals around the centroid of each karyotype group with at least three samples. The 46,X,i(Yp) and 46,X,pi(Yp) samples cluster away from 45,X samples and near those with two Y chromosomes (47,XYY).

Y)(*ANOS1-ANOS2P*) samples—which retain one copy of PAR1—to cluster with 45,X samples, and 46,X,t(X; Y)(*PRKX-PRKY*) samples—which retain two copies of PAR1—to cluster with 46,XX and 46,XY samples. Instead, we found that the clustering of 46,X,i(Xq) samples depended on the copy number of NPX genes on Xp. For example, the 46,X,i(Xq-cen) and 46,X,i(Xq-prox) samples—with one copy of most or all NPX-NPY pair genes on Xp—clustered with the 45,X samples, while the 46,X,i(Xq-dist) sample—with three copies of most NPX-NPY pairs—clustered with the 46,XX samples (one might expect this sample to cluster with 47,XXX samples, but, due to mosaicism for a 45,X cell line [STAR Methods], expression averages to 46,XX levels; Figures 5E and 5F). Similarly, 46,X,t(X; Y)(*ANOS1-ANOS2P*) samples—with two copies of most NPX-NPY pairs—clustered with 46,XX samples, while 46,X,t(X; Y)(*PRKX-PRKY*) samples—with reduced copy number of only two NPX-NPY pairs: *ZFX*/*ZFY* and *RPS4X*/*RPS4Y*—clustered with 45,X samples (Figure 5G). In all, these results indicate that NPX-NPY pairs, not PAR1 genes, drive the shared autosomal response to X and Y copy number in LCLs and fibroblasts. Among NPX-NPY pairs, only one pair maps to Xp and has one less copy in 46,X,t(X; Y)(*PRKX-PRKY*) samples: *ZFX* and *ZFY*.

ZFX and *ZFY* activate a shared transcriptional program genome-wide

To determine whether *ZFX* and *ZFY* mediate the genome-wide response to X or Y copy number, we searched for DNA motifs in promoters of X- or Y-responsive genes (STAR Methods). Motifs matching previously characterized ZFX DNA-binding signatures were enriched in genes that were positively X- or Y-responsive but not among genes that were negatively X- or Y-responsive; the highest enrichment was among genes positively responsive to *both* X and Y in LCLs (Table S8). The motif database we queried does not include a ZFY motif, but *ZFX* and *ZFY* are 93% identical in both DNA and predicted amino acid sequence,[6] and evidence indicates that ZFY occupies the same genomic sites as ZFX: their zinc-finger domains and genome-wide binding profiles are nearly identical.[29–31]

Next, we assessed functional evidence that ZFX, and possibly ZFY, act at X- or Y-responsive genes. Reanalysis of ENCODE data revealed that ZFX protein binding is enriched at promoters of genes positively responsive to X and Y in LCLs and fibroblasts (Figure S16, Table S9, STAR Methods). Moreover, published target genes activated by ZFX in three cell lines (MCF-7 breast cancer cells, C4-2B prostate cancer cells, and human embryonic kidney [HEK 293T] cells) were significantly enriched among genes positively, but not negatively, responsive to X and/or Y dosage in LCLs and fibroblasts (Figure S17; Table S10, STAR Methods).[31,32] This aligns well with studies showing that ZFX is a transcriptional activator[32–35] and strengthens previous evidence of ZFX motif enrichment in genes positively regulated by Chr X copy number identified using microarray data.[36,37] Since positively Y-responsive genes were enriched for ZFX targets, we hypothesized that ZFX's gene-activating functions were retained by ZFY during Y chromosome evolution.

To directly assess the functions of ZFX and ZFY genome-wide, we knocked down *ZFX* in 46,XX fibroblasts (from three individuals) and *ZFX* and/or *ZFY* in 46,XY fibroblasts (from three individuals) using CRISPR interference (CRISPRi[38]; Figure 6A, STAR Methods), followed by RNA-sequencing. To identify differentially

CellPress
OPEN ACCESS

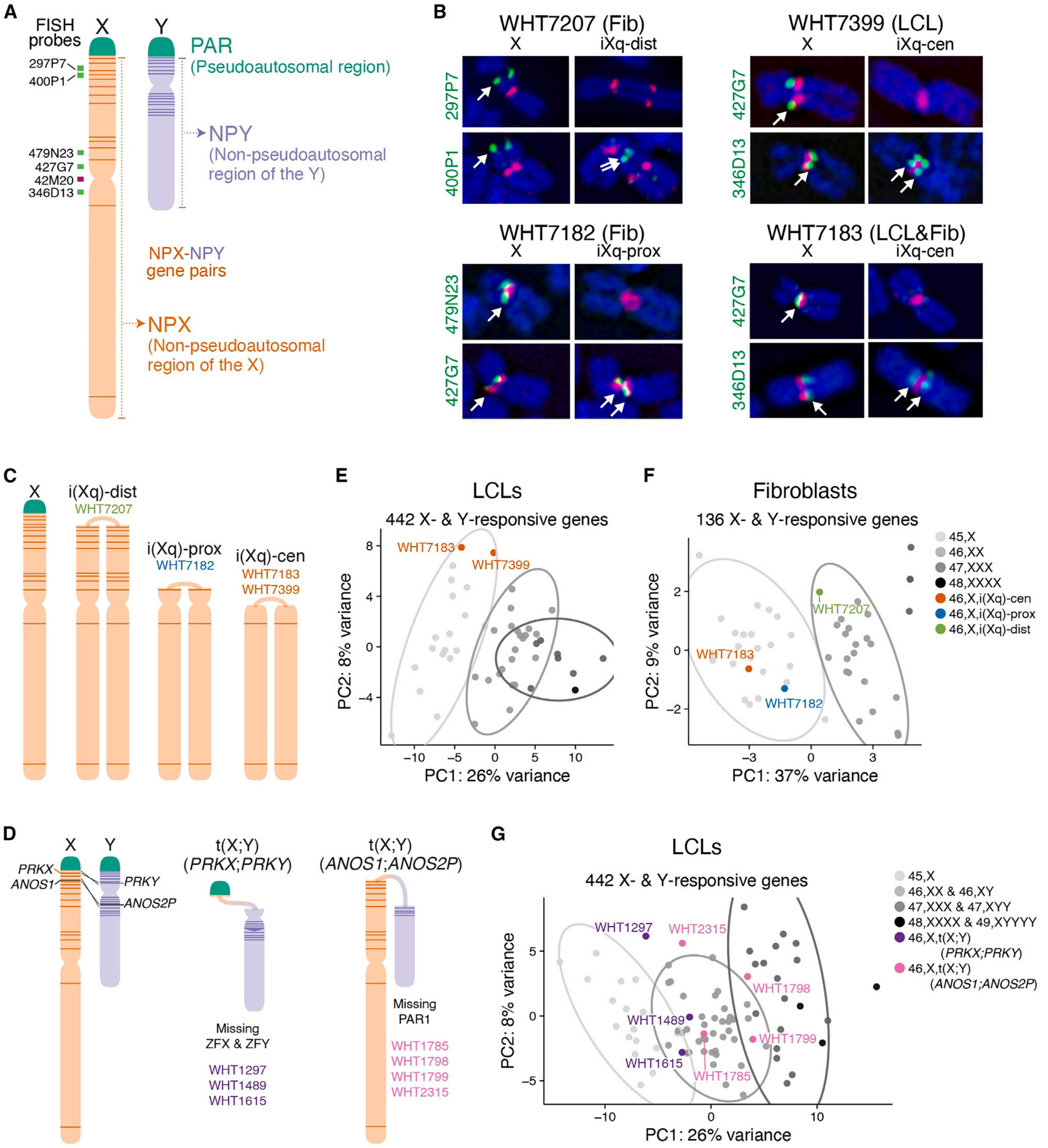

Figure 5. NPX-NPY pair genes on Xp (the short arm), but not PAR1 genes, drive the shared genome-wide response to X and Y chromosome copy number

(A) Anatomy of the sex chromosomes. Locations of FISH probes used in (B) are indicated.

(B) DNA FISH on four 46,X,i(Xq) samples refined the sites of recombination. Fluorescent images of the normal X and iXq from the same cell are shown side-by-side, with probes across the chromosome (green) and the X centromere (red). Full FISH results are found in Figure S11.

(C) Schematic of a normal Chr X and three types of X isochromosomes. Locations of NPX-NPY pair genes indicated by dark orange lines. The sample IDs for individuals carrying each type of X isochromosome are provided. We obtained matching LCLs and fibroblasts for one WHT7183, while we only had LCLs from WHT7399 and only fibroblasts for WHT7182 and WHT7207.

(legend continued on next page)

expressed genes, we modeled gene expression as a function of the CRISPRi target gene (*ZFX* and/or *ZFY* versus control; STAR Methods). These CRISPRi knockdowns were effective: in single knockdowns we observed an ~80% reduction of *ZFX* and >90% reduction of *ZFY* levels (Figure 6B). The knockdowns were specific: *ZFX* expression was not affected by *ZFY* knockdown, and vice versa, which suggests that ZFX and ZFY are not strong transcriptional regulators of each other. In a separate experiment, double knockdowns were performed resulting in more modest but significant reductions of both *ZFX* (~40%) and *ZFY* (~55%).

The breadth of the genome-wide effects varied among the knockdowns: in 46,XX cells, 4,638 genes were differentially expressed upon *ZFX* knockdown, while in 46,XY cells, 943 genes were differentially expressed upon *ZFX* knockdown, 206 genes upon *ZFY* knockdown, and 1,698 genes upon *ZFX/ZFY* double knockdown; in most cases, gene expression levels changed less than 2-fold (Figures 6C–6F, Table S11). The knockdowns confirm that ZFX and ZFY are transcriptional activators: genes that decrease in expression with *ZFX* and/or *ZFY* knockdown in 46,XX and 46,XY cells were enriched for ZFX DNA-binding motifs and protein binding at their promoters, compared with genes that were not significantly affected; conversely, genes that increased in expression were depleted for ZFX binding (Figure S18, Table S12).

Several lines of evidence indicate that *ZFX* and *ZFY* act in a mutually and cumulatively dose-dependent fashion. First, the magnitudes of the genome-wide effects in 46,XX and 46,XY cells were inversely related to the cumulative level of *ZFX+ZFY* expression remaining after knockdown (Figure 6G). Second, although there were fewer genes that significantly responded to *ZFY* knockdown compared with *ZFX* knockdown in 46,XY cells, 135 (69% of) *ZFY*-responsive genes also responded to *ZFX* knockdown, and these responses were nearly perfectly correlated, with larger responses to *ZFX* than to *ZFY* knockdown ($p = 7.2 \times 10^{-8}$, paired t test; Figures 6H and 6I). Third, even among genes that were significantly responsive to either *ZFX* or *ZFY* knockdown (*ZFX* "only" or *ZFY* "only"), gene-by-gene responses to *ZFX* and *ZFY* knockdowns were highly correlated (Pearson correlations for *ZFX* "only": 0.87; *ZFY* "only": 0.92; Figure S19). Finally, all Xa-expressed genes that significantly responded to *ZFY* knockdown also responded to *ZFX* knockdown in 46,XX and 46,XY cells; even the Xa-expressed genes that were only significantly responsive to *ZFX* knockdown displayed correlated responses to *ZFY* knockdown (Figure S20). We conclude that *ZFX* and *ZFY* activate the same genes, with *ZFX* being more potent than *ZFY*, and that the total levels of *ZFX+ZFY* impact thousands of genes across the genome.

ZFX and ZFY are required for shared response to Chr X and Chr Y copy number

Finally, we asked whether *ZFX* or *ZFY* activity is required for the genome-wide response to Chr X and Chr Y copy number. Of the 136 autosomal genes responsive to both Chr X and Y copy number in fibroblasts, 109 (81%) responded to *ZFX* and/or *ZFY* knockdown, a significant enrichment (Figure 7A). Among genes that responded significantly "only" to Chr X or "only" to Chr Y, 70% and 57%, respectively, were *ZFX* or *ZFY* responsive, again suggesting that ZFX and ZFY have similar genome-wide targets (Figure S21A). On a gene-by-gene basis, the effects of *ZFX* or *ZFY* knockdown were negatively correlated with the response to Chr X or Chr Y copy number, consistent with ZFX and ZFY's functions as transcriptional activators (Figures 7B and S21B). In all, the transcriptional changes upon *ZFX* or *ZFY* knockdown explained 19%–40% of the effects for autosomal genes responsive to *either* Chr X or Y copy number, and 46%–57% of the effects for autosomal genes responsive to *both* Chr X and Y copy number (Figures 7B and S21).

We obtained similar results for genes that are only expressed from Xa. Of the seven Xa-expressed genes significantly responsive to Chr X or Y number in fibroblasts, all were significantly responsive to *ZFX* and/or *ZFY* knockdown (Figure 7C). For these seven Xa-expressed genes, the effects of *ZFX* and/or *ZFY* knockdown explain 78%–92% of the effects of Chr X and Y copy number (Figure 7D). In sum, the actions of *ZFX* and *ZFY* explain most of the shared response to Chr X and Chr Y copy number on Xa as well as on autosomes.

DISCUSSION

Traditionally, the "gene-poor" Y and "inactive" X (Xi) chromosomes have not been considered prominent players in regulating the transcriptome of human somatic cells. Studies of these "sex chromosomes" have focused instead on their capacity to differentiate the reproductive cells and organs of males and females. Our study produced two surprising findings that challenge common understandings of these chromosomes. First, the Xi and Y chromosomes modulate expression of thousands of autosomal genes in somatic cells, as we demonstrated in transformed (lymphoblastoid) cells and primary cultures (skin fibroblasts). Second, the genome-wide impact of these two chromosomes is strikingly similar: many autosomal genes respond to Xi and Y by changing expression in the same direction and with correlated effect sizes. These shared effects do not reflect a generic aneuploidy response, hormonal responses, or a response to a heterochromatin sink, and they are not driven by pseudoautosomal genes. Instead, a pair of NPX- and NPY-encoded transcriptional activators, ZFX and ZFY, account for about half of the shared effects.

Twenty-one percent of autosomal genes expressed in LCLs or fibroblasts respond to Chr X or Y copy number

We began this study by quantitatively modeling autosomal gene expression in cells of two types cultured from 176 individuals

(D) Schematic showing recombination between X and Y Chrs at *PRKX* and *PRKY*, or at *ANOS1* and *ANOS2P*, resulting in X-Y translocation products.
(E and F) Principal-component analysis (PCA) of autosomal X- and Y-responsive genes in LCL (E) or fibroblast (F) samples with one to three copies of Chr X or 46,X,i(Xq) structural variants.
(G) PCA of autosomal X- and Y-responsive genes in LCL samples with one to five total sex chromosomes or with X-Y translocated chromosomes. Ellipses represent 95% confidence intervals around the centroid of each karyotype group with at least three samples.

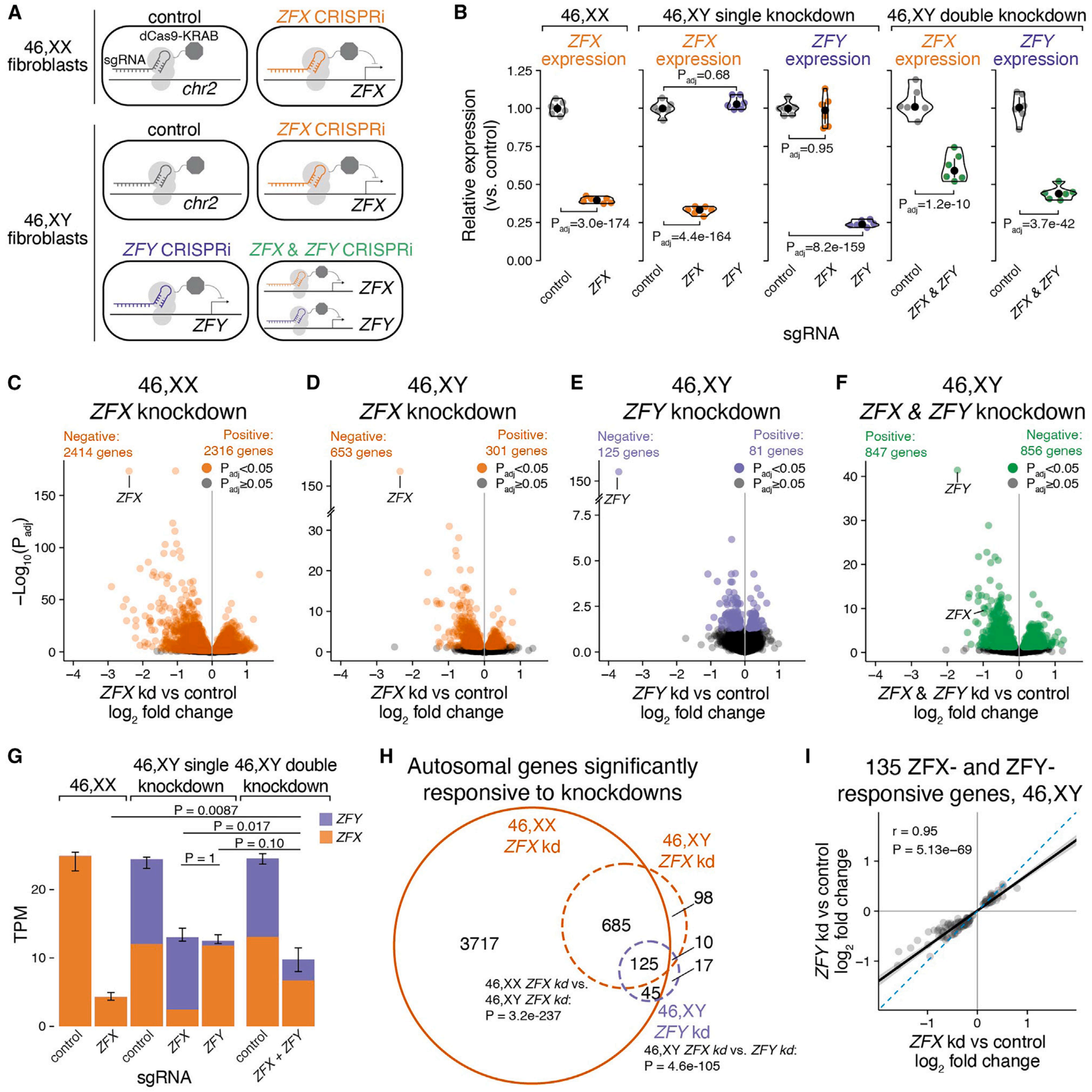

Figure 6. *ZFX* and *ZFY* activate a common set of genes

(A) Schematic of CRISPRi knockdown experiments. 46,XX and 46,XY fibroblasts from three individuals were transduced with dCas9-KRAB and sgRNAs directed against a control intergenic region on Chr 2, the promoter of *ZFX*, or the promoter of *ZFY* to block transcription.

(B) Violin plots with median and interquartile range showing relative expression of *ZFX* or *ZFY* in cells transduced with *ZFX* and/or *ZFY* sgRNAs, compared with control sgRNAs. Log_2FC and P_{adj} values from DESeq2 results.

(C–F) Volcano plots showing effect size and significance of all expressed genes in *ZFX* and/or *ZFY* knockdowns vs. controls. Numbers of significantly differentially expressed genes are indicated. Note that the Y axis limits are not the same across these plots.

(G) Stacked bar plots showing median and interquartile ranges (whiskers) of cumulative expression in transcripts per million (TPM) of *ZFX* and *ZFY* in CRISPRi experiments. Bonferroni-adjusted p values, Wilcoxon rank-sum test.

(H) Venn diagram of significantly differentially expressed autosomal genes upon knockdown of *ZFX* in XX or XY cells or upon knockdown of *ZFY* in XY cells. Bonferroni-adjusted p values, hypergeometric test.

(I) Scatterplot of significantly *ZFX*- and *ZFY*-responsive autosomal genes in 46,XY cells comparing effects of *ZFX* vs. *ZFY* knockdowns. Black line and gray shading, weighted Deming regression and 95% confidence interval; blue dashed line, identity (X = Y) line; Pearson correlation and p value are indicated.

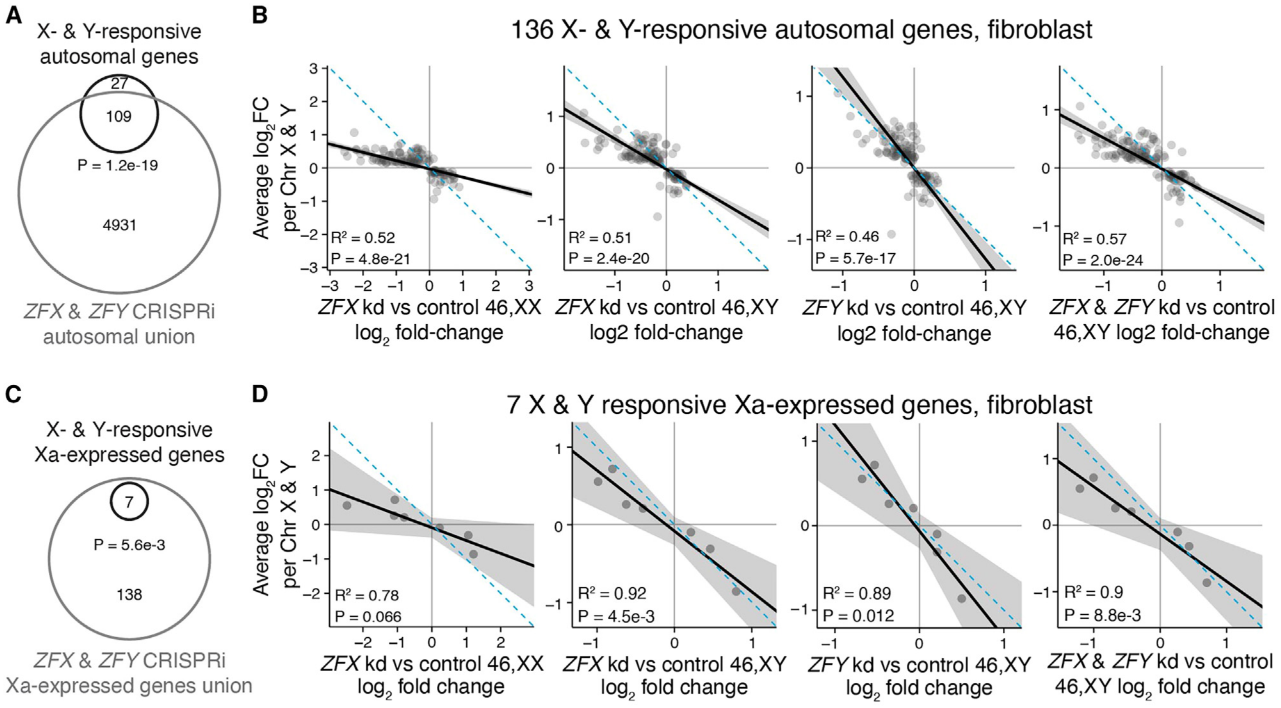

Figure 7. *ZFX* and *ZFY* are required for shared Chr X and Chr Y-responsive transcriptional program

(A and C) Venn diagram of X- and Y-responsive autosomal (A) or Xa-expressed (C) genes in fibroblasts with union of all *ZFX*- and *ZFY*-responsive genes across the four CRISPRi experiments. Bonferroni-adjusted p values, hypergeometric test.

(B and D) Scatterplots comparing response to Chr X copy number and response to *ZFX* or *ZFY* knockdown for 136 autosomal genes (B) or seven Xa-expressed genes (D) that are significantly X- and Y-responsive in fibroblasts. Black line and gray shading, weighted Deming regression and 95% confidence interval; blue dashed line, identity (X = Y) line; coefficients of determination and Bonferroni-adjusted p values are indicated.

with a wide range of sex chromosome constitutions: one to four X chromosomes (zero to three Xi chromosomes), and zero to four Y chromosomes (e.g., 45,X to 49,XXXXY and 49,XYYYY). Incorporating these diverse sex chromosome constitutions into a single linear model (Figure 1) yielded advantages over earlier studies that compared transcriptomes in pairwise fashion across a circumscribed set of sex chromosome constitutions: most frequently comparing 46,XX with 46,XY; 45,X with 46,XX; and 46,XY with 47,XXY.[12–14,36,37,39–44] Our linear model provided the power required to detect and quantify increases or decreases in expression of individual autosomal genes as a function of Chr X copy number, and as a function of Chr Y copy number. Eighteen percent of LCL-expressed autosomal genes were significantly responsive to Chr X copy number, and 6% of such genes were responsive to Chr Y copy number (Figure 1). In fibroblasts, 5% of expressed autosomal genes were significantly X-responsive, and 2% were Y-responsive. Merging these observations, we find that, of 13,126 autosomal genes expressed in LCLs and/or fibroblasts, 2,722 (20.7%) are significantly responsive to Chr X and/or Chr Y copy number in one or both cell types (Table S13). Additionally, of 335 Xa-expressed genes in LCLs and/or fibroblasts, 88 (26.3%) are significantly responsive to Chr X and/or Chr Y copy number in one or both cell types (Table S13). As a group, these Xi- or Y-responsive genes—2,722 autosomal genes and 88 Xa-expressed genes—are widely dispersed across functional categories, with only a weak enrichment for functional categories related to metabolism (Figure S22).

Importantly, we observed these effects of Xi and Y copy number even when minimizing the differentiating influence of sex steroid hormones by culturing cells under identical conditions. Moreover, we found that the effects of Xi copy number on autosomal gene expression are correlated in cells derived from anatomically male and female individuals (Figures S3E and S3F). We conclude that the Xi effects on autosomal gene expression observed here are independent of the presence of the Y chromosome or the gonadal sex of the individual donor.

Shared effects on the transcriptome reflect the common autosomal ancestry of X and Y chromosomes

The Y and X chromosomes play starkly different roles in the differentiation and function of the human reproductive tract.[45] In cultured somatic cells, however, we observed that the Y and Xi chromosomes have broadly similar and highly correlated effects on the transcriptome of all other chromosomes, including the Xa chromosome found in somatic cells of all individuals, regardless of their biological sex.

We had not anticipated these similar effects of Xi and Y on the global transcriptome, but they can be explained in light of the evolution of the human X and Y chromosomes from a pair of autosomes over the past 200 million years.[1,2] The human X and Y

chromosomes originated not as "sex chromosomes" but as ordinary autosomes, identical in sequence and, in all likelihood, largely identical in their functions in male and female somatic cells. The genes of these ancestral autosomes included global mediators and regulators of gene expression unrelated to sex differentiation—mediators and regulators that survived the differentiation of the X and Y chromosomes and remain expressed today on both Y and Xi.[10,46]

Indeed, as shown here, one of these global regulators—surviving today as *ZFX* on the human X chromosome and its homolog *ZFY* on the human Y chromosome—accounts for about half of the shared effect of Y and Xi on global gene expression. Combining the results reported here with prior discoveries, we propose the following mechanistic model: The single Xa chromosome found in all somatic cells (regardless of sex) contributes a relatively invariant level of ZFX protein; each Xi chromosome in the cell adds a smaller amount of ZFX protein, and each Y chromosome adds a similar quantity of ZFY protein. In *trans*, ZFX and ZFY proteins act additively, and function interchangeably, in modulating transcript levels for thousands of autosomal genes (and Xa genes).

The details and generalizability of this model should be tested in diverse somatic cell types. However, our findings are sufficient to state that the shared autosomal ancestry of the Xi and Y chromosomes continues to shape the functional roles of both chromosomes. Recognizing that *ZFX* (and *ZFY*) account for about half of the Xi and Y chromosomes' effects on the autosomal transcriptome in LCLs and skin fibroblasts, we encourage investigators to explore the possibility that other ancestral NPX-NPY gene pairs contribute mechanistically to sex-shared functions of the Xi and Y chromosomes.

The challenge: Distinguishing between the shared and differentiating roles of Xi and Y in human somatic cells

Our findings demonstrate that human Xi and Y chromosomes perform shared roles in modulating the autosomal (and Xa) transcriptome in somatic cells. However, myriad differences in autosomal gene expression in human somatic tissues (outside the reproductive tract) are found when comparing 46,XY males with 46,XX females.[12–14,39,40] More research is required to determine the degree to which these transcriptomic differences represent sex differences in cellular composition, sex steroid hormones, or cell-autonomous roles of the Xi and Y chromosomes. The task of searching for and molecularly substantiating sex-differentiating, cell-autonomous roles of individual Xi- or Y-expressed genes in somatic tissues, at the molecular level, will be complicated by the breadth and scale of the sex-shared, cell-autonomous roles described here.

Limitations of the study

We found a genome-wide response to Chr X and Y copy number in both LCLs and fibroblasts, but most of the autosomal genes that responded were X- and/or Y-responsive in only one of the two cell types. Thus, these autosomal "responders" will likely differ based on the epigenetic context of the cell or tissue type of interest, and this should be taken into consideration when comparing the gene lists here with metrics derived in other tissues. All experiments in this paper have been performed in cultured cells and should be validated *in vivo*. The genes directly targeted by androgen or estrogen receptors may differ from one cell type to another, so the absence of an overlap with X- and Y-responsive genes could reflect different targets in cancer cell lines versus LCLs and fibroblasts. The heterochromatin sink hypothesis was tested by studying naturally occurring variation in the Y chromosome; this hypothesis was not directly tested for the X chromosome, where we could not genetically or experimentally separate the possible effects of Xi heterochromatin from the effects of Xi gene expression.

STAR★METHODS

Detailed methods are provided in the online version of this paper and include the following:

- KEY RESOURCES TABLE
- RESOURCE AVAILABILITY
  - Lead contact
  - Materials availability
  - Data and code availability
- EXPERIMENTAL MODEL AND SUBJECT DETAILS
  - Human subjects
- METHOD DETAILS
  - Cell culture
  - RNA extraction and RNA-seq library preparation
  - RNA-seq data processing
  - Linear modeling to identify autosomal X- or Y-responsive genes
  - Saturation analysis of autosomal X- and Y-responsive genes
  - Comparing X- or Y-responsive genes to estrogen or androgen receptor target genes
  - Functional annotation
  - Chromosome 21 copy number analysis
  - Meta-analysis of published Xi expression datasets to define "Xa-expressed" genes
  - Analysis of Yq heterochromatin length
  - Analysis of gene expression in individuals with structural variants of the sex chromosomes
  - Y isochromosomes
  - X isochromosomes
  - X-Y translocations
  - Fluorescent *in situ* hybridization
  - Motif analysis
  - Analysis of ZFX ChIP-seq data
  - Identification of ZFX direct target genes from published data
  - CRISPR interference
  - Lentiviral production
  - Generation of dCas9-KRAB fibroblast cultures
  - ZFX and ZFY knockdown and analysis
- QUANTIFICATION AND STATISTICAL ANALYSIS

SUPPLEMENTAL INFORMATION

Supplemental information can be found online at https://doi.org/10.1016/j.xgen.2023.100462.

ACKNOWLEDGMENTS

We thank members of the Page laboratory for discussions, Sahin Naqvi for advice on RNA-sequencing and analysis, Jorge Adarme and Susan Tocio for laboratory support, Clare Malone for advice in optimizing the CRISPRi system, and the Whitehead Institute Genome Technology Core facility for library preparation and sequencing. We thank the individuals who contributed tissue samples for their participation.

Funding: National Institutes of Health grant F32HD091966 (A.K.S.R.), National Institutes of Health grant K23HD092588 (S.M.D.), NIH/NCATS Colorado CTSA grant UL1 TR002535 (N.R.T.). National Institutes of Health grant U01HG0007587 (D.C.P. and M.M.), National Human Genome Research Institute Intramural Research Program (M.M.), National Science Foundation Graduate Research Fellowship grant 1745302 (N.V.B.), Boehringer Ingelheim Fellowship (J.L.), Lallage Feazel Wall Damon Runyon Cancer Research Foundation Fellowship (L.V.B.), Howard Hughes Medical Institute (D.C.P.), and Simons Foundation Autism Research Initiative award 809293 (D.C.P.).

Contents are the authors' sole responsibility and do not necessarily represent official NIH views.

Philanthropic support from The Brit Jepson d'Arbeloff Center on Women's Health, Arthur W. and Carol Tobin Brill, Matthew Brill, Charles Ellis, The Brett Barakett Foundation, Howard P. Colhoun Family Foundation, Seedlings Foundation, and Knobloch Family Foundation.

AUTHOR CONTRIBUTIONS

Conceptualization: A.K.S.R. and D.C.P. Data curation: A.K.S.R. and H.S. Formal analysis: A.K.S.R., H.S., A.K.G., N.V.B., L.T., I.S., and L.V.B. Funding acquisition: A.K.S.R., N.V.B., L.V.B., S.M.D., N.R.T., M.M., and D.C.P. Investigation: A.K.S.R., T.P., J.L., N.K., L.B., S.P., A.D., and E.P. Methodology: A.K.S.R., H.S., A.K.G., L.T., D.W.B., T.P., J.L. Project administration: A.K.S.R., J.F.H., L.B., A.B., P.K., N.B., P.C.L., C.K., and S.M.D. Resources: A.B., P.K., N.B., P.C.L., C.K., S.M.D., A.E.L., N.R.T., C.S.S., and M.M. Software: A.K.S.R., H.S., A.K.G., N.V.B., L.T., I.S., and L.V.B. Supervision: A.K.S.R., J.F.H., L.B., A.E.L., N.R.T., C.S.S., M.M., and D.C.P. Validation: H.S., A.K.G., L.V.B., D.W.B. Visualization: A.K.S.R., A.K.G., and I.S. Writing—original draft preparation: A.K.S.R. and D.C.P. Writing—review and editing: A.K.S.R., H.S., A.K.G., N.V.B., L.T., I.S., L.V.B., D.W.B., J.F.H., and D.C.P.

DECLARATION OF INTERESTS

The authors declare no competing interests.

INCLUSION AND DIVERSITY

We support inclusive, diverse, and equitable conduct of research.

Received: June 1, 2023
Revised: August 15, 2023
Accepted: November 14, 2023
Published: December 13, 2023

REFERENCES

1. Ohno, S. (1967). Sex Chromosomes and Sex-Linked Genes (Springer Berlin Heidelberg).
2. Lahn, B.T., and Page, D.C. (1999). Four evolutionary strata on the human X chromosome. Science *286*, 964–967.
3. Lyon, M.F. (1961). Gene action in the X-chromosome of the mouse (Mus musculus L.). Nature *190*, 372–373.
4. Brown, C.J., Ballabio, A., Rupert, J.L., Lafreniere, R.G., Grompe, M., Tonlorenzi, R., and Willard, H.F. (1991). A gene from the region of the human X inactivation centre is expressed exclusively from the inactive X chromosome. Nature *349*, 38–44.
5. Stern, C. (1957). The problem of complete Y-linkage in man. Am. J. Hum. Genet. *9*, 147–166.
6. Skaletsky, H., Kuroda-Kawaguchi, T., Minx, P.J., Cordum, H.S., Hillier, L., Brown, L.G., Repping, S., Pyntikova, T., Ali, J., Bieri, T., et al. (2003). The male-specific region of the human Y chromosome is a mosaic of discrete sequence classes. Nature *423*, 825–837.
7. Carrel, L., and Willard, H.F. (2005). X-inactivation profile reveals extensive variability in X-linked gene expression in females. Nature *434*, 400–404.
8. Tukiainen, T., Villani, A.C., Yen, A., Rivas, M.A., Marshall, J.L., Satija, R., Aguirre, M., Gauthier, L., Fleharty, M., Kirby, A., et al. (2017). Landscape of X chromosome inactivation across human tissues. Nature *550*, 244–248.
9. Lahn, B.T., and Page, D.C. (1997). Functional coherence of the human Y chromosome. Science *278*, 675–680.
10. Bellott, D.W., Hughes, J.F., Skaletsky, H., Brown, L.G., Pyntikova, T., Cho, T.-J., Koutseva, N., Zaghlul, S., Graves, T., Rock, S., et al. (2014). Mammalian Y chromosomes retain widely expressed dosage-sensitive regulators. Nature *508*, 494–499.
11. Forsberg, L.A. (2017). Loss of chromosome Y (LOY) in blood cells is associated with increased risk for disease and mortality in aging men. Hum. Genet. *136*, 657–663.
12. Melé, M., Ferreira, P.G., Reverter, F., DeLuca, D.S., Monlong, J., Sammeth, M., Young, T.R., Goldmann, J.M., Pervouchine, D.D., Sullivan, T.J., et al. (2015). The human transcriptome across tissues and individuals. Science *348*, 660–665.
13. Naqvi, S., Godfrey, A.K., Hughes, J.F., Goodheart, M.L., Mitchell, R.N., and Page, D.C. (2019). Conservation, acquisition, and functional impact of sex-biased gene expression in mammals. Science *365*, eaaw7317.
14. Oliva, M., Muñoz-Aguirre, M., Kim-Hellmuth, S., Wucher, V., Gewirtz, A.D.H., Cotter, D.J., Parsana, P., Kasela, S., Balliu, B., Viñuela, A., et al. (2020). The impact of sex on gene expression across human tissues. Science *369*, eaba3066.
15. San Roman, A.K., Godfrey, A.K., Skaletsky, H., Bellott, D.W., Groff, A.F., Harris, H.L., Blanton, L.V., Hughes, J.F., Brown, L., Phou, S., et al. (2023). The human inactive X chromosome modulates expression of the active X chromosome. Cell Genom. *3*, 100259.
16. Love, M.I., Huber, W., and Anders, S. (2014). Moderated estimation of fold change and dispersion for RNA-seq data with DESeq2. Genome Biol. *15*, 550.
17. Guan, J., Zhou, W., Hafner, M., Blake, R.A., Chalouni, C., Chen, I.P., De Bruyn, T., Giltnane, J.M., Hartman, S.J., Heidersbach, A., et al. (2019). Therapeutic Ligands Antagonize Estrogen Receptor Function by Impairing Its Mobility. Cell *178*, 949–963.e18.
18. Cato, L., Neeb, A., Sharp, A., Buzón, V., Ficarro, S.B., Yang, L., Muhle-Goll, C., Kuznik, N.C., Riisnaes, R., Nava Rodrigues, D., et al. (2017). Development of Bag-1L as a therapeutic target in androgen receptor-dependent prostate cancer. Elife *6*, e27159.
19. Cotton, A.M., Ge, B., Light, N., Adoue, V., Pastinen, T., and Brown, C.J. (2013). Analysis of expressed SNPs identifies variable extents of expression from the human inactive X chromosome. Genome Biol. *14*, R122.
20. Garieri, M., Stamoulis, G., Blanc, X., Falconnet, E., Ribaux, P., Borel, C., Santoni, F., and Antonarakis, S.E. (2018). Extensive cellular heterogeneity of X inactivation revealed by single-cell allele-specific expression in human fibroblasts. Proc. Natl. Acad. Sci. USA *115*, 13015–13020.
21. Sauteraud, R., Stahl, J.M., James, J., Englebright, M., Chen, F., Zhan, X., Carrel, L., and Liu, D.J. (2021). Inferring genes that escape X-Chromosome inactivation reveals important contribution of variable escape genes to sex-biased diseases. Genome Res. *31*, 1629–1637.
22. Balaton, B.P., Cotton, A.M., and Brown, C.J. (2015). Derivation of consensus inactivation status for X-linked genes from genome-wide studies. Biol. Sex Differ. *6*, 35.
23. Siegel, J.J., and Amon, A. (2012). New Insights into the Troubles of Aneuploidy. Annu. Rev. Cell Dev. Biol. *28*, 189–214.

CellPress
OPEN ACCESS

24. Lemos, B., Branco, A.T., and Hartl, D.L. (2010). Epigenetic effects of polymorphic Y chromosomes modulate chromatin components, immune response, and sexual conflict. Proc. Natl. Acad. Sci. USA *107*, 15826–15831.
25. Repping, S., van Daalen, S.K.M., Brown, L.G., Korver, C.M., Lange, J., Marszalek, J.D., Pyntikova, T., van der Veen, F., Skaletsky, H., Page, D.C., and Rozen, S. (2006). High mutation rates have driven extensive structural polymorphism among human Y chromosomes. Nat. Genet. *38*, 463–467.
26. 1000 Genomes Project Consortium; Auton, A., Brooks, L.D., Durbin, R.M., Garrison, E.P., Kang, H.M., Korbel, J.O., Marchini, J.L., McCarthy, S., McVean, G.A., and Abecasis, G.R. (2015). The 1000 Genomes Project Consortium (2015). A global reference for human genetic variation. Nature *526*, 68–74.
27. Lappalainen, T., Sammeth, M., Friedländer, M.R., t Hoen, P.A.C., Monlong, J., Rivas, M.A., Gonzàlez-Porta, M., Kurbatova, N., Griebel, T., Ferreira, P.G., et al. (2013). Transcriptome and genome sequencing uncovers functional variation in humans. Nature *501*, 506–511.
28. Weil, D., Wang, I., Dietrich, A., Poustka, A., Weissenbach, J., and Petit, C. (1994). Highly homologous loci on the X and Y chromosomes are hot-spots for ectopic recombinations leading to XX maleness. Nat. Genet. *7*, 414–419.
29. Schneider-Gädicke, A., Beer-Romero, P., Brown, L.G., Nussbaum, R., and Page, D.C. (1989). ZFX has a gene structure similar to ZFY, the putative human sex determinant, and escapes X inactivation. Cell *57*, 1247–1258.
30. Palmer, M.S., Berta, P., Sinclair, A.H., Pym, B., and Goodfellow, P.N. (1990). Comparison of human ZFY and ZFX transcripts. Proc. Natl. Acad. Sci. USA *87*, 1681–1685.
31. Ni, W., Perez, A.A., Schreiner, S., Nicolet, C.M., and Farnham, P.J. (2020). Characterization of the ZFX family of transcription factors that bind downstream of the start site of CpG island promoters. Nucleic Acids Res. *48*, 5986–6000.
32. Rhie, S.K., Yao, L., Luo, Z., Witt, H., Schreiner, S., Guo, Y., Perez, A.A., and Farnham, P.J. (2018). ZFX acts as a transcriptional activator in multiple types of human tumors by binding downstream from transcription start sites at the majority of CpG island promoters. Genome Res. *28*, 310–320.
33. Decarpentrie, F., Vernet, N., Mahadevaiah, S.K., Longepied, G., Streichemberger, E., Aknin-Seifer, I., Ojarikre, O.A., Burgoyne, P.S., Metzler-Guillemain, C., and Mitchell, M.J. (2012). Human and mouse ZFY genes produce a conserved testis-specific transcript encoding a zinc finger protein with a short acidic domain and modified transactivation potential. Hum. Mol. Genet. *21*, 2631–2645.
34. Mardon, G., Luoh, S.W., Simpson, E.M., Gill, G., Brown, L.G., and Page, D.C. (1990). Mouse Zfx protein is similar to Zfy-2: each contains an acidic activating domain and 13 zinc fingers. Mol. Cell Biol. *10*, 681–688.
35. Xu, S., Duan, P., Li, J., Senkowski, T., Guo, F., Chen, H., Romero, A., Cui, Y., Liu, J., and Jiang, S.W. (2016). Zinc Finger and X-Linked Factor (ZFX) Binds to Human SET Transcript 2 Promoter and Transactivates SET Expression. Int. J. Mol. Sci. *17*, 1737.
36. Raznahan, A., Parikshak, N.N., Chandran, V., Blumenthal, J.D., Clasen, L.S., Alexander-Bloch, A.F., Zinn, A.R., Wangsa, D., Wise, J., Murphy, D.G.M., et al. (2018). Sex-chromosome dosage effects on gene expression in humans. Proc. Natl. Acad. Sci. USA *115*, 7398–7403.
37. Zhang, X., Hong, D., Ma, S., Ward, T., Ho, M., Pattni, R., Duren, Z., Stankov, A., Bade Shrestha, S., Hallmayer, J., et al. (2020). Integrated functional genomic analyses of Klinefelter and Turner syndromes reveal global network effects of altered X chromosome dosage. Proc. Natl. Acad. Sci. USA *117*, 4864–4873.
38. Rosenbluh, J., Xu, H., Harrington, W., Gill, S., Wang, X., Vazquez, F., Root, D.E., Tsherniak, A., and Hahn, W.C. (2017). Complementary information derived from CRISPR Cas9 mediated gene deletion and suppression. Nat. Commun. *8*. ncomms15403.
39. Gershoni, M., and Pietrokovski, S. (2017). The landscape of sex-differential transcriptome and its consequent selection in human adults. BMC Biol. *15*, 7.
40. Lopes-Ramos, C.M., Chen, C.Y., Kuijjer, M.L., Paulson, J.N., Sonawane, A.R., Fagny, M., Platig, J., Glass, K., Quackenbush, J., and DeMeo, D.L. (2020). Sex Differences in Gene Expression and Regulatory Networks across 29 Human Tissues. Cell Rep. *31*, 107795.
41. Trolle, C., Nielsen, M.M., Skakkebæk, A., Lamy, P., Vang, S., Hedegaard, J., Nordentoft, I., Ørntoft, T.F., Pedersen, J.S., and Gravholt, C.H. (2016). Widespread DNA hypomethylation and differential gene expression in Turner syndrome. Sci. Rep. *6*. 34220-34214.
42. Nielsen, M.M., Trolle, C., Vang, S., Hornshøj, H., Skakkebaek, A., Hedegaard, J., Nordentoft, I., Pedersen, J.S., and Gravholt, C.H. (2020). Epigenetic and transcriptomic consequences of excess X-chromosome material in 47,XXX syndrome-A comparison with Turner syndrome and 46,XX females. Am. J. Med. Genet. C Semin. Med. Genet. *184*, 279–293.
43. Viuff, M., Skakkebæk, A., Johannsen, E.B., Chang, S., Pedersen, S.B., Lauritsen, K.M., Pedersen, M.G.B., Trolle, C., Just, J., and Gravholt, C.H. (2023). X chromosome dosage and the genetic impact across human tissues. Genome Med. *15*, 21.
44. Liu, S., Akula, N., Reardon, P.K., Russ, J., Torres, E., Clasen, L.S., Blumenthal, J., Lalonde, F., McMahon, F.J., Szele, F., et al. (2023). Aneuploidy effects on human gene expression across three cell types. Proc. Natl. Acad. Sci. USA *120*, e2218478120.
45. Hughes, J.F., and Page, D.C. (2015). The Biology and Evolution of Mammalian Y Chromosomes. Annu. Rev. Genet. *49*, 507–527.
46. Bellott, D.W., and Page, D.C. (2021). Dosage-sensitive functions in embryonic development drove the survival of genes on sex-specific chromosomes in snakes, birds, and mammals. Genome Res. *31*, 198–210.
47. Godfrey, A.K., Naqvi, S., Chmátal, L., Chick, J.M., Mitchell, R.N., Gygi, S.P., Skaletsky, H., and Page, D.C. (2020). Quantitative analysis of Y-Chromosome gene expression across 36 human tissues. Genome Res. *30*, 860–873.
48. Subramanian, A., Tamayo, P., Mootha, V.K., Mukherjee, S., Ebert, B.L., Gillette, M.A., Paulovich, A., Pomeroy, S.L., Golub, T.R., Lander, E.S., and Mesirov, J.P. (2005). Gene set enrichment analysis: a knowledge-based approach for interpreting genome-wide expression profiles. Proc. Natl. Acad. Sci. USA *102*, 15545–15550.
49. Liberzon, A., Subramanian, A., Pinchback, R., Thorvaldsdóttir, H., Tamayo, P., and Mesirov, J.P. (2011). Molecular signatures database (MSigDB) 3.0. Bioinformatics *27*, 1739–1740.
50. Horlbeck, M.A., Gilbert, L.A., Villalta, J.E., Adamson, B., Pak, R.A., Chen, Y., Fields, A.P., Park, C.Y., Corn, J.E., Kampmann, M., and Weissman, J.S. (2016). Compact and highly active next-generation libraries for CRISPR-mediated gene repression and activation. Elife *5*, e19760.
51. Price, C., Gill, S., Ho, Z.V., Davidson, S.M., Merkel, E., McFarland, J.M., Leung, L., Tang, A., Kost-Alimova, M., Tsherniak, A., et al. (2019). Genome-Wide Interrogation of Human Cancers Identifies EGLN1 Dependency in Clear Cell Ovarian Cancers. Cancer Res. *79*, 2564–2579.
52. Bray, N.L., Pimentel, H., Melsted, P., and Pachter, L. (2016). Near-optimal probabilistic RNA-seq quantification. Nat. Biotechnol. *34*, 525–527.
53. Langmead, B., and Salzberg, S.L. (2012). Fast gapped-read alignment with Bowtie 2. Nat. Methods *9*, 357–359.
54. Zhang, Y., Liu, T., Meyer, C.A., Eeckhoute, J., Johnson, D.S., Bernstein, B.E., Nusbaum, C., Myers, R.M., Brown, M., Li, W., and Liu, X.S. (2008). Model-based analysis of ChIP-Seq (MACS). Genome Biol. *9*, R137.
55. Quinlan, A.R., and Hall, I.M. (2010). BEDTools: a flexible suite of utilities for comparing genomic features. Bioinformatics *26*, 841–842.
56. Heinz, S., Benner, C., Spann, N., Bertolino, E., Lin, Y.C., Laslo, P., Cheng, J.X., Murre, C., Singh, H., and Glass, C.K. (2010). Simple combinations of lineage-determining transcription factors prime cis-regulatory elements required for macrophage and B cell identities. Mol. Cell *38*, 576–589.

57. Metsalu, T., and Vilo, J. (2015). ClustVis: a web tool for visualizing clustering of multivariate data using Principal Component Analysis and heatmap. Nucleic Acids Res. *43*, W566–W570.

58. Pruitt, K.D., Harrow, J., Harte, R.A., Wallin, C., Diekhans, M., Maglott, D.R., Searle, S., Farrell, C.M., Loveland, J.E., Ruef, B.J., et al. (2009). The consensus coding sequence (CCDS) project: Identifying a common protein-coding gene set for the human and mouse genomes. Genome Res. *19*, 1316–1323.

59. Soneson, C., Love, M.I., and Robinson, M.D. (2015). Differential analyses for RNA-seq: transcript-level estimates improve gene-level inferences. F1000Res. *4*, 1521.

60. Teitz, L.S., Pyntikova, T., Skaletsky, H., and Page, D.C. (2018). Selection Has Countered High Mutability to Preserve the Ancestral Copy Number of Y Chromosome Amplicons in Diverse Human Lineages. Am. J. Hum. Genet. *103*, 261–275.

61. Dohm, J.C., Lottaz, C., Borodina, T., and Himmelbauer, H. (2008). Substantial biases in ultra-short read data sets from high-throughput DNA sequencing. Nucleic Acids Res. *36*, e105.

62. ’t Hoen, P.A.C., Friedländer, M.R., Almlöf, J., Sammeth, M., Pulyakhina, I., Anvar, S.Y., Laros, J.F.J., Buermans, H.P.J., Karlberg, O., Brännvall, M., et al. (2013). Reproducibility of high-throughput mRNA and small RNA sequencing across laboratories. Nat. Biotechnol. *31*, 1015–1022.

63. Lange, J., Skaletsky, H., van Daalen, S.K.M., Embry, S.L., Korver, C.M., Brown, L.G., Oates, R.D., Silber, S., Repping, S., and Page, D.C. (2009). Isodicentric Y chromosomes and sex disorders as byproducts of homologous recombination that maintains palindromes. Cell *138*, 855–869.

64. Lange, J., Noordam, M.J., van Daalen, S.K.M., Skaletsky, H., Clark, B.A., Macville, M.V., Page, D.C., and Repping, S. (2013). Intrachromosomal homologous recombination between inverted amplicons on opposing Y-chromosome arms. Genomics *102*, 257–264.

65. Ritchie, M.E., Phipson, B., Wu, D., Hu, Y., Law, C.W., Shi, W., and Smyth, G.K. (2015). limma powers differential expression analyses for RNA-sequencing and microarray studies. Nucleic Acids Res. *43*, e47.

66. Schiebel, K., Winkelmann, M., Mertz, A., Xu, X., Page, D.C., Weil, D., Petit, C., and Rappold, G.A. (1997). Abnormal XY interchange between a novel isolated protein kinase gene, PRKY, and its homologue, PRKX, accounts for one third of all (Y+)XX males and (Y-)XY females. Hum. Mol. Genet. *6*, 1985–1989.

67. Chen, B., Gilbert, L.A., Cimini, B.A., Schnitzbauer, J., Zhang, W., Li, G.-W., Park, J., Blackburn, E.H., Weissman, J.S., Qi, L.S., and Huang, B. (2013). Dynamic Imaging of Genomic Loci in Living Human Cells by an Optimized CRISPR/Cas System. Cell *155*, 1479–1491.

68. Fulco, C.P., Munschauer, M., Anyoha, R., Munson, G., Grossman, S.R., Perez, E.M., Kane, M., Cleary, B., Lander, E.S., and Engreitz, J.M. (2016). Systematic mapping of functional enhancer–promoter connections with CRISPR interference. Science *354*, 769–773.

69. Stewart, S.A., Dykxhoorn, D.M., Palliser, D., Mizuno, H., Yu, E.Y., An, D.S., Sabatini, D.M., Chen, I.S.Y., Hahn, W.C., Sharp, P.A., et al. (2003). Lentivirus-delivered stable gene silencing by RNAi in primary cells. RNA *9*, 493–501.

70. R Development Core Team (2020). R: A Language and Environment for Statistical Computing (R Foundation for Statistical Computing).

STAR★METHODS

KEY RESOURCES TABLE

REAGENT or RESOURCE	SOURCE	IDENTIFIER
Experimental models: Cell lines		
Lymphoblastoid cell lines and primary fibroblast cell cultures	This paper	N/A
Lymphoblastoid cell lines	Colorado Children's Hospital Biobank	N/A
Lymphoblastoid cell lines and primary fibroblast cell cultures	Coriell Cell Repository	See Table S1
EBV-producing lymphoblasts	Coriell Cell Repository	B95-8 (RRID:CVCL_1953)
Lenti-X 293T cells	Takara	Cat# 632180
Bacterial strains		
Stable cells	New England Biolabs	Cat# C3040I
Chemicals, peptides, and recombinant proteins		
Percoll	Cytiva	Cat# 17-0891-01
RPMI 1640	Gibco	Cat# 31800-089
HEPES	SAFC	Cat# RES6008H-A702X
FBS	Hyclone	Cat# SH30071
Amphotericin B	Gibco	Cat# 15290-018
Gentamicin	Gibco	Cat# 15710-072
Penicillin-Streptomycin	Lonza	Cat# 11140-076
Cyclosporine	LC Laboratories	Cat# C-6000
DMEM/F12	Gibco	Cat# 11320-033
DMEM High Glucose	Gibco	Cat# 11960-069
L-Glutamine	MP Biomedicals	Cat# IC10180683
MEM Non-essential amino acids	Gibco	Cat# 15140-163
Gelatin	Sigma	Cat# G2500
TRIzol	ThermoFisher	Cat# 15596026
RNAprotect Cell Reagent	Qiagen	Cat# 76526
TransIT-LT1 Transfection Reagent	Mirus Bio	Cat# MIR2305
FastDigest Esp3I (BsmBI)	ThermoFisher	Cat# FD0454
FastAP	ThermoFisher	Cat# EF0654
T4 polynucleotide kinase	New England Biolabs	Cat# M0201S
Quick Ligase	New England Biolabs	Cat# M2200S
Lenti-X concentrator	Takara	Cat# 631231
Viral boost reagent	Alstem Bio	Cat# VB100
Polybrene (hexamethrine bromide)	Sigma	Cat# H9268
Blasticidin	Gibco	Cat# A1113903
Puromycin	Sigma	Cat# P9620
Cell Titer Glo reagent	Promega	Cat# G7570
Biotin-dUTP	Sigma	Cat# 11093070910
Cy3-dUTP	Amersham	Cat# PA53022
Avidin-FITC	Invitrogen	Cat# A821
Critical commercial assays		
Vacutainer ACD Tubes	BD Biosciences	Cat# 364606
MycoAlert Kit	Lonza	Cat# LT07-318
SapphireAmp Fast PCR Master Mix	Takara	Cat# RR350A

(*Continued on next page*)

Continued		
REAGENT or RESOURCE	SOURCE	IDENTIFIER
RNeasy Plus Mini Kit	Qiagen	Cat# 74134
QIAshredder Columns	Qiagen	Cat# 79654
Qubit RNA HS Assay Kit	ThermoFisher	Cat# Q32855
Fragment Analyzer RNA Kit	Agilent	Cat# DNF-471
HS NGS Fragment Kit	Agilent	Cat # DNF-474
TruSeq RNA Library Preparation Kit v2	Illumina	Cat# RS-122-2001
KAPA mRNA HyperPrep Kit	Roche	Cat# KK8581
PippinHT 2% Agarose Gel Cassettes	Sage Science	Cat# HTC2010
EndoFree Maxi Kit	Qiagen	Cat# 12362
QIAquick Gel Extraction Kit	Qiagen	Cat# 28704
Lenti-X GoStiX	Takara	Cat# 631280
Deposited data		
Raw, de-identified RNA-seq data	This paper	dbGaP: phs002481.v3.p1
Processed data	This paper	https://doi.org/10.5281/zenodo.10042162
Custom GENCODE v24 transcriptome annotation	Godfrey et al., 2020[47]	https://doi.org/10.5281/zenodo.3627233
RNA-seq and estrogen receptor alpha ChIP-seq in MCF7 cells with and without estradiol	Guan et al. 2019[17]	GSE117943
RNA-seq and androgen receptor ChIP-seq in LNCaP cells with and without testosterone	Cato et al. 2017[18]	GSE89939
Molecular Signatures Database (MSigDB) v2022.1.Hs	Subramanian et al. 2005[48] and Liberzon et al., 2011[49]	https://www.gsea-msigdb.org/gsea/msigdb/
Allelic expression data in fibroblasts and hybrid cell lines	Carrel et al., 2005[7]	N/A
Allelic expression data in LCLs and fibroblasts from paired genomic and cDNA SNP-chips	Cotton et al., 2013[19]	N/A
Allelic expression data from bulk and single LCLs in GTEx	Tukiainen et al., 2017[8]	N/A
Allelic expression data in single fibroblasts	Garieri et al., 2018[20]	N/A
Allelic expression data in bulk LCLs	Sauteraud et al., 2021[21]	N/A
Meta-analysis of expression from the inactive X chromosome	Balaton et al., 2015[22]	N/A
1000 Genomes Project whole genome sequencing data	The 1000 Genomes Project Consortium, 2015[26]	https://www.internationalgenome.org/
Geuvadis RNA-seq data	Lappalainen et al. 2013[27]	https://www.internationalgenome.org/data-portal/data-collection/geuvadis
ZFX ChIP-seq, HCT116	ENCODE	ENCFF858YWR, ENCFF102UZB
ZFX ChIP-seq, C4-2B	ENCODE	ENCFF160AXQ, ENCFF264NUJ
ZFX ChIP-seq, MCF-7	ENCODE	ENCFF861DOL, ENCFF429AXS
ZFX ChIP-seq, HEK-293T	ENCODE	ENCFF292HYE, ENCFF625XHP
RNA-seq, HCT-116	ENCODE	ENCFF435PHM
ZFX knockdown and control RNA-seq, C4-2B	Rhie et al., 2018[32]	GSE102616
RNA-seq, MCF-7	ENCODE	ENCFF721BRA
ZFX knockdown and control RNA-seq, MCF-7	Rhie et al., 2018[32]	GSE102616
ZFX knockout and control RNA-seq, HEK-293T	Ni et al., 2020[31]	GSE145160
Recombinant DNA		
pLX_311-KRAB-dCas9	Addgene	Cat# 96918
sgOpti	Addgene	Cat# 85681
psPAX2	Addgene	Cat# 12260
pCMV-VSV-G	Addgene	Cat# 8454
Whole X or Y chromosome FISH probes	Applied Spectral Imaging	Cat# FPRPR0167

(*Continued on next page*)

REAGENT or RESOURCE	SOURCE	IDENTIFIER
Continued		
Bacterial artificial chromosomes used as FISH probes	See Table S7C	N/A
Oligonucleotides		
ERCC RNA Sike-In Mix	Invitrogen	Cat#: 4456740
Mycoplasma primer, F: CTT CWT CGA CTT YCA GAC CCA AGG CAT	This paper	N/A
Mycoplasma primer, R: ACA CCA TGG GAG YTG GTA AT	This paper	N/A
hGAPDH primer, F: TGT CGC TGT TGA AGT CAG AGG AGA	This paper	N/A
hGAPDH primer, R: AGA ACA TCA TCC CTG CCT CTA CTG	This paper	N/A
See methods for table of gRNA sequences and primers used in this study	This paper; Horlbeck et al., 2016[50]; Price et al., 2019[51]	N/A
Software and algorithms		
Custom code to process RNA-seq data and generate figures	This paper	https://doi.org/10.5281/zenodo.10042162
R v3.6.3	The R Foundation	https://www.r-project.org
Kallisto v0.42.5	Bray et al., 2016[52]	https://pachterlab.github.io/kallisto/
DESeq2 v1.26.0	Love et al., 2014[16]	https://bioconductor.org/packages/release/bioc/html/DESeq2.html
Bowtie2 v2.3.4.1	Langmead and Salzberg, 2012[53]	https://bowtie-bio.sourceforge.net/bowtie2/index.shtml
MACS2 v2.2.7.1	Zhang et al. 2008[54]	https://github.com/macs3-project/MACS
BEDTools v2.26.0	Quinlan et al., 2010[55]	https://bedtools.readthedocs.io/
Homer v4.11.1	Heinz et al., 2010[56]	http://homer.ucsd.edu/homer/
Deming R package v1.4	Terry Therneau	https://cran.r-project.org/web/packages/deming/index.html
ClustVis beta	Metsalu et al., 2015[57]	https://biit.cs.ut.ee/clustvis/
Illustrator	Adobe	https://www.adobe.com/products/illustrator.html
Other		
QuBit 4 Fluorometer	ThermoFisher	N/A
5200 Fragment Analyzer System	Agilent	N/A
PippinHT system	Sage Sciences	N/A
HiSeq 2500	Illumina	N/A
NovaSeq 6000	Illumina	N/A
DeltaVision deconvolution microscope	GE Healthcare	N/A

RESOURCE AVAILABILITY

Lead contact

Further information and requests for resources and reagents should be directed to and will be fulfilled by the lead contact, David C. Page (dcpage@wi.mit.edu).

Materials availability

Cell lines generated in this study are available upon request to the lead contact.

Data and code availability

- Raw, de-identified RNA-sequencing data from human cell cultures has been deposited to dbGaP under accession number phs002481.v3.p1, and processed data has been deposited at Zenodo under accession number https://doi.org/10.5281/zenodo.10042162.

- This paper analyzes existing, publicly available data. Accession numbers for these datasets are listed in the key resources table.
- Original code has been deposited at Zenodo under accession number https://doi.org/10.5281/zenodo.10042162 and is publicly available as of the date of publication.
- Any additional information required to reanalyze the data reported in this paper is available from the lead contact upon request.

EXPERIMENTAL MODEL AND SUBJECT DETAILS

Human subjects

As part of an IRB-approved study at the NIH Clinical Center (12-HG-0181) and Whitehead Institute/MIT (Protocol # 1706013503), we recruited human subjects through the NIH Clinical Center, the Focus Foundation, and Mass General Hospital. We included individuals with a previous karyotype showing non-mosaic sex chromosome aneuploidy or a structural variant of the sex chromosomes, as well as euploid "healthy volunteers". We obtained informed consent from all study participants. For derivation of cell cultures and analysis, we collected blood samples and/or skin biopsies and shipped them to the Page lab. We performed karyotyping of peripheral blood and fibroblast cell cultures at the National Human Genome Research Institute Cytogenetics and Microscopy Core. We obtained additional cell lines from the Colorado Children's Hospital and Coriell Research Institute, and cultured them for at least two passages prior to RNA-sequencing. The analysis presented represents the combined analysis of samples published for the first time here, and a set of previously-published samples collected during the course of the same study.[15] We provide information about all samples analyzed in this study in Table S1.

METHOD DETAILS

Cell culture

We derived and cultured lymphoblastoid cell lines (LCLs) and skin fibroblasts as previously described.[15] Briefly, we collected blood in BD Vacutainer ACT blood collection tubes and placed two 4mm skin punch biopsies from the upper arm in transport media (DMEM/F12, 20% FBS, and 100 IU/mL Penicillin-Streptomycin [all ThermoFisher]) and shipped these to the Page lab 1–3 days after collection. We subjected blood to density gradient centrifugation to obtain lymphocytes, which we then transformed by culturing in complete RPMI (RPMI 1640 [Gibco], 25mM HEPES, 15% FBS [Hyclone], 1.25ug/ml Fungizone [Gibco], 3.33ug/ml Gentamycin [Gibco], 100 IU/mL Penicillin-Streptomycin [Lonza], pH 7.2) supplemented with EBV and 66.6 μg/ml cyclosporine until we observed cell clumping and proliferation. We minced skin biopsies and placed them on gelatin-coated plates with growth media (High Glucose DMEM [Gibco], 20% FBS [Hyclone], 2mM L-Glutamine [MP Biomedicals], 1X MEM Non-Essential Amino Acids [Gibco], 100 IU/mL Penicillin-Streptomycin [Lonza]), leaving undisturbed until fibroblasts grew out of the biopsies and could be passaged. For each sample, we collected and froze one million cells in RNAprotect Cell Reagent (Qiagen) at −80°C until RNA extraction. We confirmed that cell cultures were negative for mycoplasma contamination periodically using either the MycoAlert Kit (Lonza) or PCR, as described previously.[15]

RNA extraction and RNA-seq library preparation

We extracted RNA using the RNeasy Protect Cell Mini Kit (Qiagen), per the manufacturer's instructions, using the following modifications: we added 10 μL β-mercaptoethanol per mL of buffer RLT for cell lysis, and 10 μL of a 1:100 dilution of ERCC RNA Spike-In Mix (Invitrogen) per million cells. We homogenized lysates using QIAshredder columns (Qiagen), removed genomic DNA using gDNA eliminator columns, performed all optional spin steps, and eluted RNA in 30 μL RNase-free water. We quantified RNA using a Qubit 2.0 or 4.0 fluorometer and the Qubit RNA HS Assay Kit (ThermoFisher). RNA was consistently high-quality with RNA integrity numbers (RIN) near 10 as measured on a Bioanalyzer 2100 instrument (Agilent). We prepared RNA sequencing libraries using the TruSeq RNA Library Preparation Kit v2 (Illumina) with modifications as detailed in Naqvi et al.[13] or using the KAPA mRNA HyperPrep Kit (Roche). In both cases, we size-selected libraries using the PippinHT system (Sage Science) and 2% agarose gels with a capture window of 300-600bp. We performed paired-end 100x100 bp sequencing on a HiSeq 2500 or NovaSeq 6000 (Illumina). We indicate the library preparation kit and sequencing platform for each sample in Table S1.

RNA-seq data processing

We performed all analyses using human genome build hg38, and a custom version of the comprehensive GENCODE v24 transcriptome annotation as in Godfrey et al.[47] This annotation represents the union of the "GENCODE Basic" annotation and transcripts recognized by the Consensus Coding Sequence project.[58] To analyze samples in which ERCC spike-ins were added, we merged our custom transcript annotation with the ERCC Control annotation.

To pseudoalign reads to the transcriptome annotation and estimate expression levels of each transcript, we used kallisto software (v0.42.5).[52] We included the –bias flag to correct for sequence bias. We imported the resulting count data (abundance.tsv file) into R for analysis in DESeq2 (v1.26.0)[16] using the tximport package (v1.14.0).[59] Although we pseudoaligned and performed DESeq2 on the

entire custom transcriptome annotation, we restricted our downstream analysis to protein-coding and long non-coding RNA genes, as annotated in ensembl v107. We filtered the final analyses for genes with median expression level in XX or XY samples of at least 1 transcript per million (TPM) in a given cell type.

Linear modeling to identify autosomal X- or Y-responsive genes

We performed linear modeling in DESeq2 with three covariates: Chr X copy number, Chr Y copy number, and library preparation batch. The "$\log_2$ fold change" values output from DESeq2 in these analyses refer to the $\log_2$ fold change in expression per copy of Chr X or Y. We considered genes with $P_{adj} < 0.05$ as significantly Chr X-responsive or Chr Y-responsive.

To control for the possibility that bulk differences in sex chromosome gene expression may lead to global effects during normalization, we performed an identical analysis, removing Chr X and Y genes prior to DESeq2 normalization and linear modeling. In this analysis, we refit the same model on the renormalized data. This had little effect on the overall results.

We compared significantly X-responsive or Y-responsive genes between LCLs and fibroblasts by first restricting our analysis to autosomal genes expressed in both LCLs and fibroblasts, and then intersecting the significant gene lists. To compute p values of the overlaps, we used hypergeometric tests, with $p < 0.05$ considered significant. We calculated Pearson correlations between $\log_2$ fold change per X or Y Chr in LCLs and fibroblasts on the overlapping sets of genes.

Saturation analysis of autosomal X- and Y-responsive genes

For LCLs and fibroblasts separately, we randomly sampled without replacement size-*n* subsets of available RNA-seq libraries. We ran DESeq2 on these subsets as in the full analysis, and recorded the number of significantly Chr X- and Y-responsive expressed autosomal genes ($P_{adj} < 0.05$). We performed 100 down-samplings for each sample size, *n*.

Comparing X- or Y-responsive genes to estrogen or androgen receptor target genes

We defined direct target genes of the estrogen and androgen receptors as genes that change in expression and are bound by estrogen receptor alpha (ERα) or androgen receptor (AR) within 30 kb of the transcription start site upon addition of 17-β estradiol (E2) or dihydrotestosterone (DHT), respectively. For ERα target genes, we reanalyzed RNA-seq and ChIP-seq data in MCF7 breast cancer cells from Guan et al.,[17] and for AR target genes we reanalyzed RNA-seq and ChIP-seq data in LNCaP prostate cancer cells from Cato et al.[18] For RNA-seq data, we used the same processing pipeline as above—pseudoalignment and quantification of transcript abundance with kallisto and differential expression analysis (treatment versus vehicle) in DESeq2—with genes with $P_{adj} < 0.05$ considered significantly responsive to E2 or DHT.

For AR and ER ChIP-seq data, we aligned the reads to the human genome (hg38) using bowtie2 (v2.3.4.1)[53] with one mismatch allowed (-N 1) and called peaks using the MACS2 (v2.2.7.1)[54] callpeak function with default parameters. We called peaks in hormone treated data and vehicle control, using the matched input samples as the background control. We then selected peaks exclusively found in the treated condition compared to the vehicle control. We associated peaks with genes in our custom GENCODE v24 transcriptome annotation using the bedtools (v2.26.0) closest function with default parameters. We considered genes with a peak within 30 kb of their transcription start site "bound" by a given factor.

Functional annotation

We performed functional gene category analysis on autosomal genes significantly responsive to Chr X or Y copy number using the Molecular Signatures Database (MSigDB) Gene Ontology Biological Process categories, version 2022.1.Hs.[48,49] To test for enrichment of gene categories, we performed hypergeometric tests using all expressed autosomal genes in each cell type as the background set. We corrected the resulting p values for multiple hypothesis testing using the Benjamini-Hochberg method, with categories below the FDR threshold of 0.05 considered significantly enriched.

Chromosome 21 copy number analysis

To assess and compare the response to aneuploidy for an autosome with our sex chromosome aneuploidy model, we obtained six lymphoblastoid cell lines with Trisomy 21 from the Coriell Institute for Medical Research (AG09802, AG10316, AG10317, AG17485, AG13455, and AG16945; see Table S1 for more details). We cultured these LCLs in the laboratory for two passages and performed RNA-sequencing in parallel with the sex chromosome aneuploidy lines. We processed raw RNA-sequencing data as above. We used data from 46,XX, 46,XY, 47,XX+21, and 47,XY+21 to evaluate the effects of three versus two copies of Chr 21. We performed this analysis in DESeq2 using three covariates in a linear model: sex chromosome constitution (XX vs. XY), Chr 21 copy number (two vs. three), and library preparation batch. We previously analyzed effects of Chr 21 copy number on the expression of genes encoded on Chr 21, finding that ~3/4 significantly increased in expression.[15] Here, we compare the effects of Chr 21 copy number on the other autosomes to those of Chr X or Chr Y copy number.

Meta-analysis of published Xi expression datasets to define "Xa-expressed" genes

To define "Xa-expressed" genes, which have no evidence of expression from Xi, we compiled data from five studies of Chr X allelic ratios for 965 annotated protein-coding and long non-coding RNAs.[8,15,19–21]

The first dataset (Additional file 7 in Cotton et al.)[19] was derived from paired genomic and cDNA SNP-chips in skewed LCL and fibroblast cell cultures. We used the provided mean AR values for 416 genes informative in at least 5 samples, their standard deviations, and the number of informative samples, to test whether the AR values were significantly greater than zero with one-sample, one-sided t-tests. We corrected the resulting p values for multiple comparisons with the p.adjust function in R using the Benjamini-Hochberg method. Genes with adjusted p values (Padj) below 0.05 were considered expressed from Xi ("escape").

The second dataset was derived from bulk or single-cell RNA-seq of LCLs (Tables S5 and S8 from Tukiainen et al.).[8] The bulk RNA-seq was from one individual in the GTEx dataset with 100% skewed X inactivation across the body, and the single-cell RNA-seq in LCLs was from three individuals (we excluded data from one dendritic cell sample). We included 82 genes with data from at least two individuals in the single-cell dataset, or one individual in the single-cell dataset and data from the bulk RNA-seq dataset. Using the ratio of read counts from the more lowly and highly expressed alleles in each sample, we calculated AR values and used the provided adjusted p values to identify genes with significant Xi expression (Padj <0.05). We called a gene as "escape" if one or both of the datasets showed evidence of Xi expression.

The third dataset (Dataset 3 from Garieri et al.)[20] was derived from single-cell allelic expression in fibroblasts.[20] We included 203 genes that had data from at least two of the five samples in the dataset. We converted the reported values (Xa reads/total reads) to AR values using the following formula: $AR = \frac{1}{\frac{Xa\ reads}{total\ reads}} - 1$. We used the provided AR threshold to consider a gene significantly expressed from Xi in each sample (AR>0.0526). We called a gene as "escape" if it had at least one sample with significant expression from Xi.

The fourth dataset (Tables S4 and S5 from Sauteraud et al.)[21] was derived from allele-specific bulk RNA-seq performed on 136 LCLs with skewed X inactivation. We analyzed 215 genes that had data from at least 10 samples. We calculated an AR value for each gene in each sample using the read counts from the more lowly and highly expressed alleles in each sample, adjusting for the level of skewing in each sample. To identify genes that were significantly expressed from Xi across samples, we performed paired, two-sample, one-sided t-tests, asking whether the raw (pre-adjusted for skewing) AR values were greater than the baseline AR given the level of skewing in each sample ($baseline\ AR = \frac{1 - skewing\ coefficient}{skewing\ coefficient}$); we corrected the resulting p values for multiple comparisons with the p.adjust function in R using the Benjamini-Hochberg method. We considered genes with Padj <0.01 as significantly expressed from Xi ("escape").

The fifth dataset (Table S6 from San Roman et al.)[15] was derived from our prior analysis of allelic ratios in bulk RNA-sequencing of skewed LCLs and fibroblasts with two copies of the X chromosome. We calculated AR values for 152 genes in LCLs and 120 in fibroblasts. We used the Benjamini-Hochberg adjusted p values from one-sided, one-sample t-tests to determine whether AR values were significantly greater than zero, considering genes with Padj <0.05 as expressed from Xi ("escape").

Next, we synthesized the calls from these five datasets. We assigned a gene as having evidence for expression from Xi ("escape") if 1) most (>50% of) studies indicated "escape" or 2) 50% or fewer (but more than 0) studies indicated "escape" and either i) there was more than one study with evidence of escape or ii) the average AR across all studies was ≥ 0.1. We assigned genes as not expressed from Xi ("subject" to X chromosome inactivation) if: 1) all studies indicated "subject" or 2) most (>50%) studies indicated "subject" and the average AR across all studies was <0.1. For genes with no data from any of the five studies, we investigated whether there was evidence of expression from Xi using human-rodent hybrid cell lines carrying a human Xi from Carrel et al.[7] as compiled in Balaton et al.[22] If a gene was expressed in at least 22% of Xi hybrid cell lines (per Carrel et al.), we considered the gene to be expressed from Xi ("escape"). Our final annotations of "Xa-expressed" genes are listed in Table S4. In total, we found 107 genes with prior evidence of expression from Xi ("escape"), 460 genes without prior evidence of expression from Xi ("subject"—considered here as "Xa-expressed"), and 398 genes without data to make a call ("no call").

Analysis of Yq heterochromatin length

To assess Yq heterochromatin length, we analyzed whole genome sequencing data of 1,225 males from the 1000 Genomes Project.[26] We aligned FASTQ files with paired-end reads to the human reference genome (hg38) using bowtie2.[53] From each male in the sample, we calculated the average depth of coverage of the *DYZ1* region. In addition, we calculated the average depth of coverage of a 1-megabase single-copy region of the Y chromosome (from bases 14,500,000–15,500,000). We adjusted read depths to account for GC bias in sequencing depth.[60,61] In cases of individuals with multiple sequenced samples, we summed the read depths following GC-bias correction. For each male, we normalized mean *DYZ1* read depth by mean single-copy region read depth to account for differences in sequencing depth between different sequencing runs. After these steps, a higher depth of coverage implies higher *DYZ1* copy number and an increase in the size of the Yq heterochromatic region.

To analyze gene expression as a function of *DYZ1* read depth, we obtained RNA-seq data from The Geuvadis Consortium and restricted our analysis to samples marked as male, "UseThisDuplicate = = 1", RNA integrity number (RIN) $\geq$ 8, and with estimates for *DYZ1* read depth. We excluded the following outlier or incorrectly annotated samples as flagged in t'Hoen et al.[62]: NA12546.1, HG00329.5, NA18861.4, NA19144.4, NA19225.6, HG00237.4, NA12399.7, and NA07000.1, resulting in a total of 194 male samples. We downloaded the raw RNA-seq data and pseudoaligned the reads with kallisto as above. Using DESeq2, we modeled the $\log_2$ read counts as a function of $\log_2$ *DYZ1* depth of coverage, controlling for population and the lab that sequenced the libraries.

Analysis of gene expression in individuals with structural variants of the sex chromosomes

Naturally occurring structural variants of the sex chromosomes allowed us to investigate the consequences of deleting or duplicating specific genes on Chr X or Chr Y. The Page lab collected these samples over several decades during the course of studying infertility, disorders of sex development, and sex chromosome aneuploidy. We cultured these cells and performed RNA-sequencing in parallel with the sex chromosome aneuploidy cell lines.

Y isochromosomes

The Page lab previously identified three unrelated individuals with Y isochromosomes lacking the Yq heterochromatic region among males with spermatogenic failure.[63,64] One of these isochromosomes resulted from recombination between the IR4 repeats on Yp and Yq (WHT5557, referred to as a "pseudoisochromosome") and two resulted from recombination more distally, within the P1 palindrome. Both recombination events resulted in the duplication of protein-coding NPY genes and all PAR1 genes expressed in somatic cell types. For the 442 X- and Y-responsive autosomal genes in LCLs, we used PCA plots to visualize the clustering of the Y isochromosome samples within the Chr Y chromosome copy number series, controlling for a single X chromosome (0Y: 45,X; 1Y: 46,XY; 2Y: 47,XYY; 4Y: 49,XYYYY). For the PCA plots, we performed a variance stabilizing transformation on normalized counts from DESeq2 and used the removeBatchEffect function in the limma R package (v3.42.2)[65] to remove any effects of library preparation batch. Ellipses representing the 95% confidence intervals around the centroid of each group (with at least 3 samples) were computed using ClustVis software.[57]

X isochromosomes

Through karyotyping of individuals with Turner syndrome, we identified four unrelated individuals with X isochromosomes in which the entirety of Xq—and in some cases proximal portions of Xp—is duplicated. We mapped the breakpoints of these isochromosomes by fluorescent *in situ* hybridization (FISH) using bacterial artificial chromosomes (BACs) with known locations across the X chromosome as DNA probes (Figure S11; see below for FISH methods). In two individuals, WHT7183 and WHT7399, the breakpoints mapped to the centromere. In these two cases, probe 343B12, proximal to all genes on Xp, was absent on the isochromosome, and probe 168B01, proximal to all genes on Xq, was present in two copies. A third individual, WHT7182, had a breakpoint on Xp ~5 Mb from the centromere. This breakpoint mapped within the 206-kb region (chrX:52,607,059-52,813,686) covered by FISH probe 34G18. This region contains a palindrome (P5), the likely site of homologous recombination. A fourth individual, WHT7207, had a breakpoint on distal Xp near PAR1. We mapped the breakpoint to a 361-kb region (chrX:9,438,772-9,842,745) from *TBL1X* to *SHROOM2*. *TBL1X* is expressed at a level consistent with it being present on this individual's isochromosome, indicating that the breakpoint is proximal to *TBL1X*.

In analyzing gene expression from the X isochromosome samples, we discovered that the isochromosome was not always stable in the cell cultures. Samples with recombination in the centromere had stable karyotypes in both LCLs and fibroblasts. However, for the two samples with more distal Xp breakpoints, LCLs mostly or completely lost the isochromosome and thus could not be used in this analysis. Fibroblasts retained the isochromosome in enough cells that we could analyze their gene expression (WHT7182: 47% 46,X,i(Xq) and 53% 45,X, 49 interphase nuclei; WHT7207: 64% 46,X,i(Xq) and 36% 45,X, 100 interphase nuclei), however the resulting expression levels should be considered an average between these populations. For the X- and Y-responsive autosomal genes in LCLs or fibroblasts, we used PCA plots to visualize the clustering of the X isochromosome samples with the Chr X copy number series, without Y chromosomes (1X: 45,X; 2X: 46,XX; 3X: 47,XXX; 4X: 48,XXXX).

X-Y translocations

Among individuals with 46,XY disorders of sexual development or Turner syndrome, we identified three females (WHT1297, WHT1489, WHT1615) with X-Y translocations between the *PRKX* and *PRKY* genes [46,X,t(X; Y)(*PRKX-PRKY*)]. Recombination between *PRKX* and *PRKY* is common among 46,XY females and is facilitated by a polymorphic Yp inversion.[25,66] We used a PCR-based screening approach to determine and sequence the breakpoints (Table S7). These females are missing five NPX-NPY genes: *SRY*, *RPS4Y1*, *ZFY*, *AMELY*, *and TBL1Y*. Samples WHT1297 and WHT1489 are missing *PRKX* but retain *PRKY*, while sample WHT1615 is missing *PRKY* but retains *PRKX*.

Also among individuals with 46,XY disorders of sexual development, we ascertained four females (WHT1785, WHT1798, WHT1799, WHT2315) with X-Y translocations between the *ANOS1 (KAL1)* and *ANOS2P (KALP)* genes [46,X,t(X; Y)(*ANOS1-ANOS2P*)]. We identified breakpoints using a PCR-based screening approach (Table S7). In three of the cases (WHT1785, WHT1798, and WHT1799, *ANOS1* is retained, while *ANOS2P* is deleted. In WHT2315, the recombination is within *ANOS1* and *ANOS2P*, resulting in the partial deletion of each. Based on RNA-seq data of the translocated samples, some or all of the of the genes on Yq are not expressed. This is likely due to spreading of X chromosome inactivation onto the translocated portion of the Y. In WHT1799, no Y genes are expressed; in WHT1785 and WHT1798 only *EIF1AY* is expressed; while in WHT2315 *TXLNGY* and *KDM5D* are expressed at low levels and *EIF1AY* is expressed (Table S7).

For the X- and Y-responsive autosomal genes in LCLs, we used PCA plots to visualize the clustering of the X-Y translocation samples with the Chr X and Y copy number series (1X: 45,X; 2X: 46,XX; 2X: 47,XXX; 4X: 48,XXXX; 1Y: 46,XY; 2Y: 47,XYY; 4Y: 49,XYYYY).

Fluorescent *in situ* hybridization

To assess copy number and variations in X and Y chromosome structure, we performed DNA fluorescent *in situ* hybridization using BACs with known locations as DNA probes, as previously described.[63] We labeled FISH probes by nick translation with biotin-dUTP (Sigma) and Cy3-dUTP (Amersham). To detect Biotin-labelled probes, we used Avidin-FITC probes (Life Technologies, 1:250 dilution), and counterstained DNA with DAPI. We captured images on a DeltaVision deconvolution microscope. We provide information on probes for FISH experiments in Table S7. For copy number analysis, we performed FISH on interphase cells using Whole X and Whole Y probes (Applied Spectral Imaging).

Motif analysis

We analyzed DNA motif enrichment using Homer software v4.11.1.[56] The findMotifs function identified *de novo* motifs enriched in promoters of a gene set of interest (e.g., Chr X-responsive autosomal genes in LCLs) relative to expressed genes as a "background" set (e.g., autosomal genes expressed in LCLs). We defined promoters as +/− 1 kb from the transcription start site. We used the "-fdr" argument with 100 randomizations to calculate an empirical FDR for the motif enrichments. We then compared the enriched motifs to motif databases to identify the best match among known DNA-binding proteins.

Analysis of ZFX ChIP-seq data

To detect ZFX binding at gene promoters, we analyzed four ZFX ChIP-seq experiments in different cell lines from ENCODE4 v1.6.1 (HCT 116, C4-2B, MCF-7, and HEK-293T). Using the BEDTools closest function, we mapped ZFX peaks to the nearest annotated transcription start site in the GENCODE v24 comprehensive annotation.[55] For gene expression analysis in each cell line, we processed fastq files from ENCODE or GEO[31,32] using kallisto as above or, when available, we downloaded pre-processed RNA-seq data. We restricted our analysis of ZFX peaks in each cell line to genes with median TPM $\geq$ 1.

			Accession #		
ChIP-seq target	Cell line	Karyotype	IDR-thresholded narrow peak bed file	Fold change versus control bigWig file	RNAseq files
ZFX	HCT 116	46,XY	ENCFF858YWR	ENCFF102UZB	TPMs: ENCFF435PHM
	C4-2B	46,XY	ENCFF160AXQ	ENCFF264NUJ	fastq: GSE102616
	MCF-7	46,XX	ENCFF861DOL	ENCFF429AXS	TPMs: ENCFF721BRA<br>fastq: GSE102616
	HEK-293T	46,XX	ENCFF292HYE	ENCFF625XHP	fastq: GSE145160

To evaluate ZFX binding in promoters of genes, we investigated whether there was a ChIP-seq peak 1 kb up- or downstream of the transcription start site. To get a sense for the most robust binding sites across cell types, we calculated the proportion of cell lines bound at the promoter of each gene by dividing the number of cell lines with binding by the number of cell lines in which that gene is expressed.

Identification of ZFX direct target genes from published data

To determine the direct targets of ZFX, we combined ZFX ChIP-seq results (analyzed as described above) and gene expression analysis of *ZFX* knockdown or knockout from Rhie et al. and Ni et al. in three cell lines: MCF7, C4-2B, and HEK293T.[31,32] Direct target genes in each cell line were defined as genes where ZFX bound within 1 kb of the transcription start site and that significantly change in expression with *ZFX* knockdown or knockout. We obtained the raw RNA-sequencing data from GEO and processed the RNA-seq data as above: pseudoaligning and quantifying transcript abundances with kallisto and performing differential expression analysis (*ZFX* knockdown or knockout versus control) using DESeq2. We considered genes with Padj<0.05 to be significantly ZFX-responsive.

CRISPR interference

To knock down endogenous *ZFX* or *ZFY* levels in 46,XX and 46,XY fibroblasts, we used CRISPR interference to target a nuclease-dead Cas9 fused with a repressive KRAB domain (dCas9-KRAB) to the *ZFX* or *ZFY* promoter and thereby repress *ZFX* or *ZFY* transcription.

Lentiviral production

We obtained the following constructs from Addgene for use in our experiments: 1) for CRISPRi, pLX_311-KRAB-dCas9 (Addgene: #96918), a KRAB-dCas9 blasticidin-selectable lentiviral expression vector[38]; 2) sgOpti (#85681), a puromycin-selectable structurally optimized guide RNA (gRNA) lentiviral expression vector[67,68]; 3) psPAX2 (#12260), a second generation lentiviral packaging plasmid; and 4) pCMV-VSV-G (#8454), a lentiviral envelope protein plasmid.[69] We purified all plasmids using the EndoFree Maxi Kit (Qiagen).

We used the top five guide sequences for *ZFX* and *ZFY* from the human CRISPRi v2 gRNA library.[50] We tested these guides for *ZFX* or *ZFY* knockdown and chose the two guides that gave the most robust response for the final experiments. We also used intragenic (IG) control guides mapping to a gene-poor region on Chr 2.[51] Guides and primer sequences are listed below.

Guide name	Protospacer sequence	Primer, forward	Primer, reverse
ZFX-2	GGGCGGCACCCGGGACTCAC	CACCGGGCGGCACCCGGGACTCAC	AAACGTGAGTCCCGGGTGCCGCCC
ZFX-3	GGCACCCGGGACTCACCGGA	CACCGGCACCCGGGACTCACCGGA	AAACTCCGGTGAGTCCCGGGTGCC
ZFY-1	GGCACGCGGGACTCACCCGA	CACCGGCACGCGGGACTCACCCGA	AAACTCGGGTGAGTCCCGCGTGCC
ZFY-2	GTGCGGGCGGTCGGCGACAG	CACCGTGCGGGCGGTCGGCGACAG	AAACCTGTCGCCGACCGCCCGCAC
IG-1	GACATATAAGAGGTTCCCCG	CACCGACATATAAGAGGTTCCCCG	AAACCGGGGAACCTCTTATATGTC
IG-3	ACCACACGGAGTTACCATGG	CACCACCACACGGAGTTACCATGG	AAACCCATGGTAACTCCGTGTGGT

To clone guides into the sgOpti vector, we digested using FastDigest BsmBI (ThermoFisher) and dephosphorylated the ends with FastAP (ThermoFisher) for 30 min at 37°C. We gel-purified the digested plasmid using the QIAquick Gel Extraction Kit (Qiagen). Prior to ligation, we phosphorylated and annealed each pair of oligos using T4 Polynucleotide kinase (New England BioLabs). We then ligated each insert into the backbone using Quick Ligase (New England BioLabs) for 10 min at room temperature, and transformed into NEB Stable Cells for amplification (New England BioLabs). We confirmed gRNA sequences by sequencing.

LentiX-293T cells (Takara) were cultured in DMEM with 2mM L-Glutamine, 100U/mL Penicillin-Streptomycin, and 10% FBS on plates coated with collagen (5 μg/cm^2) to produce virus. For gRNA constructs, we plated 5x10^6 LentiX-293T cells in one 10 cm plate the night before transfection. The next day, we co-transfected 4.5 μg of sgOpti, 4 μg of pCMV-VSV-G, and 6.5 μg of psPAX2 using TransIT-LT1 reagent (Mirus). We harvested virus-containing media once at 48 h and once at 72 h, pooled, and tested for successful viral production using Lenti-X GoStiX (Takara). We concentrated gRNA virus 10X using Lenti-X concentrator (Takara). For the dCas9-KRAB, we co-transfected five plates of LentiX-293T cells with 7 μg of pLX_311, 3 μg of pCMV-VSV-G, and 5 μg of psPAX2. We added Viral Boost reagent (Takara) 24 h post transfection, and again with replacement media after harvesting at 48 h. We pooled virus-containing media across all plates and concentrated 100X.

Generation of dCas9-KRAB fibroblast cultures

For CRISPRi we transduced 46,XX and 46,XY fibroblasts from three individuals each. We performed transductions using the "spin-fection" method: we added 1 mL fibroblast media with 5–20 μL of 100X concentrated pLX_311 lentiviral vector and 8 μg/mL polybrene (hexamethrine bromide; Sigma) to 7.5x10^4 cells per well in 12-well plates or 1.875x10^5 cells per well in 6-well plates, spun at 1000 rpm for 1 h and incubated overnight. 24 h post infection, we trypsinized each and expanded to 6-well or 10-cm plates with fibroblast media containing 5 μg/mL blasticidin (Life Technologies), which we maintained throughout the experiment for continuous selection.

ZFX and ZFY knockdown and analysis

We transduced control (intragenic) and *ZFX*- or *ZFY*-targeting gRNAs into the stably-expressing dCas9-KRAB fibroblasts as above, and selected cells using 2 μg/mL puromycin (Sigma) beginning 24 h post infection. We collected cells 72 h post infection. We isolated RNA and prepared libraries for RNA-seq as described above and sequenced using 150 bp paired-end reads on a NovaSeq 6000. We trimmed reads to 100 bp, pseudoaligned with kallisto, and imported the count data into R using tximport, as described above. We used DESeq2 to perform differential expression analysis including sgRNA target (*ZFX*, *ZFY*, or intergenic control) and cell line as covariates.

QUANTIFICATION AND STATISTICAL ANALYSIS

We used various statistical tests to calculate p values as indicated in the STAR Methods section, figure legend, or text, where appropriate. To calculate all statistics and generate plots, we used R software, version 3.6.3.[70] We considered individual results statistically significant when $p < 0.05$ or, when using multiple hypothesis correction, $P_{adj} < 0.05$ or FDR<0.05. In cases where conclusions were based on multiple statistical analyses, we corrected the p values using the Bonferroni method. The minimum reportable number in R software is 2.2e−308, so p values close to zero were reported as less than this value. Unless otherwise stated, we plotted data summaries as median and interquartile range. We used Deming regressions to compare variables that are measured with error (e.g., $\log_2$ fold change per chrX vs. $\log_2$ fold change per chrY). We calculated Deming regressions using the R package "deming" v1.4. For weighted Deming regressions, we included error values using the "xstd" and "ystd" arguments.

Cell Genomics, Volume *4*

Supplemental information

The human Y and inactive X chromosomes similarly modulate autosomal gene expression

Adrianna K. San Roman, Helen Skaletsky, Alexander K. Godfrey, Neha V. Bokil, Levi Teitz, Isani Singh, Laura V. Blanton, Daniel W. Bellott, Tatyana Pyntikova, Julian Lange, Natalia Koutseva, Jennifer F. Hughes, Laura Brown, Sidaly Phou, Ashley Buscetta, Paul Kruszka, Nicole Banks, Amalia Dutra, Evgenia Pak, Patricia C. Lasutschinkow, Colleen Keen, Shanlee M. Davis, Angela E. Lin, Nicole R. Tartaglia, Carole Samango-Sprouse, Maximilian Muenke, and David C. Page

Figure S1

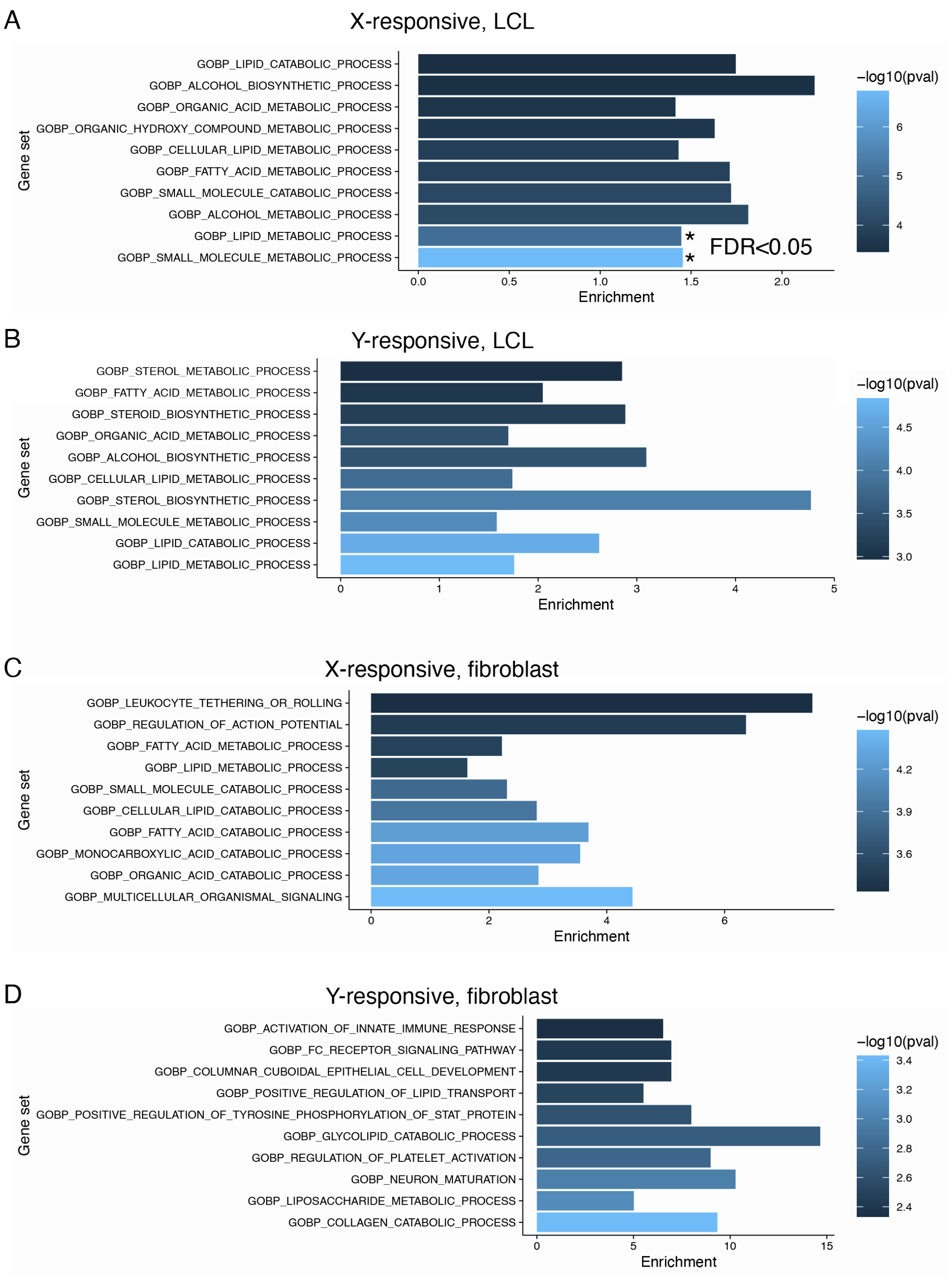

Figure S1. Analysis of gene ontology category enrichment in autosomal genes that significantly respond to Chr X or Y copy number, related to Figure 1. Enrichment of

“Biological Process” Gene Ontology categories was calculated separately for autosomal genes that significantly responded to Chr X or Y copy number in LCLs or fibroblasts using Fisher’s exact test. All expressed genes in LCLs or fibroblasts were used as the background set. Shown here are the top 10 enriched categories based on P value. Stars indicate those that were below the Benjamini-Hochberg-corrected P value (FDR) of 0.05. There were no significant categories (by FDR threshold) for genes that responded to Chr Y copy number in LCLs, nor for genes that responded to Chr X or Y copy number in fibroblasts.

Figure S2

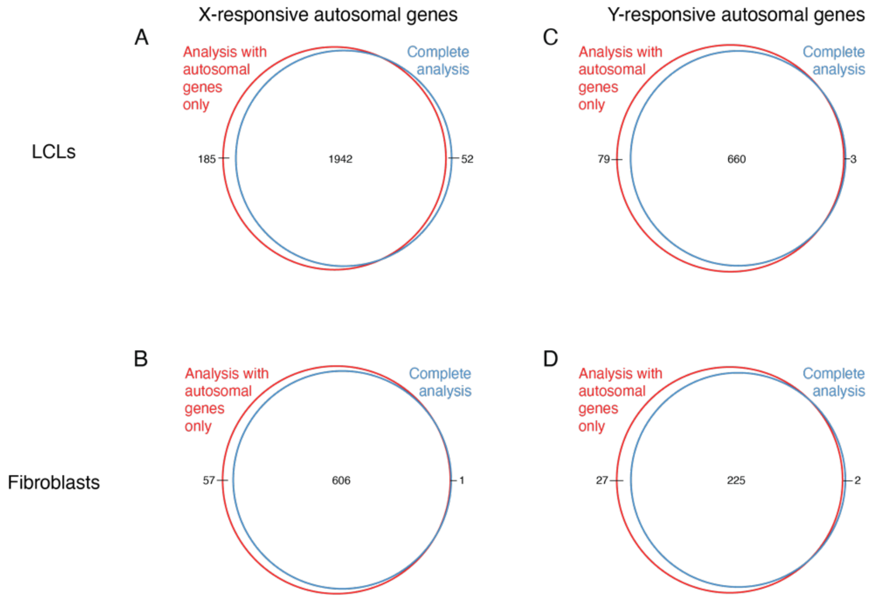

Figure S2. Mapping to a transcriptome with or without the sex chromosomes does not affect X- and Y-responsive autosomal genes in LCLs and fibroblasts, related to Figure 1. Read depth normalization between samples and differential expression was performed using an annotation only containing autosomal genes to rule out artifacts of bulk over-expression of sex chromosome genes, and compared to the analysis conducted using the complete annotation. Significantly X-responsive genes in LCLs (A) and fibroblasts (B) were almost completely overlapping between the two analyses, as were the significantly Y-responsive genes in LCLs (C) and fibroblasts (D).

Figure S3

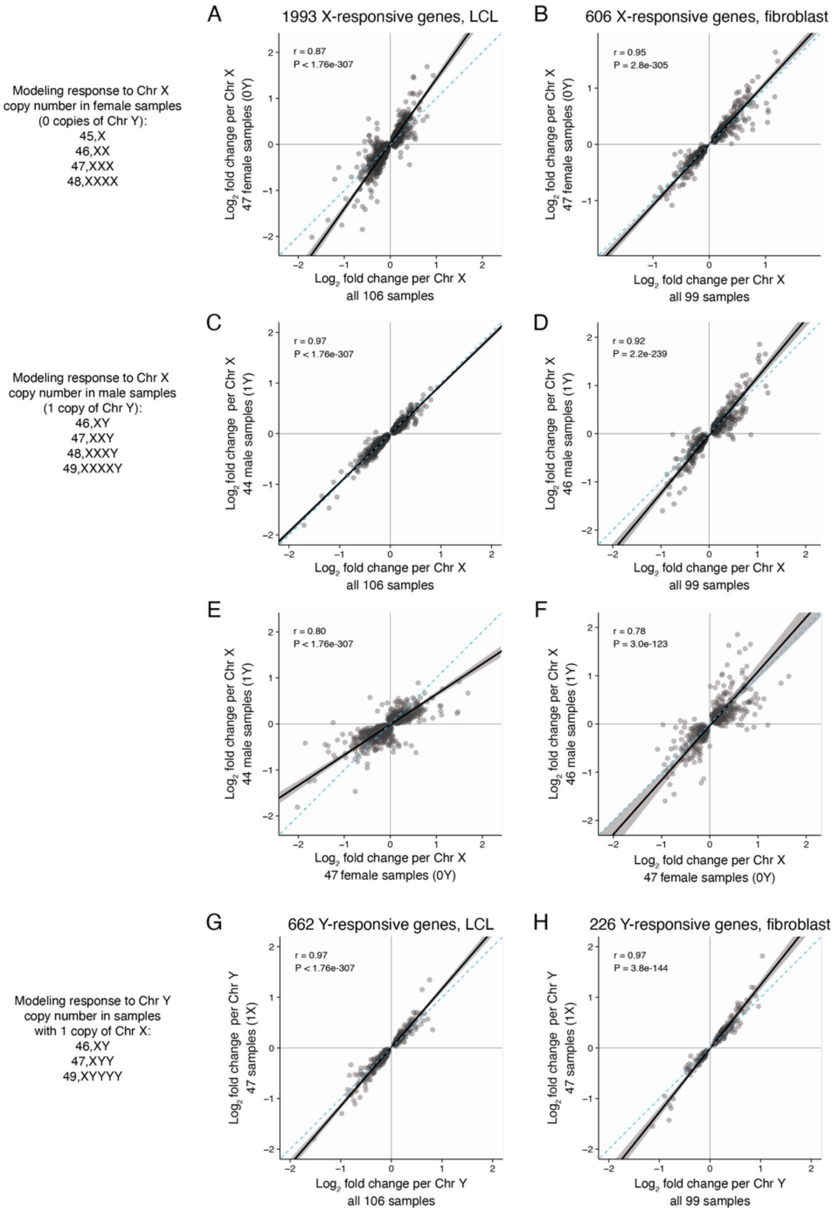

Figure S3. Modeling Chr X or Y copy number separately is highly correlated with models including both covariates, related to Figure 1. (A-D) Scatterplots comparing models of gene expression as a function of Chr X copy number in female samples (zero Y chromosomes; [A, B]) or male samples with one Y chromosome (C, D) with the full model show high correlations. **(E, F)** Scatterplots comparing models of gene expression as a function of Chr X copy number in male samples with one Y chromosome and female samples (no Y chromosomes) show high

correlations. **(G, H)** Scatterplots comparing a model of gene expression as a function of Chr Y copy number in male samples with one X chromosome with the full model reveal a high correlation. Black line and grey shading, Deming regression and 95% confidence interval; blue dashed line, identity (X=Y) line. Pearson correlation coefficients and Bonferroni-adjusted P values are indicated.

Figure S4

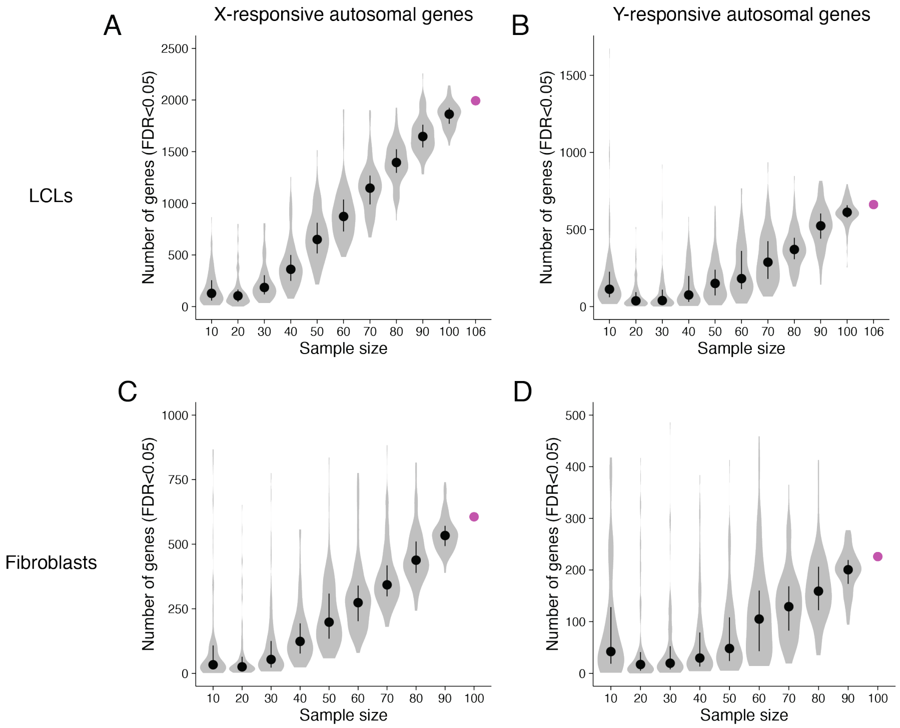

Figure S4. Detection of autosomal genes significantly responsive to Chr X and Y copy number is not saturated at the current sample size, related to Figure 1. To determine whether we had saturated the signal of significantly Chr X- or Y-responsive genes at the given sample size, we performed a bootstrapping analysis. Violin plots with median (dots) and interquartile range (whiskers) showing the number of Chr X-responsive (**A, C**) or Y-responsive (**B, D**) autosomal genes detected in random subsets of the LCL (A,B) or fibroblast (C,D) RNA-seq libraries. The number of samples in each subset is shown on the x-axis. 100 random samples were obtained at each sample size. Pink dots show the number of Chr X- and Y-responsive genes detected in the full set of samples. The large number of genes detected in some of the smallest subsets is caused by unbalanced karyotype distributions.

Figure S5

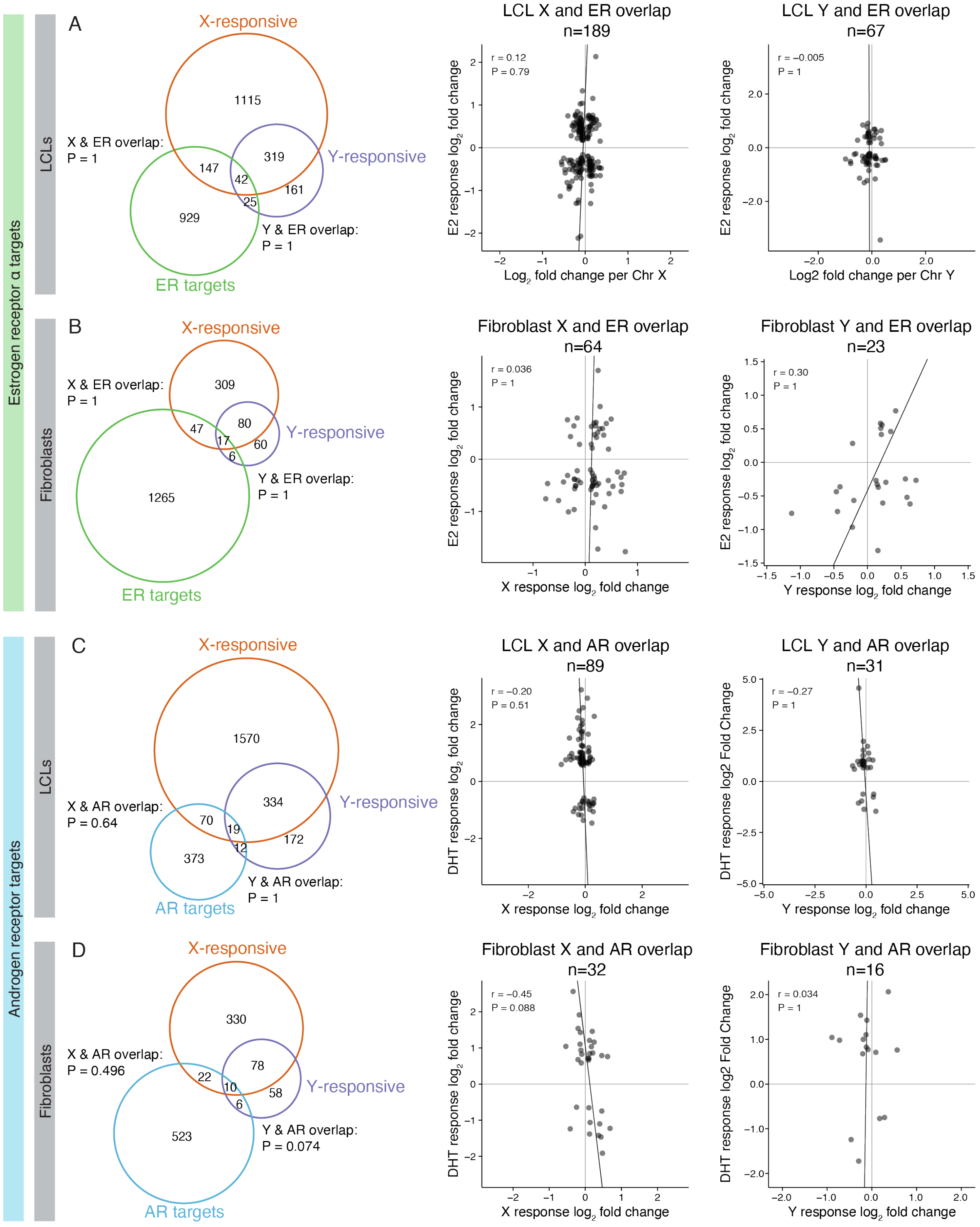

Figure S5. The response to Chr X- and Y-responsive genes is distinct from the response to sex hormones, as defined by estrogen receptor alpha and androgen receptor direct target

genes, related to Figure 1. (A-B) *Left,* Venn diagrams showing overlap of ERα direct target genes (bound by ERα within 30 kb of the transcription start site and differentially expressed [P_{adj}<0.05] with 17-ß estradiol [E2] treatment) and Chr X- or Y-responsive genes in LCLs (A) or fibroblasts (B). Venn diagrams were restricted to autosomal genes expressed in both MCF7 cells (where ERα direct targets were defined) and LCLs or fibroblasts. *Right,* scatterplots of overlapping genes showing the relationship between response to E2 and Chr X or Y copy number. **(C-D)** *Left,* Venn diagrams showing overlap of AR direct target genes (bound by AR within 30 kb of the transcription start site and differentially expressed [P_{adj}<0.05] with dihydrotestosterone [DHT] treatment) and Chr X- or Y-responsive genes in LCLs (C) or fibroblasts (D). Venn diagrams were restricted to autosomal genes expressed in both LNCaP cells (where AR direct targets were defined) and LCLs or fibroblasts. *Right,* scatterplots of overlapping genes showing the relationship between response to DHT and Chr X or Y copy number. Venn diagram Bonferroni-adjusted P values, hypergeometric test; scatterplots show Deming regressions (black lines), Pearson correlation coefficients and Bonferroni-adjusted P values.

Figure S6

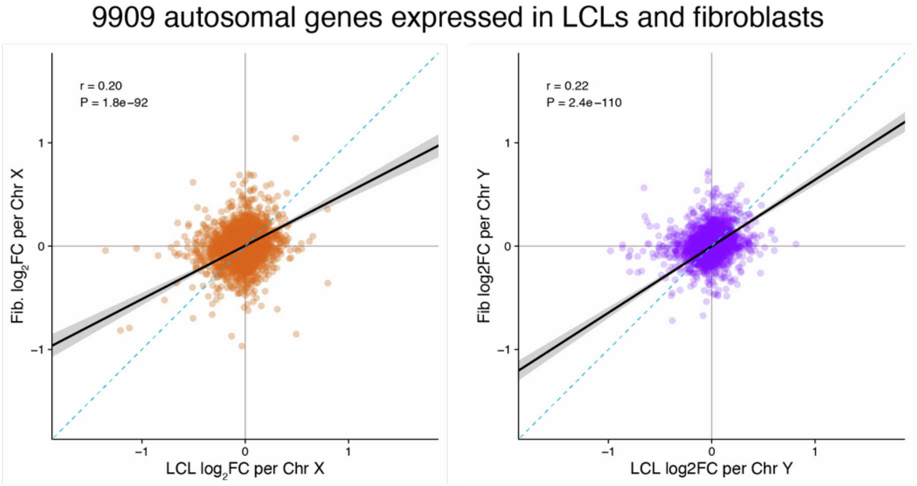

Figure S6. Response to Chr X or Y copy number is weakly correlated between cell types, related to Figure 1. Scatterplots comparing the effect sizes of the response to Chr X (left) or Y (right) copy number in LCLs versus fibroblasts for all autosomal genes expressed in both cell types. Black line and grey shading, weighted Deming regression and 95% confidence interval; blue dashed line, identity (X=Y) line; Pearson correlation coefficients and Bonferroni-adjusted P values are indicated.

Figure S7

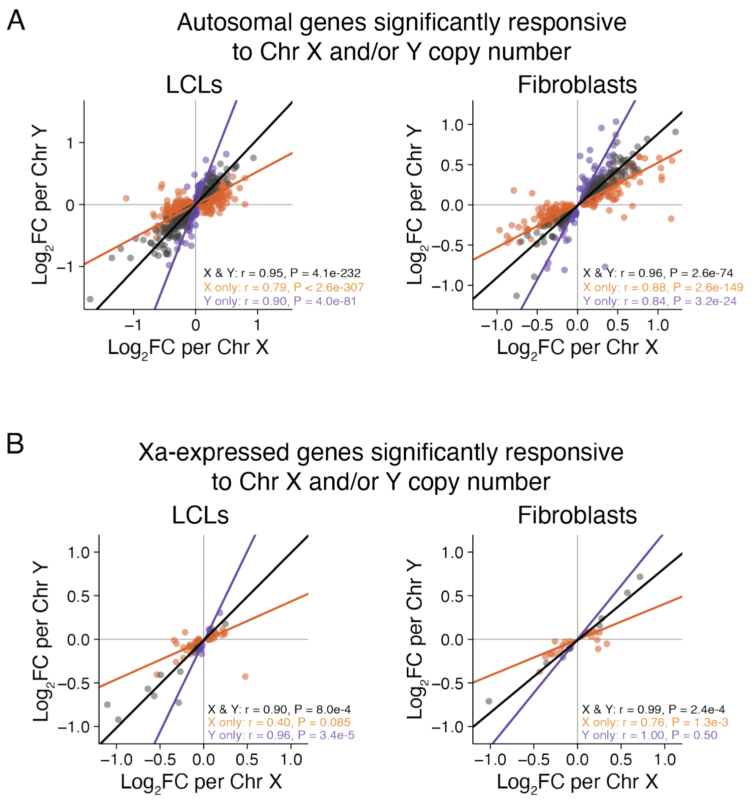

Figure S7. Correlation plots for significantly X and/or Y responsive genes, related to Figure 2. Scatterplots showing the relationship between the effects per additional copy of Chr X or Y for autosomal genes **(A)** or Xa-expressed genes **(B)** called as significantly responsive (P_{adj}<0.05) to either Chr X, Chr Y, or both. Weighted Deming regression lines, Pearson correlation coefficients, and Bonferroni-adjusted P values of the correlation are included in each plot.

Figure S8

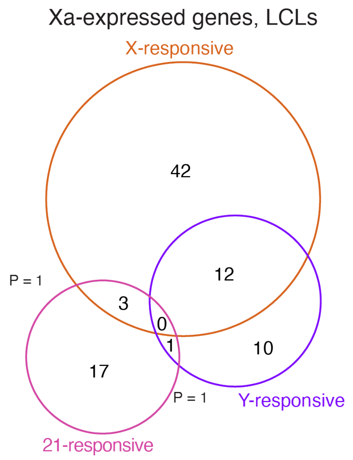

Figure S8. For Xa-expressed genes, the response to Chr 21 copy number is distinct from the shared response to Chr X and Chr Y copy number, related to Figure 3. Venn diagram of Xa-expressed genes that significantly respond to Chr 21, Chr X, or Chr Y copy number. This analysis is limited to genes previously annotated as transcribed from Xa but not from Xi. Bonferroni-adjusted P values, hypergeometric test.

Figure S9

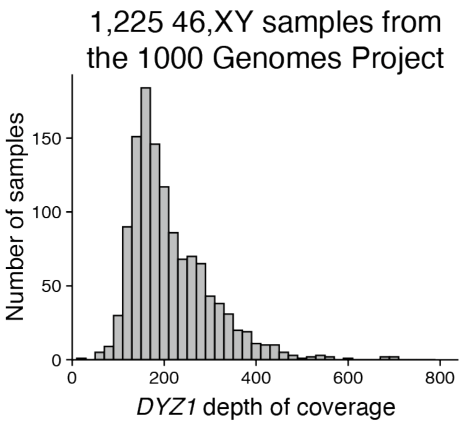

Figure S9. Wide range of Y heterochromatin lengths in males from the 1000 Genomes Project, related to Figure 4. Histogram of *DYZ1* depth of coverage – a proxy for Chr Y heterochromatin length – for 1,225 46,XY samples from the 1000 Genomes Project.

Figure S10

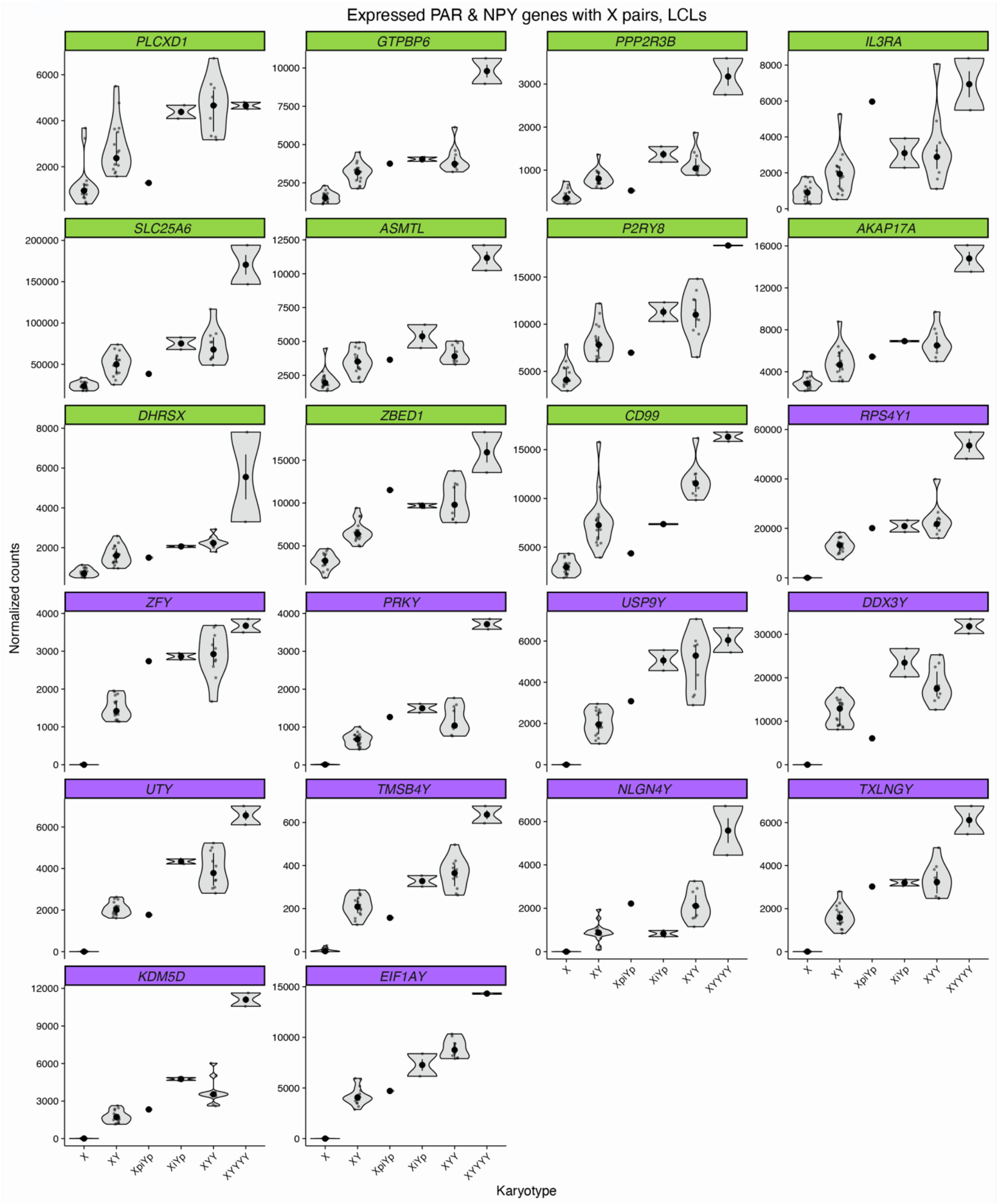

Expressed PAR & NPY genes with X pairs, LCLs
PLCXD1
GTPBP6
PPP2R3B
IL3RA
SLC25A6
ASMTL
P2RY8
AKAP17A
DHRSX
ZBED1
CD99
RPS4Y1
ZFY
PRKY
USP9Y
DDX3Y
UTY
TMSB4Y
NLGN4Y
TXLNGY
KDM5D
EIF1AY
Normalized counts
X
XY
XpiYp
XiYp
XYY
XYYYY
Karyotype

Figure S10. Chr Y gene expression in samples with variant Y chromosomes, related to Figure 4. Violin plots showing median and interquartile range for all expressed Chr Y genes (PAR1 and NPY) for samples with two types of variant Y chromosomes: isoYp (XiYp) with recombination in palindrome P1, pseudoisoYp (XpiYp) with recombination between IR4 inverted repeats, or samples with zero to four copies of Chr Y and one copy of Chr X. Green, PAR1 genes; purple, NPY genes with homologs on Chr X.

Figure S11

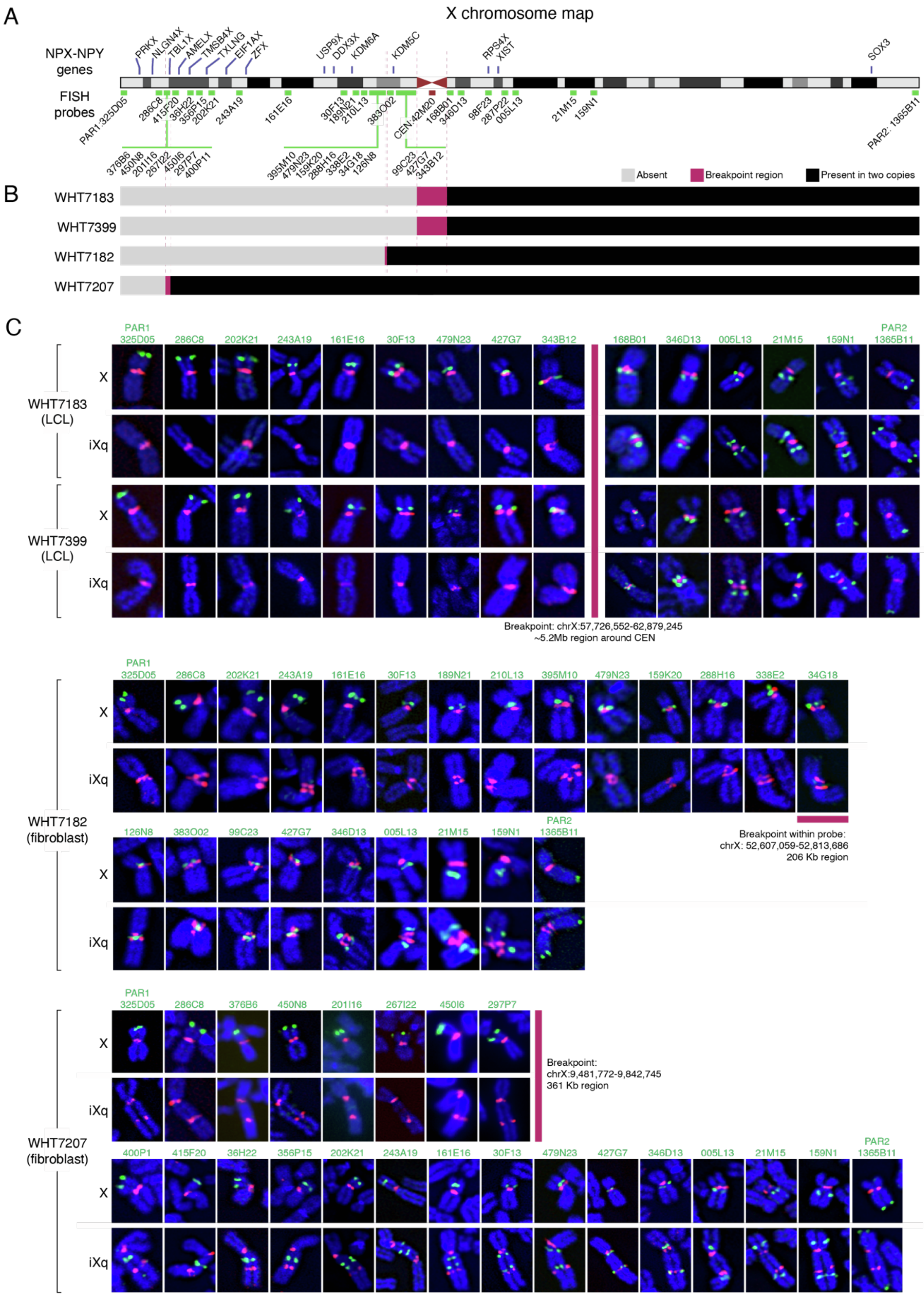

A
X chromosome map
NPX-NPY genes
FISH probes
Absent
Breakpoint region
Present in two copies
B
WHT7183
WHT7399
WHT7182
WHT7207
C
WHT7183 (LCL)
WHT7399 (LCL)
Breakpoint: chrX:57,726,552-62,879,245
~5.2Mb region around CEN
WHT7182 (fibroblast)
Breakpoint within probe:
chrX: 52,607,059-52,813,686
206 Kb region
WHT7207 (fibroblast)
Breakpoint:
chrX:9,481,772-9,842,745
361 Kb region

Figure S11. Determination of X isochromosome breakpoints in four individuals using DNA FISH, related to Figure 5. (A) Chr X ideogram showing locations of NPX-NPY gene pairs and FISH probes. Centromere probe 42M20 (labeled in red) served as a point of reference in all experiments; all other probes labeled in green. **(B)** Locations of breakpoints on X isochromosomes as determined by FISH. Locations of breakpoints correspond to schematic of X Chr in (A) and are indicated with pink shading. Grey, region absent in iXq; pink, breakpoint region; black, region present in two copies in iXq. **(C)** Breakpoints for iXq chromosomes were refined using DNA FISH. For each individual and probe, an image of a normal Chr X and iXq are included from the same chromosome spread for comparison. Images for WHT7183 and WHT7399 were obtained from FISH on spreads from LCLs, while those for WHT7182 and WHT7202 were obtained from FISH on spreads from fibroblasts. See Table S7 for additional details of gene content and FISH probes.

Figure S12

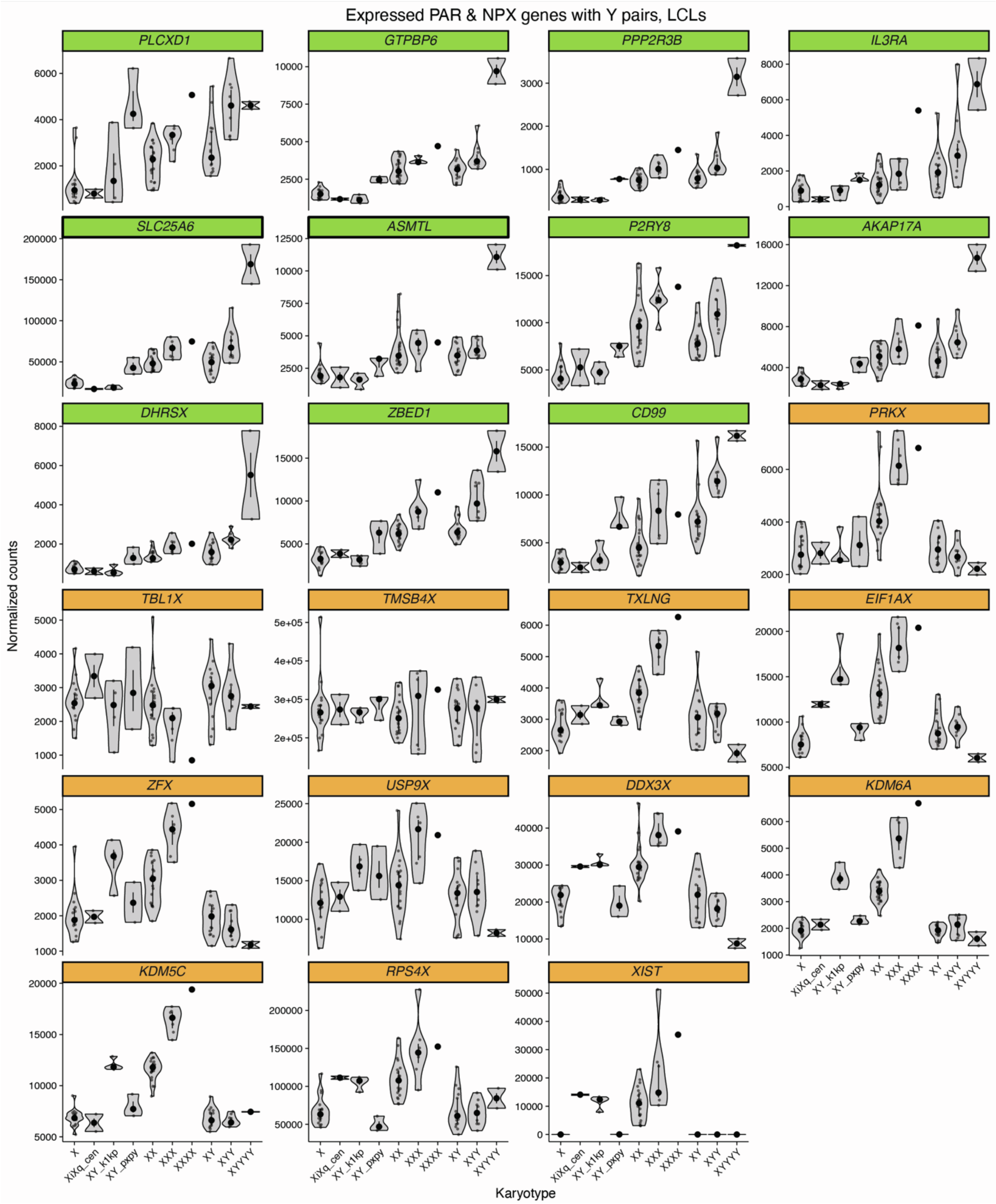
Expressed PAR & NPX genes with Y pairs, LCLs
PLCXD1
GTPBP6
PPP2R3B
IL3RA
SLC25A6
ASMTL
P2RY8
AKAP17A
DHRSX
ZBED1
CD99
PRKX
TBL1X
TMSB4X
TXLNG
EIF1AX
ZFX
USP9X
DDX3X
KDM6A
KDM5C
RPS4X
XIST
Normalized counts
X
XiXq_cen
XY_k1kp
XY_pxpy
XX
XXX
XXXX
XY
XYY
XYYYY
Karyotype

Figure S12. Expression levels of PAR1 and NPX genes with NPY homologs in LCL samples with structural variants of Chr X or Chr Y, related to Figure 5. Violin plots showing median and interquartile range for expressed PAR1 genes (green) and NPX genes with NPY homologs and *XIST* (orange) for samples with Chr X or Y structural variants or samples with one to four copies of Chr X and one to four copies of Chr Y. Karyotypes with only one sample are represented as a single dot. XiXq_cen: 46,X,i(Xq) samples with recombination in the centromere; XY_k1kp: 46,X,t(X;Y)(*ANOS1-ANOS2P*); XY_pxpy: 46,X,t(X;Y)(*PRKX-PRKY*).

Figure S13

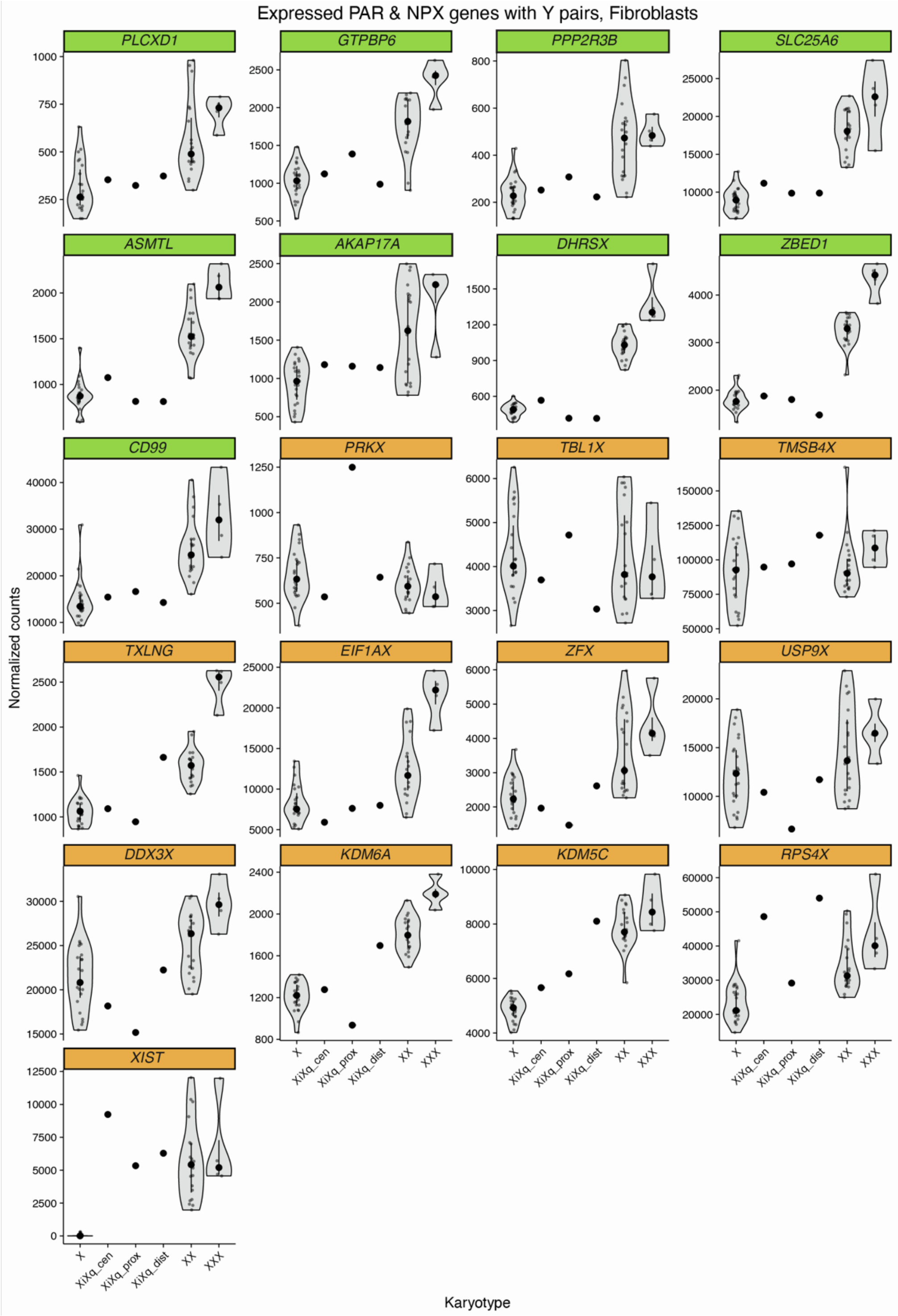

Expressed PAR & NPX genes with Y pairs, Fibroblasts
PLCXD1
GTPBP6
PPP2R3B
SLC25A6
ASMTL
AKAP17A
DHRSX
ZBED1
CD99
PRKX
TBL1X
TMSB4X
TXLNG
EIF1AX
ZFX
USP9X
DDX3X
KDM6A
KDM5C
RPS4X
XIST
Normalized counts
X
XiXq_cen
XiXq_prox
XiXq_dist
XX
XXX
Karyotype

Figure S13. Expression levels of PAR1 and NPX genes with NPY homologs in fibroblast samples with structural variants of Chr X, related to Figure 5. Violin plots showing median and interquartile range for expressed PAR1 genes (green) and NPX genes with NPY homologs and *XIST* (orange) for samples with Chr X structural variants or samples with one to three copies of Chr X. Karyotypes with only one sample are represented as a single dot. XiXq_cen: 46,X,i(Xq) samples with recombination in the centromere; XiXq_prox: 46,X,i(Xq) samples with recombination in proximal Xp; XiXq_dist: 46,Xi(Xq) samples with recombination in distal Xp.

Figure S14

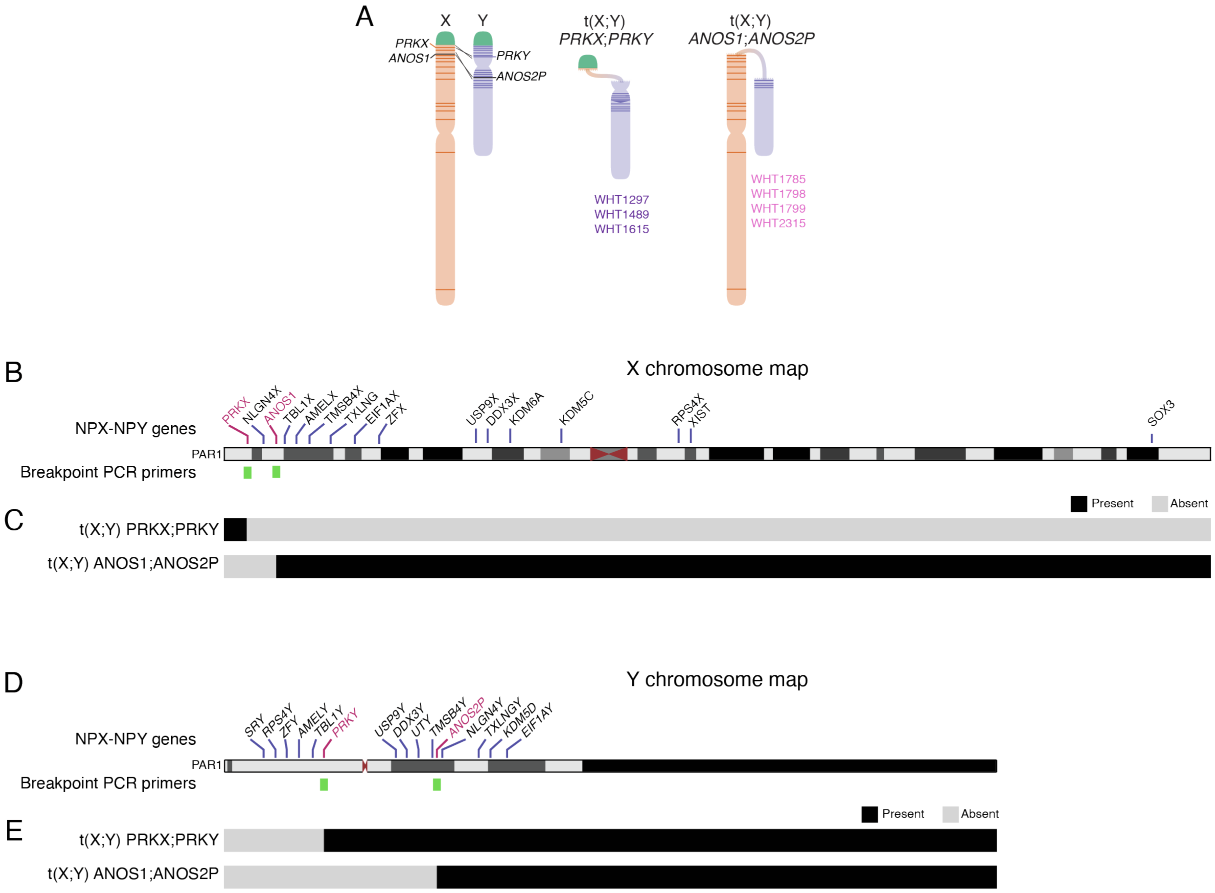

Figure S14. X-Y translocation breakpoints in seven individuals, related to Figure 5. (A) Schematic showing recombination between X and Y Chrs at *PRKX* and *PRKY*, or at *ANOS1* and *ANOS2P*, resulting in X-Y translocation products. **(B,D)** Chr X (B) and Chr Y (D) ideograms showing locations of NPX-NPY gene pairs, *ANOS1,* and *ANOS2P*. The locations of primers to amplify the breakpoints are indicated; for more details see Table S7. **(C,E)** Locations of breakpoints on the X (C) and Y (E) chromosomes as determined by PCR. Locations of breakpoints correspond to schematic of corresponding chromosomes above. Grey, region absent in t(X;Y) chromosome; black, region present in t(X;Y) chromosome.

Figure S15

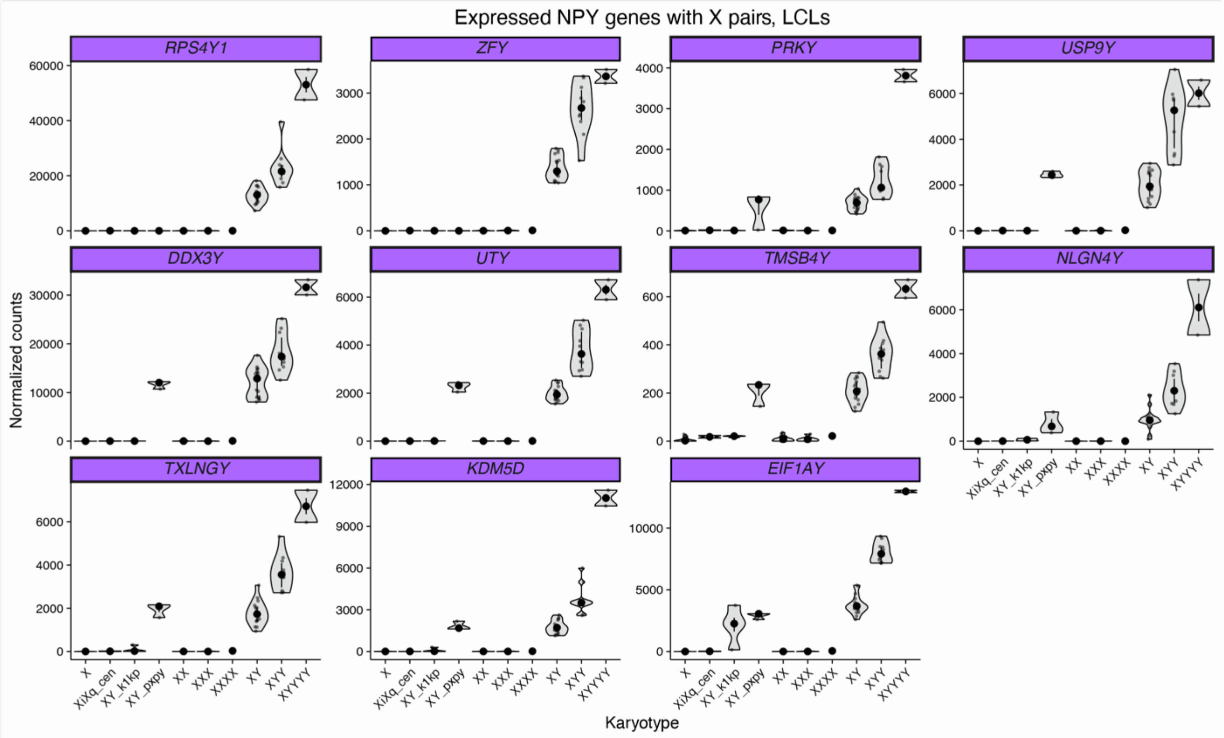

Figure S15. Expression levels of NPY genes with NPX homologs in LCL samples with structural variants of Chr X or Chr Y, related to Figure 5. Violin plots showing median and interquartile range for expressed NPY genes for samples with Chr X or Y structural variants or samples with one to four copies of Chr X and one to four copies of Chr Y. Karyotypes with only one sample are represented as a single dot. XiXq_cen: 46,X,i(Xq) samples with recombination in the centromere; XY_k1kp: 46,X,t(X;Y)(*ANOS1*-*ANOS2P*); XY_pxpy: 46,X,t(X;Y)(*PRKX*-*PRKY*).

Figure S16

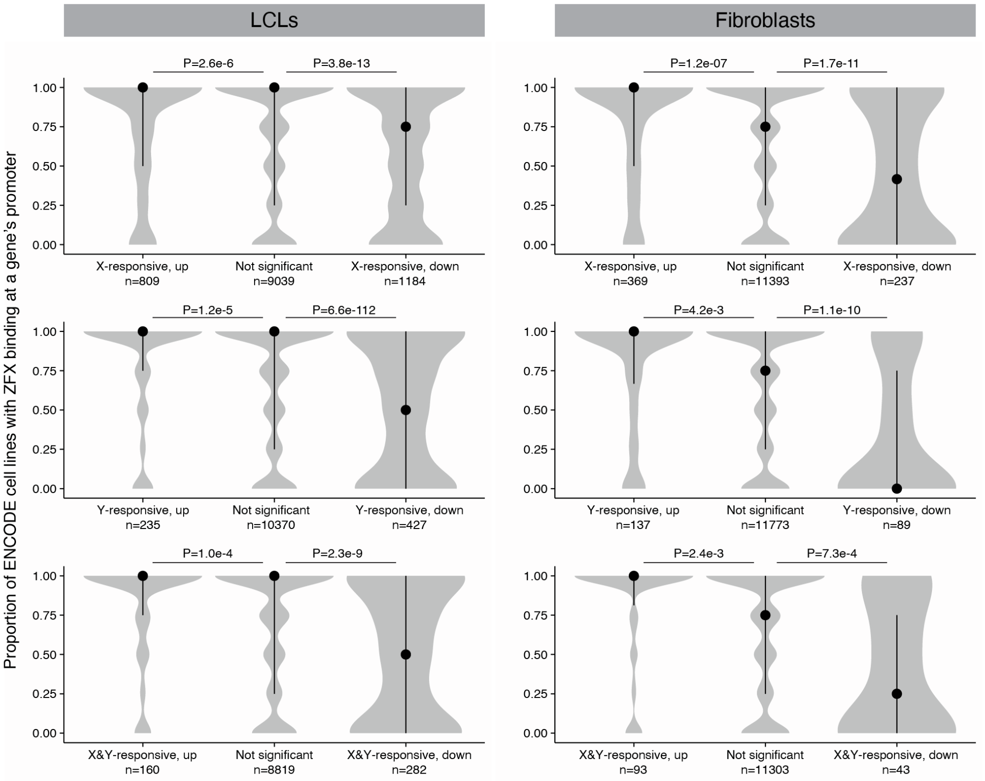

Figure S16. ZFX protein binding is enriched in promoters of autosomal genes whose expression increases in response to Chr X and/or Y copy number, and is depleted in promoters of genes whose expression decreases, related to Figure 6. For autosomal genes with or without significant expression changes with Chr X or Chr Y copy number, we investigated ZFX binding using publicly available ChIP-seq data from four cell lines. Violin plots, median (dot), and interquartile range (line) of the proportion of cell lines with ZFX binding at the gene's promoter (in cell lines where the gene is expressed). For example, a gene expressed in three of the four cell types with a ZFX peak in the promoter in those three cell types would have a proportion bound of 1. Top row, Chr X-responsive autosomal genes; middle row, Chr Y-responsive autosomal genes; bottom row, Chr X- and Y-responsive autosomal genes. The number of genes in each violin is indicated. Bonferroni-adjusted P values, Wilcoxon rank sum test.

Figure S17

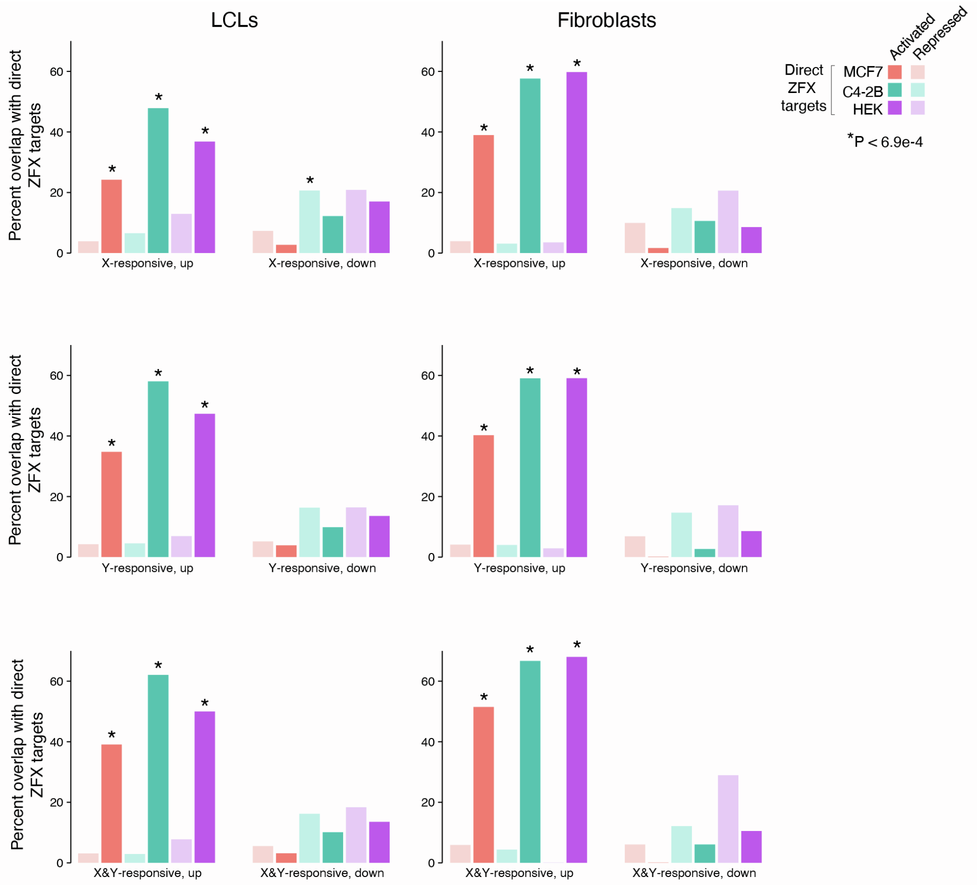

Figure S17. Published ZFX-activated genes are enriched in autosomal genes positively responsive to Chr X and Y copy number, related to Figure 6. Bar plots showing the percentage of genes responsive to Chr X and/or Y copy number that were identified as ZFX direct target genes in three cell types: MCF7, C4-2B, and HEK-293T [S1,2]. We defined direct target genes as those having a ZFX binding peak within 1 kb of the transcription start site and displaying significant expression changes (FDR<0.05) upon *ZFX* knockdown or knockout. We considered genes whose expression decreased with ZFX knockdown/knockout to be “activated” by ZFX, and those whose expression increased to be “repressed” by ZFX. Top row, Chr X-responsive autosomal genes; middle row, Chr Y-responsive autosomal genes; bottom row, Chr X- and Y-responsive autosomal genes. To identify significant enrichments of ZFX target genes in Chr X- or Y-responsive genes, we restricted analyses to genes expressed in both cell lines

(e.g., LCLs and MCF7 cells) and performed hypergeometric tests. Bars marked with an asterisk indicate the P value of the enrichment was lower than the Bonferroni-adjusted threshold of 6.9e-4.

Figure 18

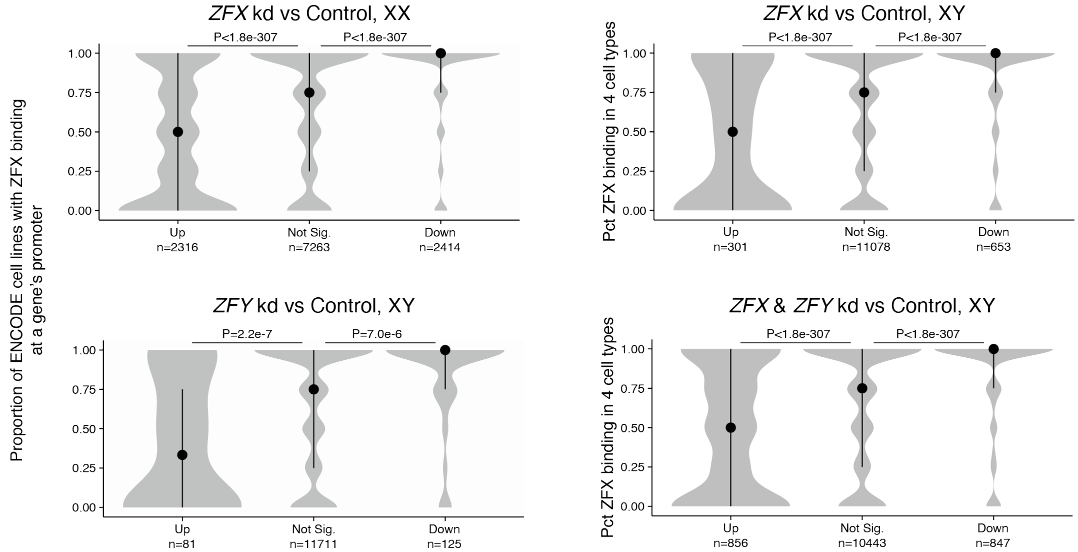

Figure S18. ZFX protein binding is enriched in promoters of autosomal genes that decrease in response to *ZFX* and/or *ZFY* knockdown in fibroblasts, and is depleted in promoters of genes that increase, related to Figure 6. For autosomal genes with or without significant expression changes in each of the four CRISPRi experiments, we investigated ZFX binding using publicly available ChIP-seq data from four cell lines (as in **Fig. S16**). Violin plots, median (dot), and interquartile range (line) of the proportion of cell lines with ZFX binding at the gene's promoter (in cell lines where the gene is expressed). The number of genes in each violin is indicated. Bonferroni-adjusted P values, Wilcoxon rank sum test.

Figure S19

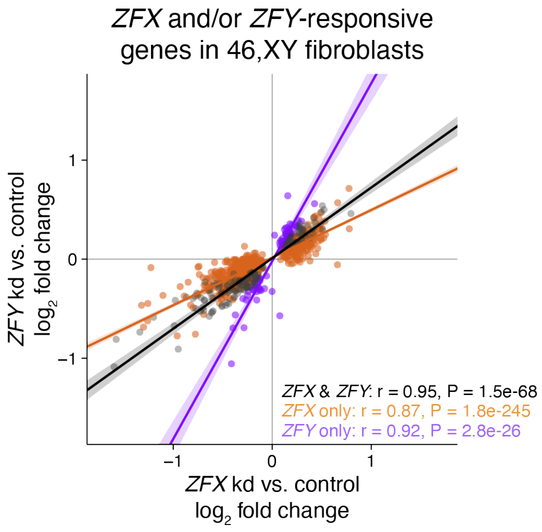

Figure S19. Correlation plots for significantly *ZFX* and/or *ZFY* responsive genes, related to Figure 6. Scatterplot showing the relationship between the effects of *ZFX* and *ZFY* knockdown in 46,XY cells for autosomal genes called as significantly responsive (P_{adj}<0.05) to either *ZFX* (orange), *ZFY* (purple), or both (grey). Weighted Deming regression lines and 95% confidence intervals (solid purple, orange, or black lines with shading), Pearson correlation coefficients, and Bonferroni-adjusted P values of the correlations are included.

Figure S20

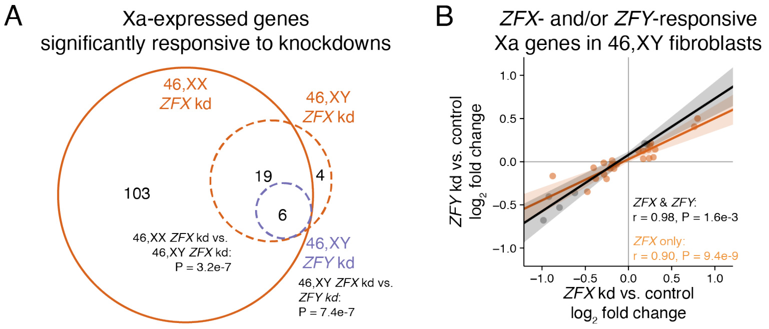

Figure S20. *ZFY*-responsive Xa-expressed genes are also *ZFX*-responsive, related to Figure 6. (A) Venn diagram of Xa-expressed genes significantly differentially expressed upon knockdown of *ZFX* in XX or XY cells or upon knockdown of *ZFY* in XY cells. Bonferroni-adjusted P values, hypergeometric test. (B) Scatterplot of Xa-expressed genes in 46,XY cells that were statistically significantly responsive to both *ZFX* and *ZFY* (grey points) or only *ZFX* (orange points), comparing gene-by-gene effects of *ZFX* versus *ZFY* knockdowns. Weighted Deming regression lines and 95% confidence intervals (black or orange lines and shading); Pearson correlation coefficients and Bonferroni-adjusted P values are indicated.

Figure S21

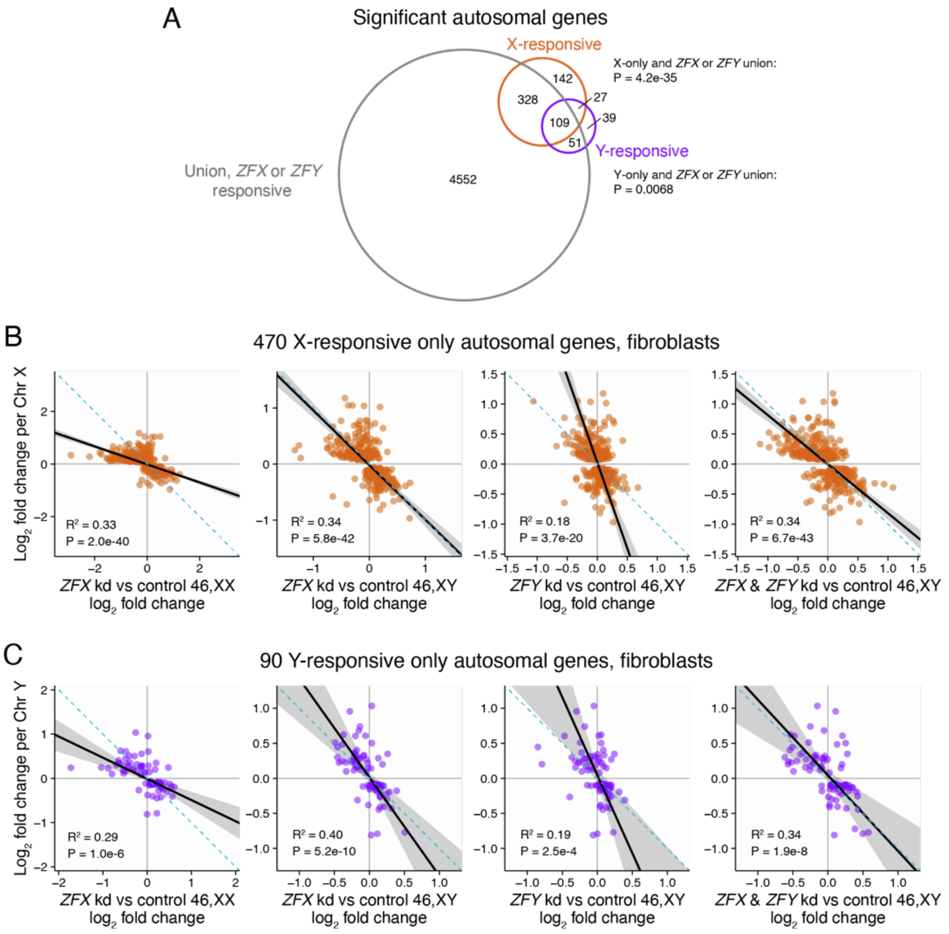

Figure S21. *ZFX* and *ZFY* response also explains variation in X-responsive-only and Y-responsive-only autosomal genes, related to Figure 7. (A) Venn diagram of genes significantly responsive to either Chr X or Y copy number in fibroblasts and the union of genes responsive to *ZFX* or *ZFY* across all four CRISPRi experiments. Bonferroni-adjusted P values, hypergeometric test. **(B-C)** Scatterplots for X-responsive-only (B) or Y-responsive-only (C) genes comparing the effect size in response to Chr X (B) or Chr Y (C) copy number with the effect size in response to four *ZFX* or *ZFY* CRISPRi experiments. Black line and grey shading, weighted Deming regression and 95% confidence interval; blue dashed line, identity line (X=Y). Coefficients of determination and Bonferroni-adjusted P values are indicated.

Figure S22

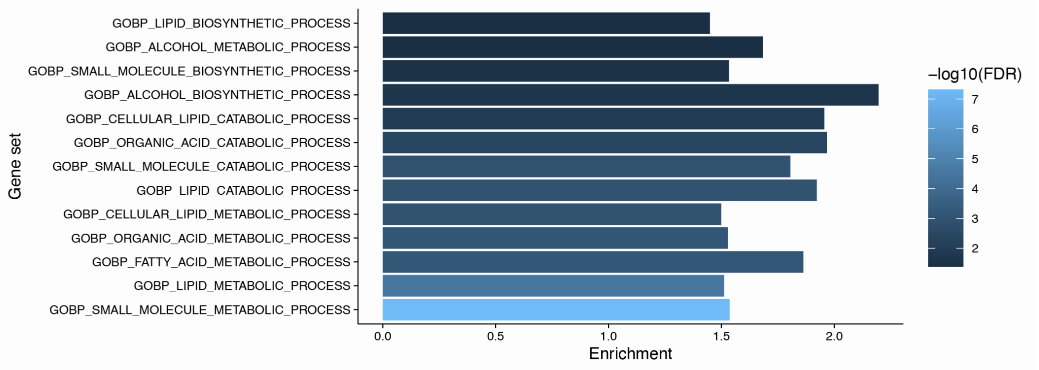

Figure S22. Functional enrichment in union of significant Chr X or Y responsive autosomal and Xa-expressed genes in LCLs and fibroblasts, related to Figure 1. Enrichment of "Biological Process" Gene Ontology categories was calculated for the union of autosomal and Xa-expressed genes and genes that significantly responded to Chr X or Y copy number in LCLs and fibroblasts. The union of all expressed autosomal and Xa-expressed genes in LCLs and fibroblasts was used as the background set. Shown here are categories that fall below the Benjamini-Hochberg-corrected P value (FDR) of 0.05, hypergeometric test.

References

[S1] Rhie, S.K., Yao, L., Luo, Z., Witt, H., Schreiner, S., Guo, Y., Perez, A.A., and Farnham, P.J. (2018). ZFX acts as a transcriptional activator in multiple types of human tumors by binding downstream from transcription start sites at the majority of CpG island promoters. Genome Res. *28*, 310-320. 10.1101/gr.228809.117.

[S2] Ni, W., Perez, A.A., Schreiner, S., Nicolet, C.M., and Farnham, P.J. (2020). Characterization of the ZFX family of transcription factors that bind downstream of the start site of CpG island promoters. Nucleic Acids Res. *48*, 5986-6000. 10.1093/nar/gkaa384.
